# Structural Elucidation and Revised Biosynthetic Pathway of the Membrane Vesicle–Associated Antifungal Compound AFC-BC11

**DOI:** 10.1021/jacs.6c11512

**Published:** 2026-09-06

**Authors:** Inês Tavares, Yi-Chi Chen, Kirsty Agnoli, Michael Berger, Felix Hartrampf, Simon Sieber, Karl Gademann, Leo Eberl

**Affiliations:** † Department of Plant and Microbial Biology, University of Zurich, Zollikerstrasse 107, Zurich CH 8008, Switzerland; ‡ Department of Chemistry, University of Zurich, Winterthurerstrasse 190, Zurich CH 8057, Switzerland

## Abstract

Bacterial membrane vesicles (MVs) serve as delivery vehicles for hydrophobic and membrane-associated secondary metabolites, enhancing their solubility, stability, and bioactivity. Here, we show that the antifungal compound AFC-BC11, produced by *Burkholderia cenocepacia* K56–2, is released via MVs. Using HR-MS/MS, NMR, chemical degradation, and stable isotope feeding experiments, we determined the chemical structure of this compound and analyzed its biosynthesis. Our results confirm that the structure largely matches the recent report by Zhong et al., with a key difference: the double bond in the fatty acid moiety is positioned between C11 and C12. We provide compelling evidence that this constitution reflects the direct incorporation of *cis*-vaccenic acid, one of the most abundant fatty acids in *B. cenocepacia*, rather than a tailoring modification. Comparative analysis of *afcU*, *afcF*, and *afcS* mutants suggests a biosynthetic route involving ω-modification of *cis*-vaccenic acid, which would revise previously proposed pathways and open avenues for acyl chain engineering. Together, these findings establish AFC-BC11 as an MV-associated antifungal metabolite, provide a refined structural and biosynthetic model, and highlight the role of MVs in the dispersal of hydrophobic bioactive compounds.

## Graphical abstract

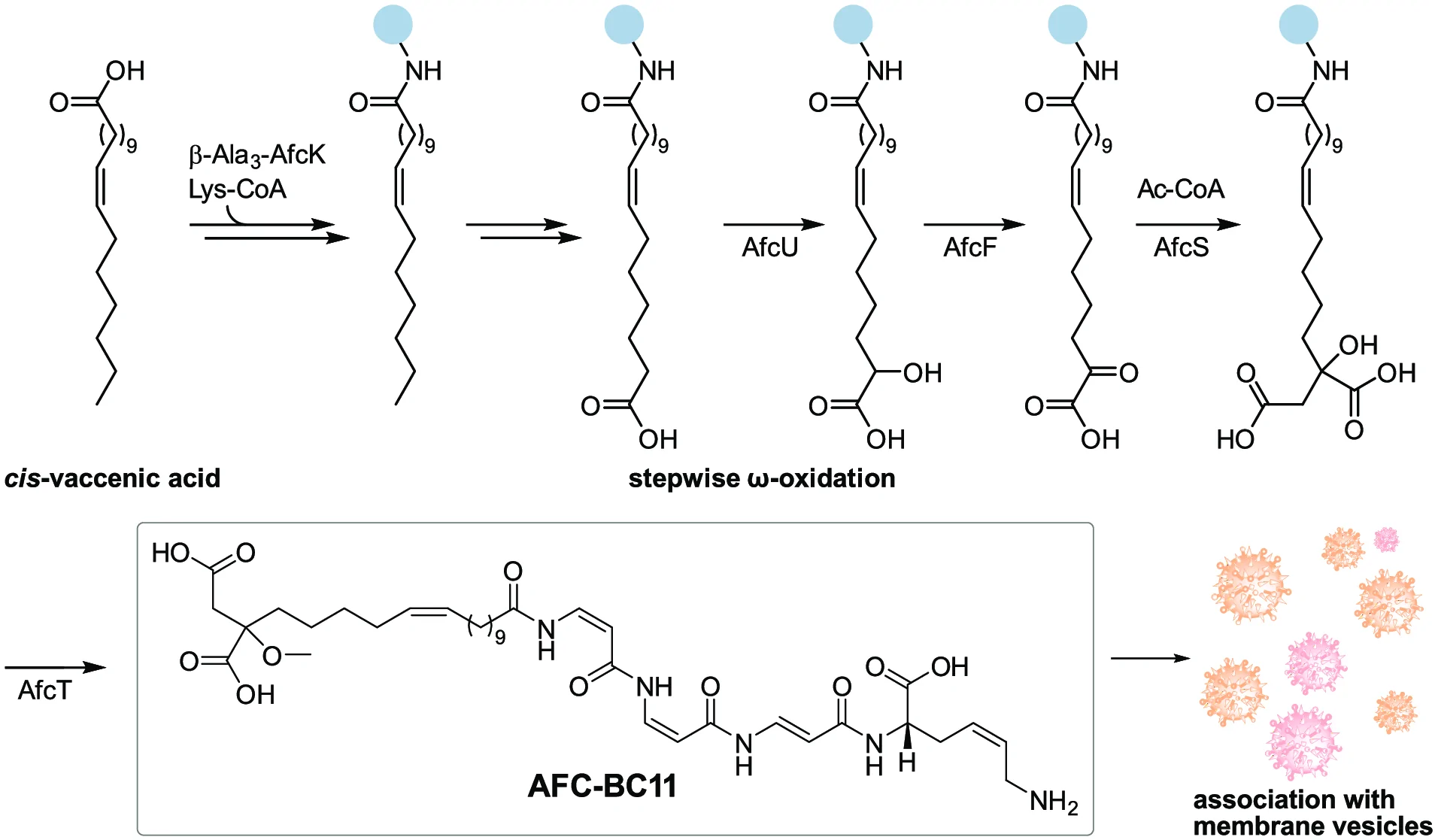

## Introduction

Bacteria employ diverse mechanisms to deliver secondary metabolites to their targets, including protein secretion systems,^1^ membrane transporters,^2^ and membrane vesicles (MVs).^3^ MVs are small, spherical particles (40–400 nm in diameter) derived from the cell envelope that can carry specific cargo.^3^ They arise via different biogenesis routes, such as outer membrane blebbing in Gram-negative bacteria, passage through peptidoglycan pores in Gram-positive bacteria, or cell lysis.^3^ MVs have been proposed to function as carriers for membrane-associated or hydrophobic molecules.^3^ Indeed, several bacterial secondary metabolites have been shown to be MV-associated (Figure 1A). For example, the *Pseudomonas aeruginosa* quinolone signal (PQS) was shown to intercalate into the outer membrane, inducing curvature and driving MV formation via the “bilayer-couple” mechanism, thereby ensuring its own packaging and transport.^4^ Other hydrophobic signaling molecules, such as C16-HSL in *Paracoccus denitrificans* ^5^ and CAI-1 in *Vibrio harveyi*,^6^ follow a similar principle. Beyond signaling, a wide range of bioactive compounds have been shown to be selectively enriched in MVs, including rhamnolipids and hydroxyalkylquinolines in *Burkholderia thailandensis*,^7^ violacein in *Chromobacterium violaceum*,^8^ linearmycins in *Streptomyces* sp. Mg165,^9^ and the bacteriocin micrococcin P1 in *Staphylococcus hominis*.^10^ Strikingly, MV association can increase solubility, stability, and activity of these compounds, as demonstrated for the algicidal tambjamine LY2, which is packaged into MVs by *Chitinimonas prasina* and efficiently delivered to target microalgae.^11^

**Figure 1:**
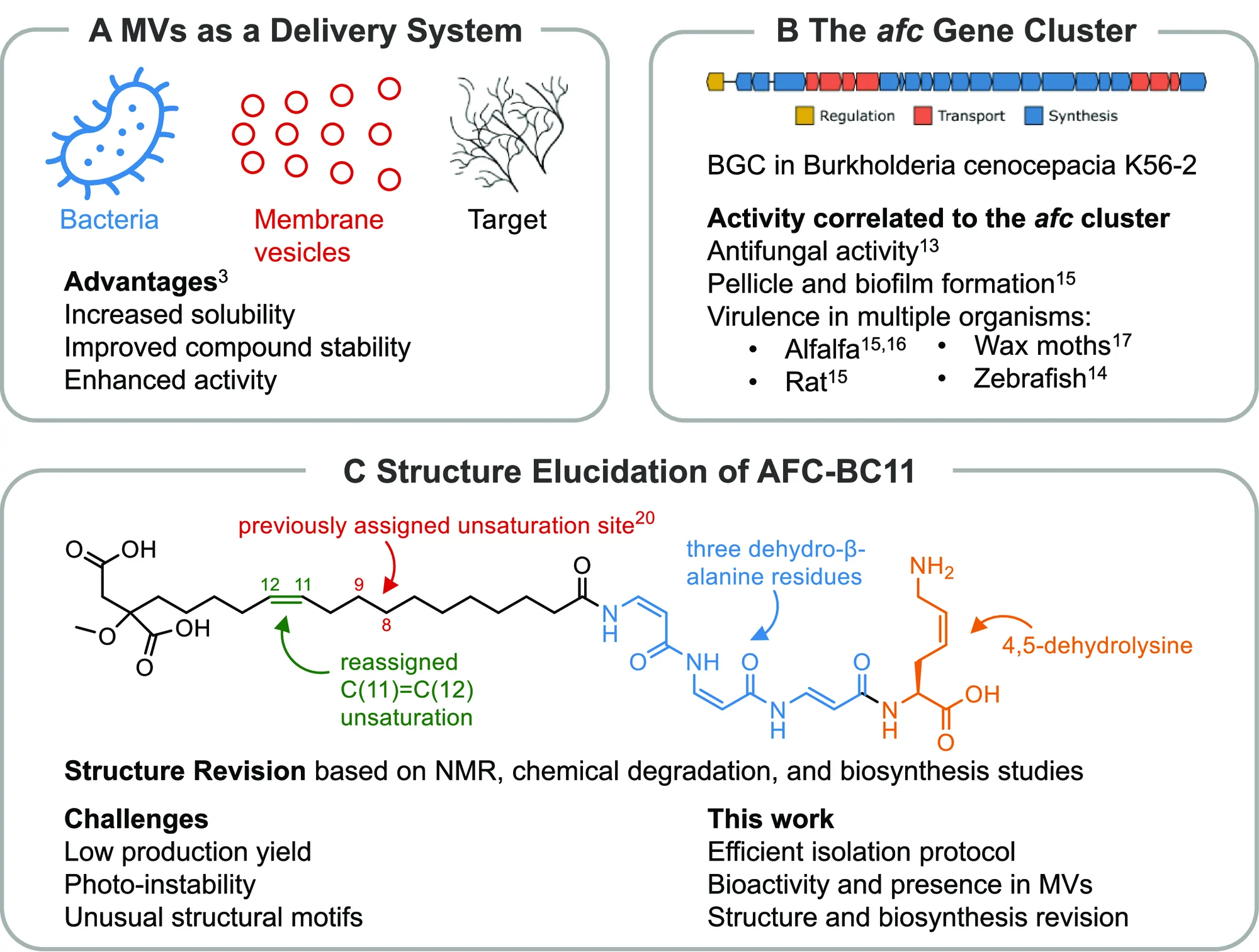
Membrane vesicles (MVs), the *afc* gene cluster, and structure revision of AFC-BC11 (**1**). (A) MVs released by bacteria can act as delivery systems for their associated cargo. MV packaging can confer several advantages to bioactive molecules, including increased solubility and stability, as well as delivery in concentrated doses to the target organisms. (B) The genes responsible for AFC-BC11 production are organized in the *afc* cluster. Initially identified for its role in antifungal activity, expression of the *afc* cluster has also been linked to virulence against a range of organisms and has also been shown to influence processes such as pellicle and biofilm formation. (C) Structure elucidation and revision of AFC-BC11 presented in this study with the fatty acid unsaturation between C(11)=C(12).

Members of the genus *Burkholderia* are well-known for producing a wide range of antifungal compounds.^12^ Among them, *Burkholderia cenocepacia* K56–2 produces the antifungal compound AFC-BC11 (**1**, Figure 1B,1C), which exhibits activity against diverse plant-pathogenic fungi.^13^ Although the chemical structure of AFC-BC11 was initially unresolved, Kang and colleagues described it as a novel, membrane-associated lipopeptide with limited water solubility in 1998.^13^ The corresponding *afc* biosynthetic gene cluster (Figure 1B) has since been linked to pellicle formation, biofilm development, and virulence in multiple model organisms, including alfalfa, rats, wax moths, and zebrafish.^14–17^ Notably, in zebrafish, AFC-BC11 is essential for the transition from intracellular persistence to acute pro-inflammatory infection and its production correlates with increased virulence among closely related strains.^14^

AFC-BC11 is produced by several members of the *Burkholderia sensu lato*, a group of closely related bacteria encompassing seven genera^18^ that inhabit soil, freshwater, industrial environments and a variety of hosts, including plants, fungi, animals and humans.^19^ Homologous *afc*-like gene clusters are also found in bacteria outside the *B. sensu lato*, including in members of the genera *Collimonas, Pseudomonas*, and *Chromobacterium*, although the production of AFC-BC11 or related molecules in these strains have not yet been investigated. Moreover, the *afc* cluster shares gene homology with the bolagladin biosynthetic pathway.^20^ While bolagladins display structural similarities to AFC-BC11, their synthesis involves a large, modular nonribosomal peptide synthase that yields a cyclodepsipeptide scaffold absent in AFC-BC11 (Figure S1).

During a detailed analysis of the cargo of MVs produced by various *Burkholderia* strains, we found that MVs from *B. cenocepacia* K56–2 cultures exhibited strong antifungal activity. We demonstrate here that this activity is linked to AFC-BC11, which is associated with MVs and thereby appear to facilitate its dispersal in aqueous environments. We also determined the chemical structure of AFC-BC11 produced by *B. cenocepacia* K56–2. While this manuscript was in preparation, Zhong and colleagues reported on the structure of the AFC-BC11 from *Burkholderia orbicola* and *Burkholderia puraquae* and provided valuable insights into its biosynthesis.^20^ Our findings are broadly consistent with their results but also complement them by revealing notable differences in both the structure and biosynthetic pathway of AFC-BC11 in *B. cenocepacia* (Figure 1C).

## Results

### *Burkholderia* sp. Release Antifungal Compounds via MVs

To investigate the role of MV-mediated secretion of bioactive molecules, MVs were isolated by ultracentrifugation from cultures of selected *Burkholderia* strains that inhibited the growth of the plant pathogen *Rhizoctonia solani* in dual-culture agar plates assays, as detailed in the Supporting Information. The MVs were then tested for antagonistic activity against *R. solani* and *Bacillus subtilis* (Figures 2A and S2). MVs from *B. thailandensis* E264 and *B. cenocepacia* K56–2 showed activity against both organisms, while MVs from *Burkholderia vietnamiensis* ABIP434 displayed antifungal activity only. The antifungal activity of MVs from strain K56–2 was of particular interest, as this strain is known to produce AFC-BC11.^13^ Given previous descriptions of AFC-BC11 as a poorly water-soluble molecule with antifungal activity,^13^ we hypothesized that the MV-associated activity of K56–2 was attributable to AFC-BC11. To test this, MVs were isolated from both wild-type K56–2 and a mutant strain (Δ*afc*) lacking the entire *afc* biosynthetic cluster and their antifungal activities were compared (Figure 2A). The complete loss of antagonistic activity against both organisms in the *afc* mutant suggests that AFC-BC11 was responsible for the activity observed in the MV fraction. To confirm the presence and morphology of membrane vesicles, the MV fraction from K56–2 WT was examined by transmission electron microscopy (TEM) (Figure 2B). The preparations were predominantly composed of vesicle-like structures, with flagella being the principal nonvesicular species observed, and no substantial membrane debris or protein aggregates were detected. Nanoparticle tracking analysis (NTA) was additionally used to compare the concentration and size distribution of MVs produced by K56–2 WT and the *afc* mutant. No significant differences in MV production or size distribution were observed between the two strains (Figure 2C). Although TEM and NTA cannot formally exclude the presence of minor amounts of cosedimenting membrane fragments, the TEM analysis provided no evidence for substantial particulate contamination of the MV preparations.

**Figure 2:**
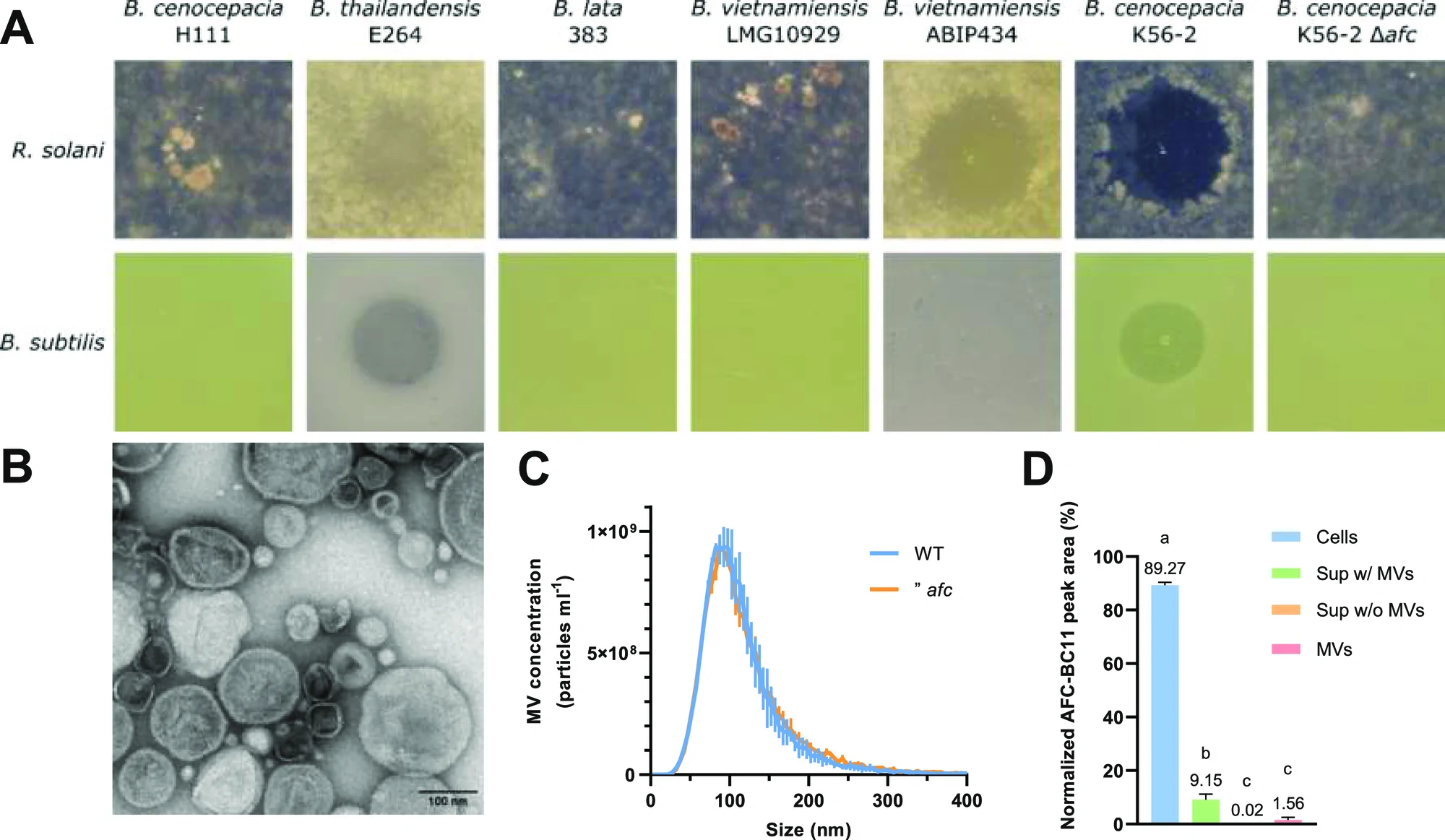
Antifungal and antibacterial activity of MVs from *Burkholderia* strains, characterization of membrane vesicles, and quantification of AFC-BC11 produced by *B. cenocepacia* K56–2. (A) Antagonistic activities of MVs isolated from selected *Burkholderia* strains against *R. solani* (top) and *B. subtilis* (bottom). MVs from the indicated strains were collected by ultracentrifugation. To test activity against *R. solani*, MVs were spotted onto MEA (malt extract agar) plates, which were subsequently overlaid with a suspension of disrupted *R. solani* mycelium. To test for activity against *B. subtilis*, MVs were spotted on a *B. subtilis* lawn on LB plates. Antimicrobial activity was observed as a halo corresponding to a zone of growth inhibition. (B) Transmission electron microscopy (TEM) images of MVs isolated from culture supernatants of *B. cenocepacia* K56–2 WT. (C) Concentration and size distribution of MVs isolated from culture supernatants of *B. cenocepacia* K56–2 WT and the Δ*afc* mutant as determined by nanoparticle tracking analysis (NTA). Data are presented as mean ± SD from three biological replicates. (D) Quantification of AFC-BC11 in different fractions of a *B. cenocepacia* K56–2 cultures grown in PDB. The total amount AFC-BC11 was quantified in the cells, culture supernatant (before and after removal of MVs) and isolated MVs. Data is presented as mean ± s.d. from biological triplicates. Different letters indicate statistically significant differences (*p* ≤ 0.005) according to one-way ANOVA followed by Tukey posthoc test. The nonsignificant result between “Supernatant w/o MVs” and the “MVs” samples was due to the experimental error introduced because of loss of sample in MV purification.

Quantification of AFC-BC11 in a K56–2 culture further showed that the majority of the molecule is retained within cells, while extracellular AFC-BC11 present in the supernatant is predominantly associated with the MV fraction (Figure 2D). Given the unusual solubility properties of AFC-BC11, its association with membrane vesicles likely facilitates its release and dispersal into the surrounding aqueous environment. Although a minor contribution from other membrane material cannot be formally excluded, the data support the conclusion that AFC-BC11 is predominantly associated with the MV fraction. Although the association with MVs likely increases the dispersal of AFC-BC11, it is not required for bioactivity, as the purified molecule also exhibited antifungal activity.^20^

### AFC-BC11 Can Be Isolated by a Novel Extraction Method

Determining the structure of AFC-BC11 has been a longstanding challenge, with nearly 25 years elapsing between its initial identification and significant advances in its chemical characterization, despite substantial progress in elucidating its biological activities during this period. The major obstacles in isolating this compound have been its poor solubility in both aqueous and common organic solvents and its instability. To overcome this, we established a novel and straightforward protocol that enabled efficient purification of AFC-BC11. In brief, whole K56–2 cultures after 24 h of growth were harvested without centrifugation and directly extracted with chloroform/methanol (1:2). The biphasic, heterogeneous mixture was then centrifuged (4000*g*), and both the aqueous and organic layers were discarded; only the precipitate was retained. The resulting precipitate was subsequently dissolved in DMF containing 1% formic acid. The yield and purity of AFC-BC11 using this extraction protocol were compared to AFC-BC11 obtained with an alternative protocol described in Zhong et al.^20^ (Figure S3). Our procedure facilitated the efficient recovery of target compound **1**. Because both water- and organic-soluble impurities were removed, the extract was already highly pure (Figure S3). Final purification was achieved by HPLC for downstream structural and functional analyses, as detailed in the Supporting Information.

### Structure Elucidation of AFC-BC11

Purified AFC-BC11 (**1**) was subjected to ultrahigh performance liquid chromatography coupled with electrospray ionization high-resolution mass spectrometry (UHPLC-ESI-HRMS) analysis using a timsTOF spectrometer. A single peak with *m*/*z* of 734.3965 (Δppm = 0.79) was observed (Figure 3A–D). The HR-MS/MS spectrum displayed major ions corresponding to the fragments in the peptide part of the molecule, generating fragments that are a combination of three dehydro-β-alanines (DBA) and a dehydrolysine (DHLys) residue (Figure 3E,F). Interestingly, fragments α, β and γ observed in MS/MS indicated that AFC-BC11 (**1**) might have lost one, two or three DBA moieties, respectively. In addition, the fragmentation of the aliphatic side chain with one DBA subunit (ion b_1_, calculated *m*/*z* of 452.2643) was obtained by a pseudo-MS^3^ measurement via Q Exactive Orbitrap mass spectrometer (Figure S4), showing several fragments with a combination of *O*-methylation, carboxylic acid, or methylcarboxylic acid loss, which confirmed the *O*-methylated malic acid-fatty acid (MMFA) motif.

**Figure 3:**
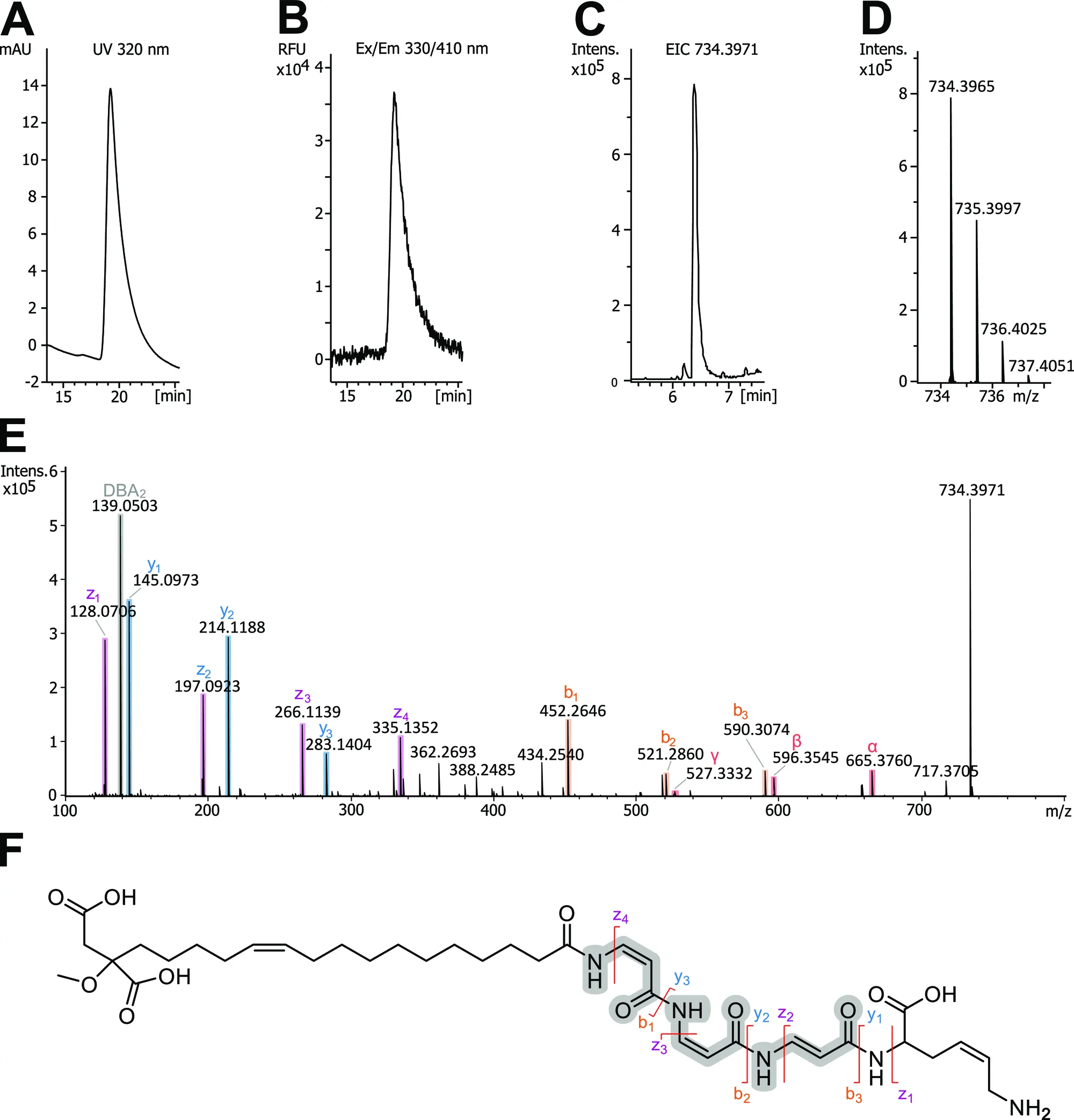
Identification of purified AFC-BC11 by UHPLC, UHPLC-HR-MS/MS and proposed assignment of selected fragments. Extracts of UHPLC chromatogram of (A) UV absorption at 320 nm and (B) fluorescence detection with λ_ex_/λ_em_ = 330/410 nm. (C) Extracted ion chromatogram for AFC-BC11. (D) Isotopic pattern of the [M + H]^+^ ion of AFC-BC11. (E) HR-MS/MS of AFC-BC11. Possible fragments involving the loss of one, two or three DBA units are labeled in red with α, β or γ, respectively. (F) Fragment assignment, DBA subunits are highlighted in light gray.

The structure was further analyzed by a combination of NMR techniques, including 1D and 2D experiments (Figures S5–S13 and Table S1). ^1^H–^1^H TOCSY experiments revealed that the compound was composed of five spin systems, including four modified amino acids and a fatty acid derivative with an unsaturated bond. The connectivity between the different parts of AFC-BC11 (**1**) was assigned using the correlations observed in the ^1^H–^13^C HMBC and ^1^H–^1^H NOESY spectra (Figure S14). Further analysis by ^1^H–^1^H COSY, ^1^H–^13^C HSQC, ^1^H–^13^C HMBC experiments led to the identification of a modified lysine residue with a C(4)=C(5) double bond (DHLys) and three DBA units. These amino acids possessed similar chemical shifts to those reported for bolagladins.^21,22^ A detailed analysis of the vicinal scalar coupling constants indicated that the double bond of the DHLys residue adopts a *cis* configuration (^3^ *J* = 11.0 Hz), the DBA^1^ fragment is *trans*-configured (^3^ *J* = 13.9 Hz), and the DBA^2^ and DBA^3^ units are *cis*-configured (^3^ *J* = 8.9 Hz and ^3^ *J* = 8.8 Hz, respectively). The configuration of the DHLys stereocenter was tentatively assigned as *S*, based on the recent Marfey’s amino acid analysis reported by Zhong and co-workers.^20^ Structural elucidation of the fatty acid side chain was achieved using a combination of ^1^H–^1^H COSY, ^1^H–^1^H TOCSY, ^1^H–^13^C HSQC, ^1^H–^13^C HMBC, and ^1^H–^13^C HSQC-TOCSY spectroscopy. Notably, the two carbon chemical shifts of C18 and C20 (174.0 and 172.1 ppm) could be identified but not differentiated. To obtain further high-quality data, the compound was analyzed on a Bruker AVANCE NEO 1.2 GHz NMR spectrometer. Clear separation between the protons H10 (1.97 ppm) and H13 (1.95 ppm; Figures S15 and S16) was observed. In addition, a high-resolution ^1^H–^13^C HSQC spectrum focusing on the methylene region enabled assignment of C10 and C13 (Figure S17) and the high-resolution ^1^H–^1^H CLIP-COSY spectrum (Figure S18) revealed clear correlations between H9 and H10, H13 and H14, and H15 and H16. Finally, correlations observed in the ^1^H–^13^C HSQC-TOCSY spectra recorded with mixing times of 60 and 100 ms allowed the assignment of the proton and carbon atoms at positions 14 and 15 (Figures S19 and S20). These results were further corroborated by analysis of an AFC-BC11 (**1**) sample obtained from cultures grown in Lysogeny Broth (LB) medium instead of Potato Dextrose Broth (PDB) (see section below). Unlike the PDB-derived samples, the LB samples included an inseparable coeluting impurity, which facilitated the analysis. A full characterization was also performed on this sample (Figures S21–S29 and Table S2) and a significant shift was observed for the proton at position 16 (now at 1.69 ppm, previously 1.57–1.58 ppm). This enabled a clear differentiation of the positions 2, 3, and 16, and revealed a distinct correlation in the ^1^H–^1^H TOCSY spectrum between the protons at position 16 with those at positions 12 and 13, and a ^1^H–^13^C HSQC-TOCSY spectrum between the proton at position 13 and 15. These results indicated that the double bond is located between carbons 11 and 12. To further investigate the location of the double bond, oxidative cleavage of AFC-BC11 (**1**) was carried out by ozonolysis, followed by oxidative workup with H_2_O_2_. The analysis of the cleavage products by UHPLC-HR-MS/MS resulted in the detection of 2-methoxyhexane-1,2,6-tricarboxylic acid, which unambiguously determined the location of the double bond between carbon atoms 11 and 12 (Figures S30–S33). The configuration of this double bond was assigned as *cis* based on a vicinal coupling constant of 11.8 Hz, determined from a ^1^H–^13^C HSQC spectrum acquired without ^13^C decoupling and by selective ^1^H homodecoupling at 1.96 ppm on the AFC-BC11 pure sample (Figures S34 and S35).

In the initial report on the AFC-BCC (**1**) structure, the double bond was assigned between C8 and C9.^20^ As that study employed *B. puraquae* DSM 103137 as the producing strain, the discrepancy in double bond position was initially attributed to strain variation. To clarify this, AFC-BC11 was isolated from *B. puraquae* DSM 103137 following the protocol of Zhong et al. and analyzed by UHPLC-HR-MS/MS.^20^ The compound displayed an identical retention time, confirmed by spiking experiments, and indistinguishable fragmentation patterns (Figure S36). In addition, comparative analysis of the ^1^H NMR and ^1^H–^13^C HSQC spectra confirmed the identity of the two samples (Figures S37 and S38). This result was further corroborated by the formation of the same degradation products upon ozonolysis and oxidative cleavage of AFC-BC11 (**1**) produced by *B*. *puraquae* DSM 103137 (Figures S39). Collectively, these findings indicate that the double bond resides between C11 and C12, thereby necessitating a structural reassignment of AFC-BC11 from *B. puraquae* DSM 103137.

### Environmental Conditions Shape the Generation of Diverse AFC-BC11 Analogs

A closer evaluation of the extract revealed that *B. cenocepacia* K56–2 produces AFC-BC11 as a major product, but several other analogs with modified acyl side chains were also detected. Furthermore, variations in media composition significantly affected the detected intensities of those compounds. The structures of those analogs might provide evidence for the biosynthetic pathway of the AFC class of compounds. To evaluate the role of the growth medium, we profiled the AFC-BC11 and related AFCs produced by *B. cenocepacia* K56–2 grown in liquid LB and PDB media. Major AFC analogs were identified based on their HRMS and their conserved MS/MS fragmentation patterns, including the characteristic DBA_3_-DHLys fragments, and their structures were proposed. These compounds differed in the acyl moiety, with the functional group at the end of the side chain varying from a carboxylic acid (ω-carboxylic fatty acid (ω-CFA)), α-hydroxy ω-CFA, α-oxo ω-CFA, to an α-amino ω-CFA, or MMFA. The chain length of the fatty acid moiety was found to be 16 or 18 carbons, with the longer one possessing a *cis*-configured double bond (Figures 4 and S40–S44). Comparison of the MS peak areas showed that PDB medium predominantly supported the production of AFC-BC11 (**1**, 83.7%) and AFC-BC11–707 (**2**, 6.5%). In contrast, extract of LB cultures displayed a more complex profile, with a marked increase in ω-CFA anologs, specifically AFC-BC11–645 (**3**, 26.0%) and AFC-BC11–619 (**4**, 18.7%) (Figure 5A, **top panel**). Additional minor derivatives were also detected, including the α-hydroxy ω-CFA compounds AFC-BC11–661 (**5**) and AFC-BC11–635 (**6**), the α-oxo ω-CFA analogs AFC-BC11–659 (**7**) and AFC-BC11–633 (**8**), and the α-amino ω-CFA compounds AFC-BC11–660 (**9**) and AFC-BC11–634 (**10**), with structures predicted by MS/MS comparative analysis (Figures S40–S44).

**Figure 4:**
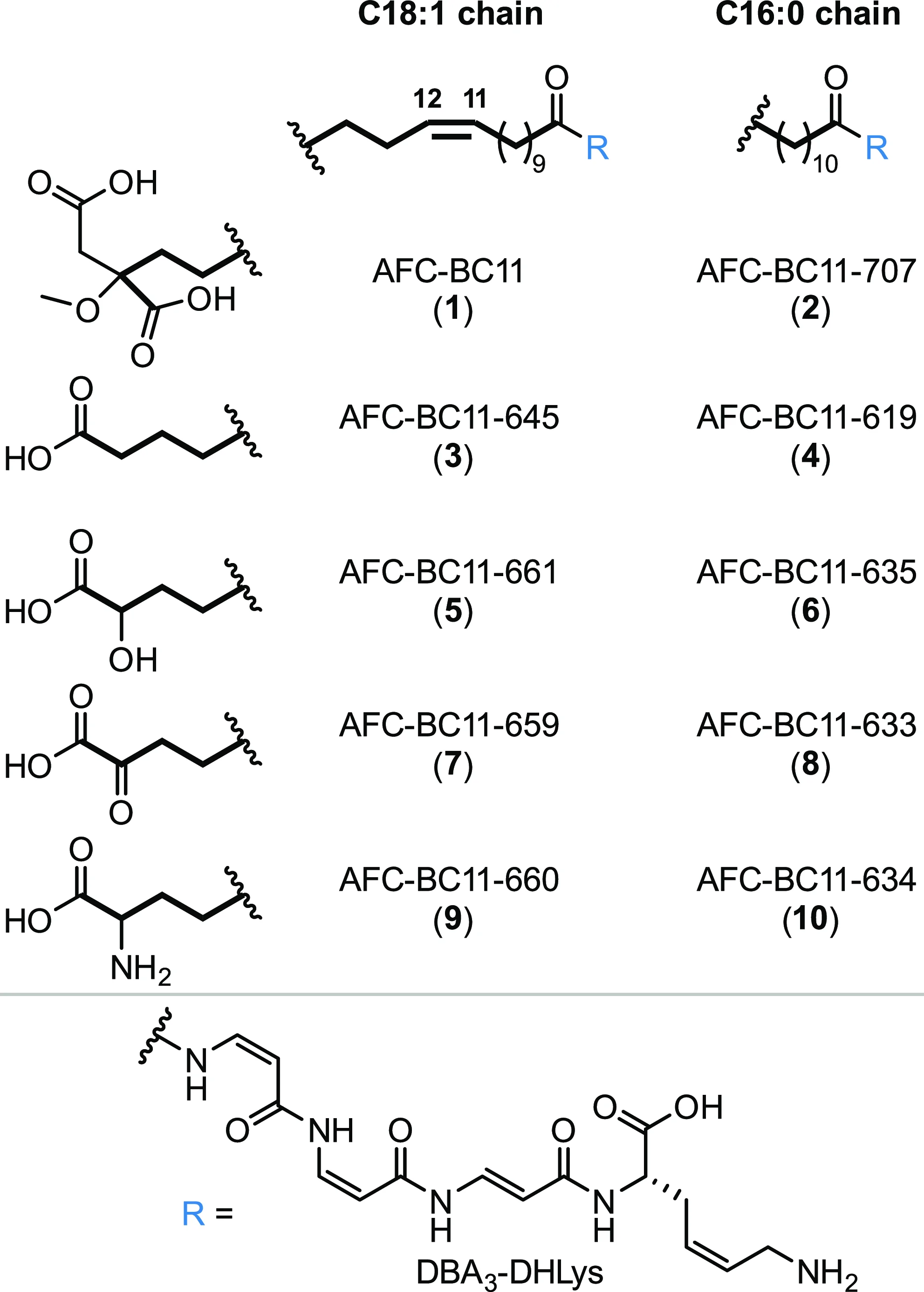
Structure of major AFC products. All share the same core and peptide structure (DBA_3_-DHLys) but differ in acyl chain composition and terminal modification. AFCs are grouped according to acyl chain (C(18):1 or C16:0), the precursor fatty acid chain is highlighted in bold.

**Figure 5:**
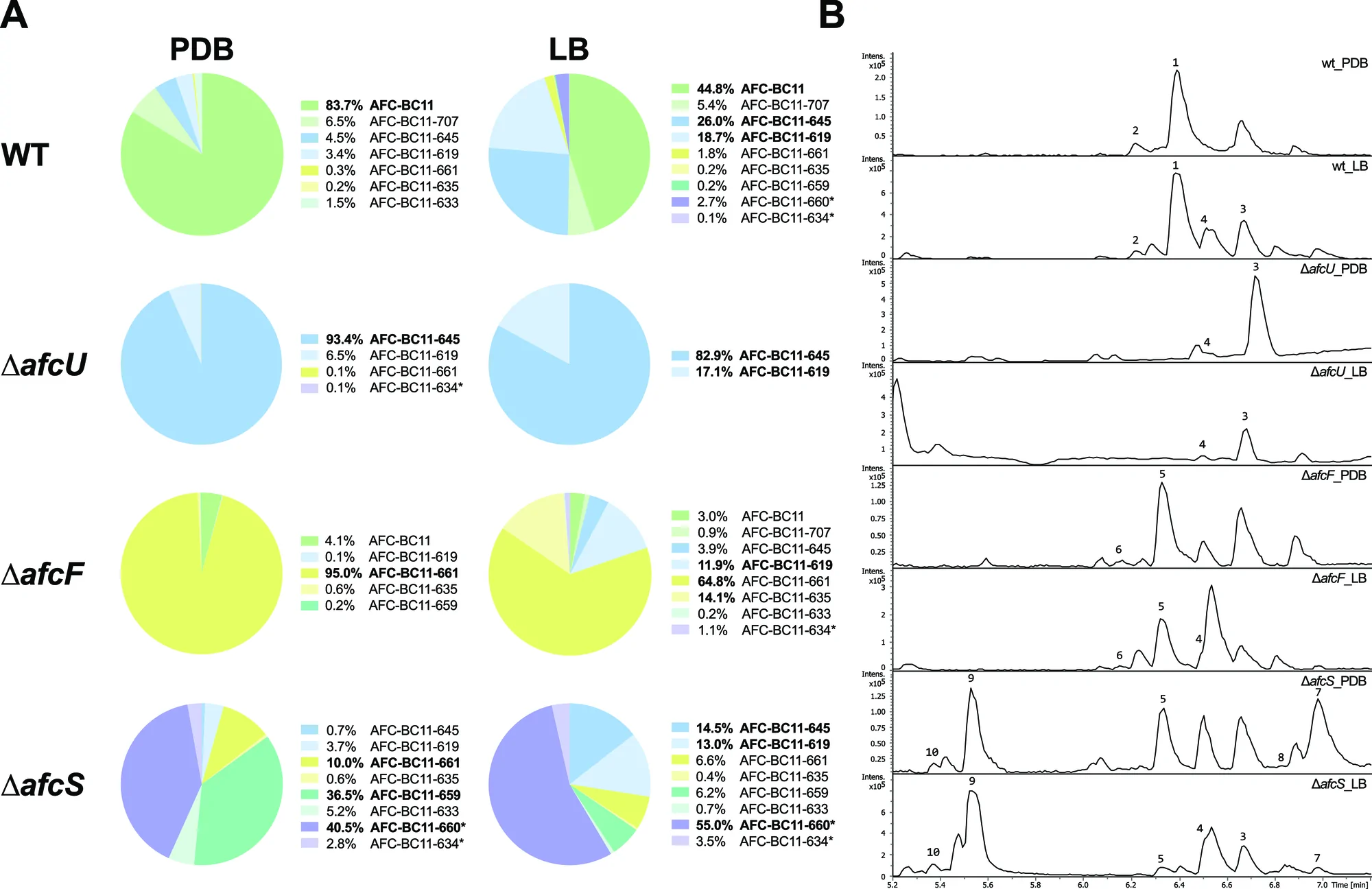
Abundance of major AFCs produced by wild-type, Δ*afcU*, Δ*afcF* and Δ*afcS* strains grown in LB and PDB media as determined by MS. (A) Peak areas of product ion ([M + H]^+^ and [M+2H]^2+^) (**1** to **10**) are integrated. Compounds with relative abundances greater than 10% are shown in bold. *Note: AFC-BC11–660 (**9**) and AFC-BC11–634 (**10**) predominantly form double-charged ions, so their absolute quantities may differ from those of the other AFCs. (B) Base peak chromatograms of AFCs produced by wild-type, Δ*afcU*, Δ*afcF* and Δ*afcS* strains grown in PDB and LB media. AFC-BC11 and its derivatives are indicated by compound numbers **1** to **10**.

These comparisons were based on relative signal intensities obtained from UHPLC-MS analyses in positive ionization mode and should therefore be regarded as semiquantitative, as differences in ionization efficiencies between compounds cannot be excluded. Nevertheless, the conserved presence of the terminal DHLys amino group is expected to contribute to broadly similar ionization behavior among the analyzed AFC derivatives.

### Biosynthesis of the Fatty Acid Moiety of AFCs

It has been proposed that the fatty acid side chain in AFC-BC11 (**1**) is incorporated by initial conjugation to the DBA moieties via an amide bond formation, followed by terminal modification of the acyl side chain through citric acid conjugation.^20^ In contrast, the biosynthesis of bolagladins is thought to involve elongation of the fatty acid side chain directly on a citric acid-derived residue, catalyzed by fatty acid synthase (FAS)^21,22^ (Figure S45). To investigate substrate specificity and clarify the biosynthetic route, stable isotope labeling experiments were conducted using deuterium-labeled fatty acid precursors in PDB culture, and the resulting products were analyzed by UHPLC-HR-MS (Table S3). While in vitro assays had previously indicated that AfcA shows high activity toward myristic acid,^20^ MS analysis of cultures supplemented with deuterium-labeled myristic acid (myristic acid-*d*_27_) only led to the identification of unlabeled AFC-BC11 (Figure S46). Instead, ω-CFA myristic acid derivatives AFC-BC11–591 (**11**) or **11**-*d*_24_ (**12**) were detected. In addition, trace amounts of AFC analogs with a shorter acyl side chain (14 carbon atoms) AFC-BC11–679 (**13**) and **13**-*d*_22_ (**14**) were observed (Figure S46). However, the putative α-hydroxy ω-CFA myristic acid derivatives AFC-BC11–607 (**15**) and **15**-*d*_23_ (**16**) were not detected. Those results suggest that myristic acid is accepted by AfcA, subsequently ω–oxidized, and finally modified to the MMFA product.

Furthermore, supplementation with palmitic acid (C16:0) and *cis*-vaccenic acid (C18:1ω7) resulted in the successful production of MMFA AFCs (Figures S47–S48). Deuterium-labeled palmitic acid (palmitic acid-*d*_31_) led to the detection of the final product AFC-BC11–707-*d*_26_ (**17**) that eluted at the same retention time as unlabeled AFC-BC11–707 (**2**) (Figure S47), and the ω-CFA intermediate AFC-BC11–619-*d*_28_ (**18**). The abundance of AFC-BC11–707-*d*_26_ (**17**) increased with higher concentrations of labeled substrate, supporting conversion from ω-CFA AFCs to MMFA AFCs. Similarly, treatment with *cis*-vaccenic acid-*d*_13_ resulted in the incorporation of deuterium to form AFC-BC11-*d*_8_ (**19**) (Figure S48), consistent with the AfcA-mediated direct incorporation model proposed by Zhong et al.^20^ Importantly, *cis*-vaccenic acid is likely a major substrate for AFC biosynthesis, consistent with the identification of AFC-BC11 (**1**) as the predominant product containing a *cis*-vaccenic acid side chain with a double bond at C(11)=C(12). Supplementation of the medium with unlabeled stearic acid (C18:0) also enabled the identification of MMFA AFC-BC11–735 (**20**) by MS/MS, although only in trace quantities (Figure S49). This result demonstrates that stearic acid can be incorporated into the final AFC product. However, its uptake from the culture medium is likely inefficient due to low solubility. In summary, these findings indicate that AfcA can utilize multiple fatty acid substrates. Although *cis*-vaccenic acid appears to be preferentially incorporated under the tested conditions, it remains unclear whether this reflects intrinsic substrate specificity of AfcA or simply the high intracellular abundance of *cis*-vaccenic acid in *Burkholderia* species relative to other fatty acids.

### AfcS, AfcF, and AfcU are Involved in Fatty Acid Moiety Modification

To further investigate the biosynthesis of the fatty acid moiety, we generated unmarked deletion mutants of several genes in the *afc* cluster and analyzed their metabolic products by UHPLC-HR-MS, along with their antifungal activity (Table S4). Metabolic network analysis of AFC derivatives (Figures 5 and S50–S52) revealed that the main metabolites detected in the *afcS* mutant varied in the length of their acyl side chain, being either based on C16:0 or C18:1, and lacked the citric acid moiety. Instead, the aliphatic side chain terminated either with a ω-CFA (AFC-BC11–645 (**3**) and AFC-BC11–619 (**4**)), an α-hydroxy ω-CFA (AFC-BC11–661 (**5**) and AFC-BC11–635 (**6**)), an α-oxo ω-CFA (AFC-BC11–659 (**7**) and AFC-BC11–633 (**8**)), or an α-amino ω-CFA (AFC-BC11–660 (**9**) and AFC-BC11–634 (**10**)). These results are consistent with a recent study by Zhong et al., which showed that AFC-BC11–645 (**3**) and AFC-BC11–661 (**5**) were produced both by the wild type and the *afcS* mutant.^20^ Similar findings were reported for bolagladin biosynthesis:^21,22^ mutation of *bolR*, the homologue of *afcS*, likewise resulted in truncated derivatives possessing either a ω-CFA or an α-hydroxy ω-CFA group. In the *afcS* mutant, α-oxo ω-CFA AFCs **7** and **8** and α-amino ω-CFA **9** and **10** were also detected, suggesting that disruption of acetylation in this mutant leads to the accumulation of α-oxo ω-CFA AFCs (Figure 5), which may subsequently be converted into α-amino ω-CFA AFCs, possibly by reductive amination.

Metabolic profiling of the *afcF* mutant revealed it produced derivatives possessing ω-CFA (**3** and **4**) and α-hydroxy ω-CFA (**5** and **6**). The compounds **3** and **5** were likewise observed in the *afcF* mutant of *B. pyrrocinia* DSM 10685 ^20^. Since the α-oxo ω-CFA **7** and **8** were observed only in Δ*afcS* and not in Δ*afcF*, we hypothesize that AfcF catalyzes the oxidation of the α-hydroxy ω-CFA AFCs into the α-oxo ω-CFA compounds. The *afcU* mutant produced only the ω-CFA **3** and **4**, strongly suggesting its role in the initial modification step of the ω-CFA (Figure 5). Based on these findings, we propose that the biosynthesis of the AFC fatty acid moiety proceeds via ω-modification of the fatty acid, a pathway distinct from the previously suggested routes reported for AFC-BC11­(**1**) and bolagladins (Figures S45 and 6).^20–22^

**Figure 6:**
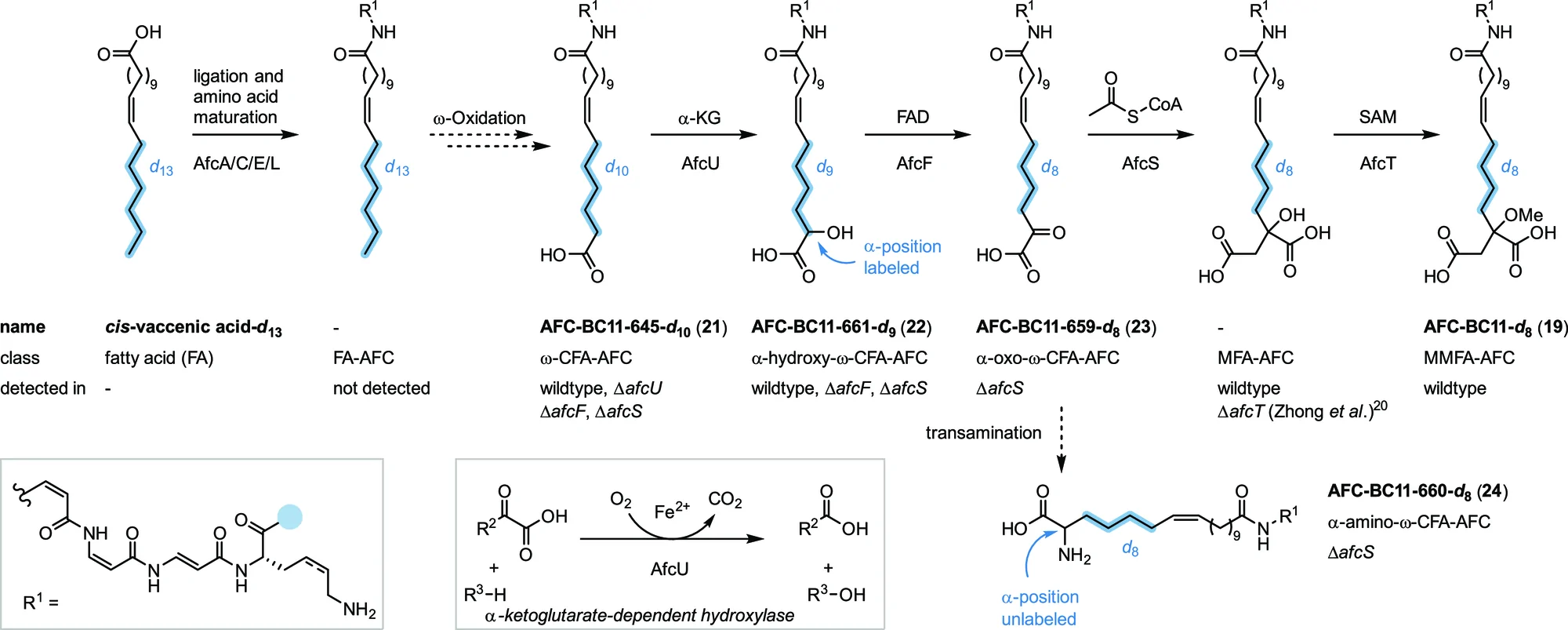
Proposed biosynthetic pathway of AFC-BC11. The biosynthesis of AFC-BC11 is clarified through the stepwise transformation of deuterium-labeled (blue) *cis*-vaccenic acid into deuterium-labeled AFC-BC11 (**19**). Proposed, but not experimentally validated steps are indicated with dashed arrows. The exact timing of lysine maturation and peptide release remains unknown and is represented with a generic formula R^1^ and a blue sphere for either CoA or OH. A mechanism for AfcU-catalyzed α-hydroxylation is proposed by comparing the reaction with TauD from *Escherichia coli* K12 and *P. putida* KT2442 (see insert).^27,28^ α-KG, α-ketoglutarate; CoA, coenzyme A; FA, fatty acid; MFA, malic acid-fatty acid; MMFA, methylated malic acid-fatty acid; SAM, *S*-adenosyl methionine.

To test this model, we supplied deuterium-labeled and unlabeled *cis*-vaccenic acids and tracked the intermediates in the wild type and the *afcU*, *afcF*, and *afcS* mutants (Figure S53). Several deuterium-labeled adducts, including AFC-BC11-*d*_8_ (**19**), AFC-BC11–645-*d*_10_ (**21**), AFC-BC11–661-*d*_9_ (**22**), AFC-BC11–659-*d*_8_ (**23**), and AFC-BC11–660-*d*_8_ (**24**), were detected (Figures S53 and S54 and Table S3). A stepwise decrease in deuterium labeling was observed from the substrate *cis*-vaccenic acid-*d*_13_ to the final product AFC-BC11-*d*_8_ (**19**). After the incorporation of the peptide domain via AfcA, the fatty acid terminus is presumably oxidized to form AFC-BC11–645-*d*_10_ (**21**) (Figure 6). No intermediates of the ω-oxidation carrying an OH or CHO group were detected, likely due to rapid turnover or instability. Subsequently, AfcU is suggested to introduce an α-hydroxy group, evidenced by the observation of loss of one deuterium atom from AFC-BC11–645-*d*_10_ (**21**) to AFC-BC11–661-*d*_9_ (**22**, Figures 6 and S53). Further oxidation by AfcF converted the OH group into the corresponding carbonyl group, deduced from an additional deuterium loss to yield AFC-BC11–659-*d*_8_ (**23**) (Figures 6 and S53). The aminated product AFC-BC11–660-*d*_8_ (**24**) was observed in the *afcS* mutant but not in Δ*afcF*, indicating that the α-amino group is derived from the α-oxo group rather than the α-hydroxy group, likely via transamination catalyzed by an unknown enzyme (Figures 6 and S53). Finally, since the α-oxo ω-CFA product is a structural mimic of oxaloacetate, the carboxymethyl group was likely incorporated from acetyl-Coenzyme A (acetyl-CoA) via AfcS (Figure 6). These findings support our hypothesis that AFC-BC11 biosynthesis proceeds via ω-modification.

## Discussion

While the major steps in the assembly of the peptide components of both AFC-BC11 and bolagladins have been well resolved, the exact chemistry underlying the addition and modification of the fatty acid remains enigmatic.^20–22^ Apart from the additional 2,3-double bond in the bolagladins, the fatty acid side chains of AFC-BC11 and bolagladins are structurally identical, and the enzymes involved in their biosynthesis display a high degree of homology (53–84%). Nevertheless, two fundamentally different biosynthetic pathways have been proposed for the formation of the fatty acid moieties in AFC-BC11 and bolagladins.

In the case of bolagladins, it has been suggested that the fatty acid residue originates from an unusual citryl-CoA starter unit.^21,22^ The biosynthesis would continue by extending this moiety with the addition of a malonyl group, which would be bound to the acyl carrier protein (ACP) of the fatty acid synthase (FAS).^22^ Further expansion of this fragment would yield the saturated MMFA-ACP thioester (Figure S45), which would be finally oxidized to form two double bonds (C(2)=C(3) and C(11)=C(12)).^21,22^ In the case of AFC-BC11, it has been proposed that AfcA catalyzes the activation and subsequent transfer of the myristic acid to the terminal β-amino group of BA_3_–Lys–SCoA (Figure S45).^20^ The fatty acid is then conjugated to citric acid by undetermined enzymes to generate the AFC-BC11. However, the mechanism responsible for introducing the double bond in the fatty acid has not been elucidated.

We suggest that AfcA transfers a fatty acid to a peptide derivative, but we propose that *cis*-vaccenic acid, rather than myristic acid, may serve as the precursor. This interpretation is based on both our feeding experiments and the fact that *cis*-vaccenic acid is one of the most abundant fatty acid in *B. cenocepacia.* ^23,24^ Notably, this could also explain the C(11)=C(12) double bond in the fatty acid residue without requiring an additional enzyme for its introduction. The position of this double bond is identical to that in bolagladins, suggesting that *cis*-vaccenic acid may likewise serve as a precursor for their biosynthesis. Substrate assays with purified AfcA showed the highest enzymatic activity with myristic acid.^20^ However, *cis*-vaccenic acid was not included among the tested substrates. Since myristic acid is present only in trace amounts in *Burkholderia sensu lato*,^25^ our data suggest that this fatty acid is taken up by the cell and rapidly converted into ω-carboxylic myristic acid-AFC. Yet, this precursor appears to be suboptimal for further modification by downstream tailoring enzymes, resulting in the accumulation of this intermediate. In contrast, supplementation with palmitic or *cis*-vaccenic acid did not lead to ω-CFA accumulation, suggesting that these fatty acids may provide the optimal chain length and structural features required for efficient downstream processing in AFC biosynthesis.

Notably, *cis*-vaccenic acid (C18:1 ω7) is not only one of the most abundant fatty acid in *B. cenocepacia* ^26^ but also in most *Burkholderia sensu lato* strains, where it is considered a diagnostic marker.^25^ The variety of AFC derivatives observed in LB but not in PDB medium may reflect changes in the cellular fatty acid profile, thereby potentially providing a broader spectrum of precursors for AFC biosynthesis. Interestingly, previous work has linked the *afc* biosynthetic cluster to lipid metabolic pathways.^26^ Specifically, mutations in *afcE* and *afcF* were shown to alter the overall lipid composition, leading to an increased abundance of *cis*-vaccenic acid in the cell,^26^ possibly indicating an accumulation of this fatty acid when it is less efficiently utilized for AFC biosynthesis. Taken together, our findings are consistent with the idea that AFC biosynthesis directly incorporates fatty acids from primary metabolism, which appears to be influenced by growth conditions.

The greatest remaining mystery in the biosynthesis of both AFC-BC11 and bolagladins concerns the introduction of the citrate group into the fatty acyl chain. For bolagladin biosynthesis, it has been proposed that citryl–CoA may serve as the starter unit for assembly of the unusual fatty acid residue.^21,22^ In contrast, for AFC-BC11, it has been suggested that a citryl-CoA precursor couples to the terminal methyl group of a myristoyl intermediate.^20^ Based on detailed analysis of AFC derivatives and various feeding experiments, we suggest an alternative biosynthetic pathway for AFC-BC11. In this model, biosynthesis may be initiated by ω-oxidation of *cis*-vaccenic acid or its derivative, followed by a further oxidation step catalyzed by AfcU (Figure 6). Subsequent oxidation by AfcF, AfcS-mediated aldol addition, and methylation by AfcT could then complete the maturation of the terminus of the fatty acid moiety. This pathway would not require the biosynthesis of an unusual citryl-CoA starter unit, nor the proposed coupling of citryl-CoA to the terminal alkyl group of a myristoyl intermediate, nor an additional desaturase for the introduction of the double bond.

## Supplementary Material



## Notes

Detailed material and methods; supporting tables including compounds list, antifungal activity test, NMR chemical shift summary, bacterial strains and primers; and supporting figures including antifungal activity test, gene alignment, chromatogram and MS pattern, MS/MS fragment assignment, NMR spectra, chemical shift assignment, ozonolysis, possible biosynthetic pathway, quantification, and molecular networking (PDF)
