# Supplementary material for: Structural Elucidation and Revised Biosynthetic Pathway of the Membrane Vesicle–Associated Antifungal Compound AFC-BC11
Source: J Am Chem Soc. 2026 Sep 6;148(37):40012–21. doi: 10.1021/jacs.6c11512 (PMC13614206; doi:10.1021/jacs.6c11512)
Supplement: Supplementary file 1 [file ja6c11512_si_001.pdf]

Supplementary Information

Structural Elucidation and Revised Biosynthetic Pathway of the Membrane Vesicle–Associated Antifungal Compound AFC-BC11

Inês Tavares‡, Yi-Chi Chen‡, Kirsty Agnoli, Michael Berger, Felix Hartrampf, Simon Sieber*, Karl Gademann*, and Leo Eberl*

***Corresponding Authors**

Leo Eberl - *Department of Plant and Microbial Biology, University of Zurich, Zollikerstrasse 107, Zurich, CH 8008 Switzerland*; https://orcid.org/0000-0002-7241-0864;
Email: leberl@botinst.uzh.ch
Karl Gademann - *Department of Chemistry, University of Zurich, Winterthurerstrasse 190, Zurich, CH 8057, Switzerland*;
http://orcid.org/0000-0003-3053-0689; Email: karl.gademann@uzh.ch
Simon Sieber - *Department of Chemistry, University of Zurich, Winterthurerstrasse 190, Zurich, CH 8057, Switzerland*;
https://orcid.org/0000-0003-1763-2525; Email: simon.sieber@chem.uzh.ch

Authors

Inês Tavares - *Department of Plant and Microbial Biology, University of Zurich, Zollikerstrasse 107, Zurich, CH 8008 Switzerland;* https://orcid.org/0009-0005-3402-3491; Email: inesrosa.mendes@botinst.uzh.ch

Yi-Chi Chen - *Department of Plant and Microbial Biology, University of Zurich, Zollikerstrasse 107, Zurich, CH 8008 Switzerland;* https://orcid.org/0000-0003-3819-4085; Email: yi-chi.chen@botinst.uzh.ch

Kirsty Agnoli - *Department of Plant and Microbial Biology, University of Zurich, Zollikerstrasse 107, Zurich, CH 8008 Switzerland;* https://orcid.org/0000-0002-6317-1996; Email: k.agnoli@botinst.uzh.ch

Michael Berger - *Department of Plant and Microbial Biology, University of Zurich, Zollikerstrasse 107, Zurich, CH 8008 Switzerland;* Email: michael.berger@botinst.uzh.ch

Felix Hartrampf - *Department of Chemistry, University of Zurich, Winterthurerstrasse 190, Zurich, CH 8057, Switzerland*; https://orcid.org/0000-0002-6748-5271; Email: felix.hartrampf@chem.uzh.ch

‡Author Contributions

I.T. and Y.C. contributed equally.

Materials and Methods

Bacterial strains, plasmids and materials

Bacterial strains are listed in **Table S5** and plasmids in **Table S6**. Unless otherwise stated, strains were routinely cultured in Lysogeny Broth (Difco™ LB Broth, Lennox) or Potato Dextrose Broth (PDB, Difco™) at pH 5 at 37 ˚C with shaking. *B. thailandensis* was grown in LB without salt (LBNS) at 30 ˚C and *B. vietnamiensis* was grown in LBNS at 37 ˚C. Antifungal assays were done with Potato Dextrose Agar (PDA, Difco™) and Malt Extract Agar (MEA, Difco™).

Pseudomonas Isolation Agar (PIA, Difco™) and LB were used for selection of *Burkholderia* mutants or transconjugants. When necessary, antibiotics were used at the following concentrations: for *E. coli* 10 μg/mL tetracycline (Tc), 50 μg/mL trimethoprim (Tp), 25 μg/mL kanamycin (Km); for *B. cenocepacia* 100 μg/mL Tc, 150 μg/mL Tp, 50 μg/mL gentamycin (Gm).

Mutant construction

Unmarked deletion mutants were constructed as described by Flannagan and colleagues[1] using the SceI mutagenesis protocol. Briefly, two homologous regions upstream and downstream of the region to delete were amplified and cloned into pGPI-SceI. The pGPI constructs were conjugated into *B. cenocepacia* K56-2 by triparental mating using the pRK2013 helper plasmid, and the plasmid was integrated into the genome after a single crossover event. Strains with successful incorporation of pGPI were selected on PIA Tp. After this step, the plasmid pDAI-SceI was conjugated into the new strain, causing a double stranded break in its genome. Successful

recombination to repair the DNA damage and excision of pGPI resulted in resistant clones on LB Tc Gm. Primers used for mutant construction have been listed in **Table S7**.

afc cluster deletion

B. cenocepacia strain K56-2Δ*afcA* was constructed as follows: pSHAFT2-FRT-afcKOUP was constructed by amplifying a 1,334-bp fragment of *B. cenocepacia* pC3 using primers upXhoF and upBglIIR, digested with XhoI and BglII, and inserted into these sites within pSHAFT2-FRT. This plasmid was transferred into K56-2 by conjugation, and single-crossover insertion mutants were selected on PIA supplemented with chloramphenicol and verified by sequencing across the joins between pC3 and the pSHAFT2-FRT-afcUP. pEX18Tp-FRT-afcKODOWN was constructed by amplification of a 1,256-bp fragment of pC3 using primers afcKOdownPstF and afcKOdownBamR followed by digestion of this fragment with BamHI and PstI, and insertion into pEX18Tp-FRT digested with the same enzymes. This vector was transferred to K56-2 bearing the single-crossover insertion of pSHAFT2-FRT-afcKOUP, and insertion mutants were selected on PIA containing trimethoprim. Correct insertion was verified by PCR across the pC3-pEX18Tp-FRT-afcKODOWN joins. Excision of the *afc* cluster was achieved by the introduction of pBBR5::FLP, as described in [2]. The *shvR* gene was not deleted.

Construction of Δ*rep* and Δ*afcF* mutants

Since the *afc* cluster in K56-2 contains a duplication of an internal region, resulting in two copies of genes *afcE* and *afcS* and partial copies of genes *afcR* and *afcT*, this region was deleted in order

to obtain the K56-2 Δ*rep* strain without duplications and with a functional *afc* cluster. This strain was used to construct mutants Δ*afcR* and Δ*afcS.*

Gene *afcF* was deleted in the Δ*afcR* strain background, with *afcR* supplied *in trans* using pBBR-afcR.

MV isolation and quantification and nanoparticle tracking analysis

MV isolation and quantification was performed as previously described.[3–5] Briefly, cell cultures were pelleted at 22,000 × *g* for 10 min and the supernatant was filtered with a 0.45 μm PVDF membrane (Durapore Membrane Filter, Merck). The supernatant was ultracentrifuged at 150,000 × *g* at 4 ˚C for 1.5 h and the MV pellet was washed with Milli-Q H_2O and reharvested using the same conditions. MVs were resuspended in appropriate volumes of Milli-Q H_2O, depending on pellet size. Protein content was quantified with the Pierce™ BCA Protein Assay Kit. Lipid content was measured with the FM1-43 dye (Life Technologies, USA). Particle concentration and size distribution were determined by nanoparticle tracking analysis (NTA) using a Nanosight NS300 instrument (Malvern Panalytical). For NTA measurements, liquid cultures were centrifuged and filtered through a 0.45 μm PVDF membrane (Durapore Membrane Filter, Merck). Filtered supernatants were diluted with HyClone HyPure Water (GE) to a final concentration of 20 to 80 particles per frame. Samples were analyzed in light scattering mode for 1 min using maximum camera sensitivity and a detection threshold of 5. Each sample was measured 10 times (technical replicates).

Transmission Electron Microscopy

A 5 µl of diluted MVs was applied to glow-discharged, Formvar-coated 300-mesh copper grids (Electron Microscopy Sciences, USA). After 1 minute, excess liquid was removed using filter paper. Grids were negatively stained for with 1% uranyl acetate for 1 min and allowed to dry in a glass Petri dish. Samples were examined using an FEI Tecnai G2 Spirit TEM (FEI, Hillsboro, USA) at an accelerating voltage of 120 kV and equipped with a side-mounted digital Gatan Orius 1000 camera (4k ×2.6k pixels).

AFC-BC11 (1) quantification in the cell culture

B. cenocepacia K56-2 overnight cultures were used to inoculate 200 mL PDB subcultures at OD_{600} 0.01 in 500 mL baffled flasks, which were grown for 24 h at 37 ˚C. Cultures were harvested by centrifugation at 2200 × *g* for 40 min. The cell pellet was resuspended in 50 mL of a 0.9% NaCl solution, constituting the “Cells” sample, while the supernatant was retained as the “Supernatant with MVs” sample. The remaining 150 mL of the cultures were centrifuged at 22,000 × *g* for 10 min and the supernatant was filtered with a 0.45 µm PVDF membrane (Durapore Membrane Filter, Merck). The filtered supernatant was ultracentrifuged at 150,000 × *g* at 4 ˚C for 1.5 h. Following ultracentrifugation, 50 mL of the MV-free supernatant was collected as the “Supernatant without MVs” sample. The MV pellet was washed with Milli-Q H_2O, reharvested using the same

conditions and resuspended in 600 μL Milli-Q H_2O. 23 μL of MVs were diluted in 50 mL of a 0.9% NaCl solution, constituting the "MV" sample. AFC-BC11 from all samples was extracted by adding one volume of $CHCl_3$:MeOH=1:2. The mixtures were extracted vigorously and subsequently centrifuged (10 min at 21,300 × *g*) to separate the phases. The bottom chloroform layer and the top aqueous layer were removed, and the middle precipitate layer was extracted twice with DMF acidified with 1% formic acid with vortexing and sonication (Branson 2210 sonicator) for 10 min. The acidified DMF phase was then dried (Concentrator 5301, Eppendorf) and stored at –20 ˚C, before quantification of AFC-BC11 amount by UHPLC-HR-MS/MS. All samples for quantification were prepared with 3 biological replicates.

Bacterial culture and substrate supplementation for AFC-BC11 (1) production

All fatty acids, including myristic acid (Sigma), palmitic acid (Sigma), stearic acid (Sigma), *cis*-vaccenic acid (Sigma), myristic acid-d_{27} (Sigma), palmitic acid-d_{31} (Sigma), *cis*-vaccenic acid-d_{13} (Cayman Chemical) were dissolved in DMSO. Fatty acids mimics were supplemented with final concentration of 1 mM or 5 mM, respectively. K56-2 wildtype or mutant strains were inoculated in 2-5 mL PDB with OD 0.01 with or without supplements for 24 h in 37 ˚C. Cells were extracted by adding $CHCl_3$:MeOH=1:2 with the same amount as the culture volume. The mixture was extracted vigorously and subsequently centrifuged (10 min at 2,500 × *g*) to separate the phases. The bottom chloroform layer and the top aqueous layer were removed, and the middle precipitate layer was resuspended in DMF acidified with 1% formic acid with vortexing and sonication (Branson 2210 sonicator) for 10 min. After centrifugation (10 min at 21,300 × *g*), the supernatants

were then stored at 4 ˚C for further analysis. For comparison with the method of Zhong *et al,* 2025[6], both cultures were grown in PDB for 96 h at 37˚C. All samples for quantification and method comparison were prepared with 3 replicates.

Antifungal and antibacterial activity assays

Dual culture assays were performed against *R. solani* (obtained from the culture collection of the phytopathology group of the Institute of Plant Sciences, Federal Institute of Technology, Zurich, Switzerland). These were carried out as detailed by O'Grady and colleagues[7] on PDA or MEA plates. For antagonistic activity against *B. subtilis,* 30 to 50 μL of MVs was spotted on top of a lawn of *B. subtilis* on LB plates. The plates were incubated overnight at 37 ˚C. For antagonistic activity against *R. solani*, 30 to 50 μL of MVs were spotted on top of MEA plates. After the MV drops were dry, a suspension of disrupted mycelia from *R. solani* was sprayed on top, as described by Jenul *et. al*[8]. The plates were incubated for 2 days at room temperature in the dark.

AFC-BC11 (1) purification

B. cenocepacia K56-2 overnight cultures were used to inoculate PDB subcultures at OD_{600} 0.01, which were grown for 24 h at 37 ˚C. Cells were extracted by adding $CHCl_3$:MeOH=1:2 with the same amount as the culture volume. The mixture was extracted vigorously and subsequently centrifuged (10 min at 21,300 × *g*) to separate the phases. The bottom chloroform layer and the top aqueous layer were removed, and the middle precipitate layer was resuspended in DMF

acidified with 1% formic acid with vortexing and sonication (Branson 2210 sonicator) for 10 min. The suspension was centrifuged (10 min at 21,300 × *g*) and the supernatant was injected into an UltiMate™ 3000 UHPLC (ThermoScientific, USA) equipped with a reverse phase C18, 4.6 mm x 250 mm, 5.0 µm, 130 Å chromatography column (Waters Xselect CSH). The column was eluted with A: H_2O + 0.05% formic acid and B: MeCN + 0.05% formic acid with a flow rate of 1 mL/min at 28˚C and with the following gradient: 0-2 min, 37% B; 2-27 min, 37%-39% B; 27-27.1 min, 39%-100% B; 27.1-33 min, 100% B; 33-33.1 min, 100%-37% B; 33.1-35 min, 37% B. AFC-BC11 (**1**) elution was tracked by UV absorption at 320 nm and fluorescence (λ_{ext} = 330 nm, λ_{em} = 410 nm) by RS Diode Array Detector and RS Fluorescence Detector, respectively. Fractions were collected and rotary-evaporated in a 45 ˚C water bath in the dark. Purified samples were stored at -20 ˚C prior to further NMR and MS analysis. For *B. puraquae* AFC-BC11 (**1**) purification, the whole procedure, including bacterial culture and extraction, was performed via the method previously described.[6] AFC-BC11 (**1**) peak elutes at 19 min. The optical rotation of AFC-BC11 (**1**) was measured on a Jasco P-2000 polarimeter (Portmann Instruments AG) using a 1 cm cell in DMSO at 23 ˚C ($[\alpha]_D$ = +0.77 (*c* 0.05, DMSO)).

UHPLC and UHPLC-HR-MS/MS analysis

The analysis of crude and purified AFC-BC11 (**1**) was performed in an UltiMate™ 3000 UHPLC (ThermoScientific, USA) equipped with a reverse phase C18, 4.6 mm x 250 mm, 5.0 µm, 130 Å chromatography column (Waters Xselect CSH). The column was eluted with A: H_2O + 0.05%

formic acid and B : MeCN + 0.05% formic acid with a flow rate of 1 mL/min at 45˚C and with the following gradient: 0-1 min, 4%-44% B; 1-9 min, 44% B; 9-10 min, 44%-100% B; 10-14 min, 100% B; 14-14.1 min, 100%-4% B; 14.1-16 min, 4% B. AFC-BC11 (**1**) elution was tracked by UV absorption at 320 nm and fluorescence (λ_{ext} = 330 nm, λ_{em} = 410 nm) by RS Diode Array Detector and RS Fluorescence Detector, respectively. AFC-BC11 (**1**) elutes at 8.75 min.

UHPLC-HR-MS/MS analysis was performed by timsTOF Pro hybrid quadrupole-time-of-flight (QTOF) mass spectrometer equipped with trapped ion mobility spectrometry (TIMS) produced by Bruker (Bremen, Germany) or Q Exactive hybrid quadrupole-Orbitrap mass spectrometer by Thermo Fisher Scientific (Waltham, MA, USA) equipped with a heated ESI source at position C and a voltage of ±3.5 kV. A Vanquish UHPLC system and was used with ACQUITY Premier CSH C18 Column 1.7 µm, 2.1 x 100 mm reverse-phase column (Waters). The column was eluted with A: H_2O + 0.1% formic acid and B: MeCN + 0.1% formic acid with a flow rate of 0.3 mL/min at 28 ˚C and with the following gradient: 0-2 min, 4% B; 2-8 min, 4%-100% B; 8-12 min, 100% B; 12-12.1 min, 100%-4% B; 12.1-14 min, 4% B. MS data was acquired in positive ESI ionization mode. For timsTOF, the data was recorded without ion mobility and the scan range was set to 80 to 1200 *m/z* at a 12 Hz base acquisition rate. Fragment spectra were acquired using the data-dependent acquisition mode (AutoMS/MS) employing 30 and 70 eV fragmentation energies. Mass calibration was performed using the Agilent low concentration tuning mix (13 compounds in acetonitrile, part number G1969-85020) prior to analysis. For additional mass accuracy, a calibration segment was programmed from 0.05 to 0.15 min at every UHPLC run with the help of

a 6-port-valve with a 20 µL loop which contained a solution of 10 mM sodium formate. The Q Exactive instrument was operated in high-resolution full-scan mode. For full MS, a resolution of 70,000 (200 *m/z* at full width at half maximum) was selected with a maximum IT of 200 ms and an AGC target of 3e6. Scan range was set at 80-800 *m/z*. For PRM, ion 452.2643 was selected, and a resolution of 35,000 (200 *m/z* at full width at half maximum) was applied with a maximum IT of 110 ms and an AGC target of 5e5. The instrument was calibrated at a mass accuracy ≤ 2 ppm with a PierceTM LTQ Velos ESI Positive Ion Calibration Solution (Thermo). External calibration was performed before each measurement run.

Data quantification was performed by TASQ (Bruker, v2022b). AFC derivatives ions with 1 and 2 positive charges were selected and all the automatic integrations were revised manually. The ratio of ion abundance was made from an average of 3 independent replicates. Note that since the major AFC-BC11-660 (**9**) and AFC-BC11-634 (**10**) ions have 2 positive charges, the ratio may not reflect the real quantity of these molecules. AFC-BC11 (**1**) was detected at 6.45 min with 734.3965 (calc. for $C_{36}H_{56}N_5O_{11}^+$ 734.3971, $\Delta m = 0.82$ ppm). AFC-707 (**2**) was detected at 6.25 min with 708.3805 (calc. for $C_{34}H_{54}N_5O_{11}^+$ 708.3814, $\Delta m = 1.27$ ppm). AFC-BC11-645 (**3**) was detected at 6.75 min with 646.3798 (calc. for $C_{33}H_{52}N_5O_8^+$ 646.3810, $\Delta m = 1.86$ ppm). AFC-BC11-619 (**4**) was detected at 6.55 min with 620.3643 (calc. for $C_{31}H_{50}N_5O_8^+$ 620.3654, $\Delta m = 1.77$ ppm). AFC-BC11-661 (**5**) was detected at 6.35 min with 662.3751 (calc. for $C_{33}H_{52}N_5O_9^+$ 662.3760, $\Delta m = 1.36$ ppm). AFC-BC11-635 (**6**) was detected at 6.20 min with 636.3592 (calc. for $C_{31}H_{50}N_5O_9^+$ 636.3603, $\Delta m = 1.73$ ppm). AFC-BC11-659 (**7**) was detected at 6.95 min with 660.3591 (calc. for $C_{33}H_{50}N_5O_9^+$ 660.3603, $\Delta m = 1.82$ ppm). AFC-BC11-633

(**8**) was detected at 6.80 min with 634.3438 (calc. for $C_{31}H_{48}N_5O_9^+$ 634.3447, $\Delta m = 1.42$ ppm). AFC-BC11-660 (**9**) was detected at 5.55 min with 661.391 (calc. for $C_{33}H_{53}N_6O_8^+$ 661.3919, $\Delta m = 1.36$ ppm) and 331.1999 (calc. for $C_{33}H_{54}N_6O_8^{2+}$ 331.1996, $\Delta m = -0.88$ ppm). AFC-BC11-634 (**10**) was detected at 5.40 min with 635.3753 (calc. for $C_{31}H_{51}N_6O_8^+$ 635.3763, $\Delta m = 1.57$ ppm) and 318.1924 (calc. for $C_{31}H_{52}N_6O_8^{2+}$ 318.19178, $\Delta m = -1.95$ ppm).

Molecular Networking analysis was further processed on Δ*afcF*, Δ*afcS* and wildtype strains with the software MZmine (version 4.7.8) using MZwizard[9–11]. Intensity in all the MS spectra are shown in either relative abundance (%) or arbitrary units (a.u.).

NMR analysis

^1H NMR spectra were recorded in DMSO-d_6 on the instruments Bruker AV-600 (600 MHz) or on the instrument Bruker AVANCE NEO (1.2 GHz); chemical shifts δ in ppm relative to solvent signals (δ = 2.50 ppm for DMSO-d_6), coupling constants J are given in Hz. ^{13}C NMR spectra were recorded in DMSO-d_6 on the instruments Bruker AV-600 (126 MHz); chemical shift in ppm relative to solvent signals (δ = 39.53 ppm for DMSO-d_6). The NMR spectra were recorded at 313K.

Ozonolysis of AFC-BC11 (1)

The procedure followed reported protocols with modifications.[12–14] AFC-BC11 (**1**, 100 μL, DMF) was transferred to a round-bottom flask (5 mL), and the solution was diluted with MeOH (900 μL), cooled to -78 ˚C, and treated with ozone for 2 min. Nitrogen was bubbled into the blue

solution for 1 min before H_2O_2 (50 μL, 30%) was added, and the solution was stirred at RT for 1 h. The solvent was evaporated, and the solids were dissolved in MeOH/H_2O (100 μL, 20%), centrifuged (5 min at 20,238 × *g*) and analyzed by UHPLC-HR-MS with a UHPLC system (Vanquish Horizon, ThermoFischer) equipped with a Diode Array Detector, Split Sampler HT, Binary Pump H, Column Compartment H connected to an HRMS (Exploris 240, Thermo Scientific, USA) in negative and positive modes using a HILIC column (BEH amide from Waters, 2.1 x 100 mm, 1.7 μm).

For the analysis, 2 μL was injected, the flow rate was 400 μL/min, the column oven was set to 40 ˚C, and the solvent was composed of A: a 5 mM aq. NH_4HCO_3 soln. and B: MeCN. The column was equilibrated for 0.5 min with 95% B and, after injection, the isocratic gradient was kept for 0.5 min at 95% B. The running gradient was decreased to 5% B over 3 min and stayed at 5% B for 2 min. The gradient was increased to the initial conditions at 95% B over 0.1 min and kept for 1 min at those conditions. The ion source parameters of the MS in positive and negative mode were set as follows: spray voltage 2.5 kV for negative mode and 3.5 kV for positive mode; ion transfer tube temperature at 325 ˚C; sheath gas 50 L/min; aux gas 10 L/min; sweep gas 1 L/min; and vaporizer temperature 340 ˚C. The EASY-ICTM internal calibration system was used at the beginning of each run. A full scan with a resolution of 60,000, a scan range of 70-900 *m/z*, and RF lens of 70% were used in positive mode for one run and negative mode for another run and a $ddMS^2$ was set up with a scan width of 0.4 *m/z*, at a resolution of 30,000, and with an HCD collision energy at 15, 30, and 50%) and using a previously defined list of potential degradation products ($C_{10}H_{15}O_7^-$ $[M – H]^-$, $C_{11}H_{20}NO_3^-$ $[M – H]^-$, $C_6H_6NO_7^-$ $[M – H]^-$, and $C_{11}H_{22}NO_3^+$ $[M + H]^+$).

Supplementary tables

Table S1. Summary of chemical shifts and correlations for AFC-BC11 (**1**). The chemical shifts of the carbon atoms were determined by a combination of ^{1}H–^{13}C HMBC and ^{1}H–^{13}C HSQC-TOCSY analyses.

Moiety	Pos.	δ_H in ppm (J_{HH} in Hz)	δ_C, type	^{1}H-^{13}C HMBC	^{1}H-^{1}H NOESY	^{1}H-^{1}H TOCSY (100ms)	^{1}H-^{13}C HSQC-TOCSY (60ms)	^{1}H-^{13}C HSQC-TOCSY (90ms)
DHLys	1		nd					
	2	4.30 (m)	52.3, CH		DHLys(3a,3b)	DHLys(3a,3b,4,5,NH)	DHLys(3)	
	3a	2.51 (m)	29.5, CH_2			DHLys(2,4,5,NH)		
	3b	2.51 (m)				DHLys(2,4,5,NH)		
	4	5.72 (m)	nd	DHLys(6)	DHLys(5)	DHLys(2,3a,3b,5,6a,6b)	DHLys(3,6)	
	5	5.54 (dt, J=11.0,7.4)	123.4, CH	DHLys(3)		DHLys(2,3a,3b,4,6a,6b)	DHLys(6)	DHLys(6)
	6a	3.46 (m)	35.2, CH_2	DHLys(4,5)		DHLys(3a,3b,4,5)	DHLys(5)	
	6b	3.34 (m)				DHLys(3a,3b,4,5)		
	NH	7.91 (br)			DBA^{1}(NH)	DHLys(2,3a,3b)		
	NH_2							
DBA^{1}	1		165.1, C					
	2	5.77 (d, J=13.9)	104.2, CH	DBA^{1}(1, 3)	DHLys(NH), DBA^{1}(NH), DBA^{2}(2)	DBA^{1}(3,NH)	DBA^{1}(3)	DBA^{1}(3)
	3	7.72 (dd, J=13.9, 11.1)	133.1, CH	DBA^{1}(1), DBA^{2}(1)	DBA^{1}(2,NH)	DBA^{1}(2,NH)	DBA^{1}(2)	DBA^{1}(2)
	NH	10.56 (d, J=11.1)		DBA^{2}(1)	DBA^{1}(2,3), DBA^{2}(2,3)	DBA^{1}(2,3)	DBA^{1}(2,3)	DBA^{1}(2,3)
DBA^{2}	1		165.7, C					
	2	5.29 (m)	98.0, CH	DBA^{2}(1)	DBA^{1}(2,NH), DBA^{2}(3)	DBA^{2}(3,NH)		DBA^{2}(3)
	3	7.46 (dd, J=11.2,8.9)	136.0, CH	DBA^{2}(1)	DBA^{1}(NH), DBA^{2}(2)	DBA^{2}(2,NH)		DBA^{2}(2)
	NH	10.97 (d, J=11.2)		DBA^{3}(1)	DBA^{1}(3), DBA^{2}(2,3), DBA^{3}(2)	DBA^{2}(2,3)	DBA^{2}(2)	DBA^{2}(2,3)
DBA^{3}	1		165.3, C					
	2	5.49, (d, J=8.8)	97.0, CH	DBA^{3}(1)	DBA^{2}(NH), DBA^{3}(3)	DBA^{3}(3,NH)		DBA^{3}(3)
	3	7.44 (dd, J=11.3,8.8)	137.6, CH	DBA^{3}(1)	DBA^{2}(NH), DBA^{3}(2), FA(2)	DBA^{3}(2,NH)		DBA^{3}(2)
	NH	10.89 (d, J=11.3)			DBA^{3}(3), FA(2)	DBA^{3}(2,3)	DBA^{3}(2)	DBA^{3}(3)
FA	1		170.9, C					
	2	2.41 (t, J=7.4)	35.2, CH_2	FA (1,3,4)	DBA^{3}(NH,3), FA(3,4)	FA (3,4)	FA (3,4)	FA (3,4)
	3	1.55 (m)	24.0, CH_2	FA (1,4)	DBA^{3}(NH), FA(2,4)	FA (2)	FA (2,4)	FA (2,4)
	4	1.27 (m)	28.0, CH_2					
	5	nd	nd					
	6	nd	nd					
	7	nd	nd					
	8	nd	28.3, CH_2					
	9	1.29	28.8, CH_2					
	10	1.97 (q, J=6.9)	26.3, CH	FA(8,9,11/12)	FA(11/12)	FA (11/12)	FA (8,9,11/12)	FA (8,9,11/12)
	11	5.31 (dt, J = 11.8, 6.9)	129.2, CH_2	FA(10,13)	FA(10,13)	FA (10,13,16)	FA (9,10,13,14,15)	FA (8,9,10,13,14,15,16)
	12	5.31 (dt, J =11.8, 6.7)	129.2, CH_2	FA(10,13)	FA(10,13)	FA (10,13,16)	FA (9,10,13,14,15)	FA (8,9,10,13,14,15,16)
	13	1.95 (q, J=6.7)	26.4, CH	FA(11/12,14,15)	FA(11/12,16)	FA (11/12,16)	FA (11/12,14,15,16)	FA (11/12,14,15,16)
	14	1.23(m)	29.4, CH_2					
	15	1.31(m)<br>1.22(m)	22.3, CH_2					
	16	1.57-1.58 (m)	35.7, CH_2	FA (15,17)	FA (13,19a,19b,21)	FA (11/12,13)	FA (13,14,15)	FA (13,14,15)
	17		79.1, C					
	18/20		174.0/172.1, C					
	19a	2.75 (d, J=14.5)	42.8, CH_2	FA (17,18/20,21)	FA (15,16,19b,21)	FA (19b)		
	19b	2.23 (d, J=14.5)			FA (15,16,19a,21)	FA (19a)		
	21	3.09 (s)	50.7, CH_3	FA (17)	FA (15,16,19a,19b)			

Data highlighted in red were determined from measurements performed on a 1.2 GHz spectrometer.

Table S2. Summary of chemical shifts and correlations for AFC-BC11 (**1**) obtained from LB medium. The chemical shifts of the carbon atoms were determined by a combination of $^1H–^{13}C$ HMBC and $^1H–^{13}C$ HSQC-TOCSY analyses.

Moiety	Pos.	δ_H in ppm (J_{HH} in Hz)	δ_C, *type*	^1H-^{13}C HMBC	^1H-^1H NOESY	^1H-^1H TOCSY	^1H-^{13}C HSQC-TOCSY
DHLys	1		172.6, C				
	2	4.39 (td, *J*=8.2, 5.5)	51.0, CH	DHLys(1,3,4), DBA[1](1)	DHLys(3a,3b,NH)	DHLys(3a,3b,4,5,NH)	DHLys(3)
	3a	2.46 (m)	29.0, CH_2	DHLys(1,2,4,5)		DHLys(2,4,5,6,NH)	DHLys(2,4)
	3b	2.53 (m)		DHLys(1,2,4,5)		DHLys(2,4,5,6,NH)	DHLys(2,4)
	4	5.68 (m)	130.7, CH	DHLys(6)	DHLys(5)	DHLys(2,3a,3b,5,6,NH)	DHLys(3,5,6)
	5	5.52 (m)	123.7, CH	DHLys(3)	DHLys(4,6)	DHLys(2,3a,3b,4,6,NH)	DHLys(4,6)
	6	3.47 (m)	35.4, CH_2	DHLys(4,5)		DHLys(3a,3b,4,5)	DHLys(4,5)
	NH	8.12 (d, *J*=8.2)		DBA[1](1)	DHLys(2,3a,3b,); DBA[1](2,NH)	DHLys(2,3a,3b,4,5,6)	DHLys(2)
	NH_2						
DBA[1]	1		165.4, C				
	2	5.75 (d, *J*=14.0)	103.6, CH	DBA[1](1, 3)	DHLys(NH), DBA[1](3,NH), DBA[2](2)	DBA[1](3,NH)	DBA[1](3)
	3	7.74 (dd, *J*=14.0, 11.1)	133.3, CH	DBA[1](1)	DHLys(NH), DBA[1](2,NH)	DBA[1](2,NH)	DBA[1](2)
	NH	10.59 (d, *J*=11.1)		DBA[2](1)	DHLys(NH), DBA[1](2,3), DBA[2](2,3)	DBA[1](2,3)	DBA[1](2,3)
DBA[2]	1		165.6, C				
	2	5.29 (m)	97.8, CH	DBA[2](1,3)	DBA[1](2,NH), DBA[2](3)	DBA[2](3,NH)	DBA[2](3)
	3	7.47 (dd, *J*=11.2, 8.9)	136.0, CH	DBA[2](1,2)	DBA[1](NH), DBA[2](2,NH)	DBA[2](2,NH)	DBA[2](2)
	NH	10.97 (d, *J*=11.2)		DBA[3](1)	DBA[2](3), DBA[3](2)	DBA[2](2,3)	DBA[2](3)
DBA[3]	1		165.3, C				
	2	5.49 (d, *J*=8.8)	96.8, CH	DBA[3](1)	DBA[2](NH), DBA[3](3)	DBA[3](3,NH)	DBA[3](3)
	3	7.44 (dd, *J*=11.0, 8.8)	137.7, CH	DBA[3](1)	DBA[3](2,NH), FA(2)	DBA[3](2,NH)	DBA[3](2)
	NH	10.87 (d, *J*=11.0)			DBA[3](3), FA(2)	DBA[3](2,3)	DBA[3](3)
FA	1		170.8, C				
	2	2.41 (t, *J*=8.4)	35.1, CH_2	FA(1,3,4)	DBA[3](NH,3), FA(3)	FA(3,4)	FA(3,4)
	3	1.56 (m)	23.9, CH_2	FA(1,2,4,5)	DBA[3](NH), FA(2,4)	FA(2,4)	FA(2,4)
	4	1.27 (m)	27.9, CH_2				
	5						
	6						
	7						
	8						
	9	1.26 (m)	28.3, CH_2				
	10/13	1.98 (m)	26.1, CH_2	FA(9,11/12,14)	FA(9,11/12,14,16)	FA(9,11/12,14,16)	FA(9,11/12,14,15)
	11//12	5.32 (m)	129.1, CH	FA(10/13)	FA(10/13)	FA(9,10/13,14,16)	FA(9,10/13,14)
	14	1.26 (m)	29.1, CH_2				
	15	1.22 (m)	22.1, CH_2				
	16	1.69 (m)	33.8, CH_2		FA(21)	FA(10/13, 11/12)	FA(14,15)
	17	-	79.7, C				
	18						
	19	2.67 (m)	39.3, CH_2	FA(17)	FA(21)		
	20						
	21	3.15 (s)	50.5, CH_3	FA(17)	FA(15,16)		

Table S3. Expected structures of AFCs after supplementation with unlabeled or deuterium-labeled myristic acid, palmitic acid, or *cis*-vaccenic acid, along with the corresponding $[M+H]^+$ ion structure and molecular formulas. Possible deuterium-labeled positions are highlighted in green.

**myristic acids**	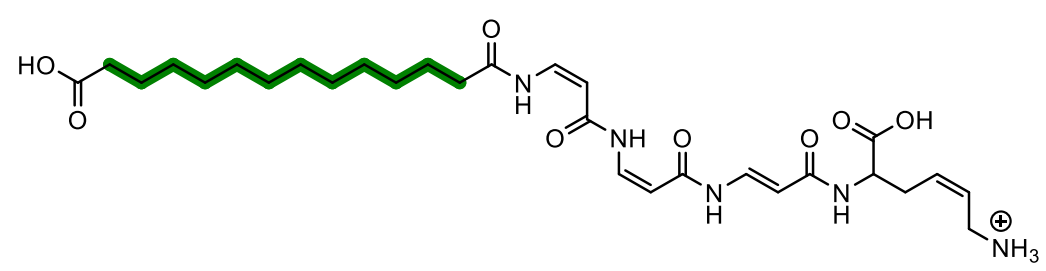	$C_{29}H_{46}N_5O_8^+$<br>(**11**)	$C_{29}H_{22}\mathbf{D_{24}}N_5O_8^+$<br>(**12**)
	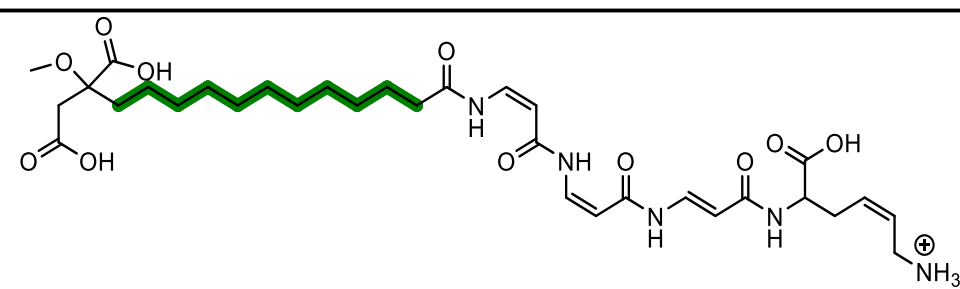	$C_{32}H_{50}N_5O_{11}^+$<br>(**13**)	$C_{32}H_{28}\mathbf{D_{22}}N_5O_{11}^+$<br>(**14**)
	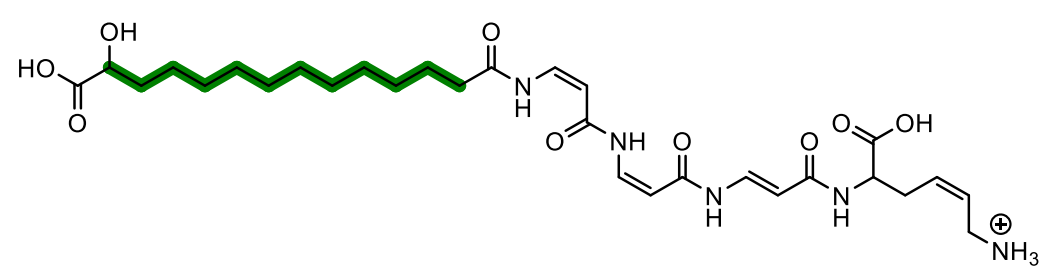	$C_{29}H_{46}N_5O_9^+$<br>(**15**)	$C_{29}H_{23}\mathbf{D_{23}}N_5O_9^+$<br>(**16**)
**palmitic acids**	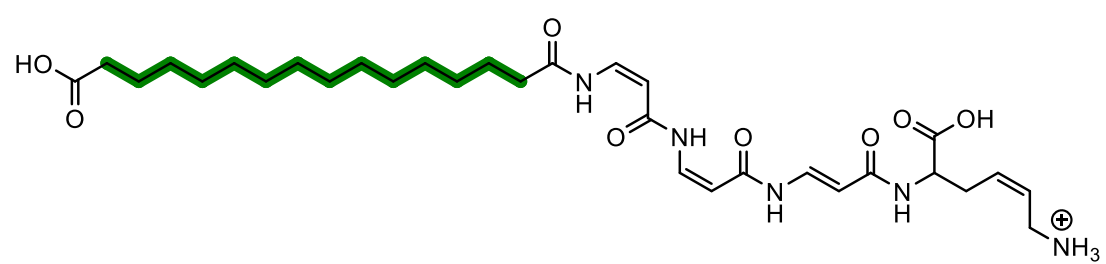	$C_{31}H_{50}N_5O_8^+$<br>**AFC-BC11-619**<br>(**4**)	$C_{31}H_{22}\mathbf{D_{28}}N_5O_8^+$<br>**AFC-BC11-619-$d_{28}$**<br>(**18**)
	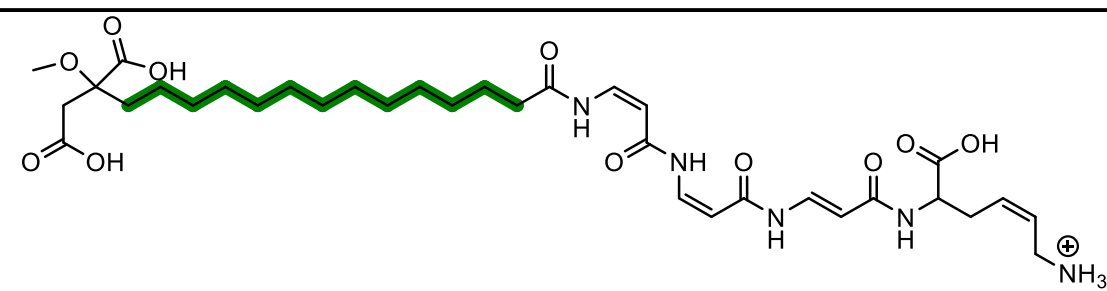	$C_{34}H_{54}N_5O_{11}^+$<br>**AFC-BC11-707**<br>(**2**)	$C_{34}H_{28}\mathbf{D_{26}}N_5O_{11}^+$<br>**AFC-BC11-707-$d_{26}$**<br>(**17**)
***cis*-vaccenic acids**	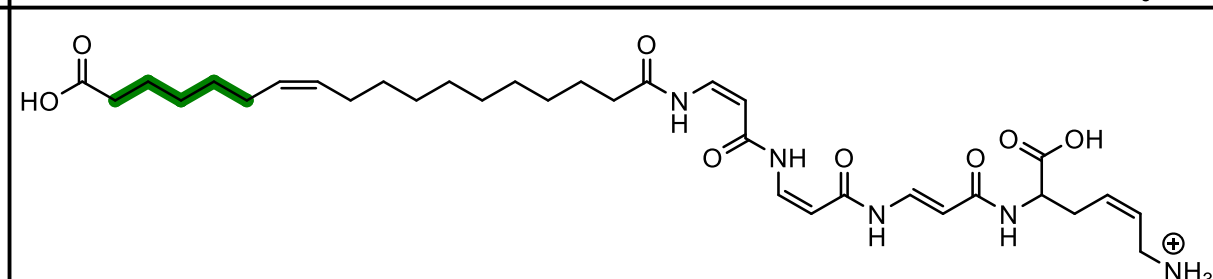	$C_{33}H_{52}N_5O_8^+$<br>**AFC-BC11-645**<br>(**3**)	$C_{33}H_{42}\mathbf{D_{10}}N_5O_8^+$<br>**AFC-BC11-645-$d_{10}$**<br>(**21**)
	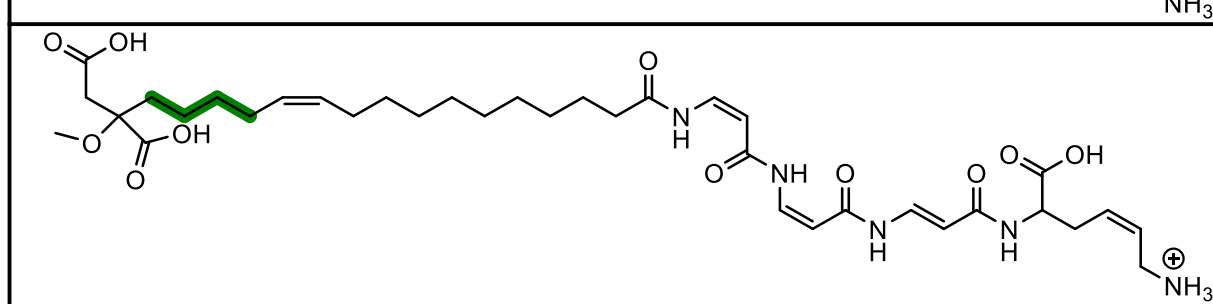	$C_{36}H_{56}N_5O_{11}^+$<br>**AFC-BC11**<br>(**1**)	$C_{36}H_{48}\mathbf{D_8}N_5O_{11}^+$<br>**AFC-BC11-$d_8$**<br>(**19**)
	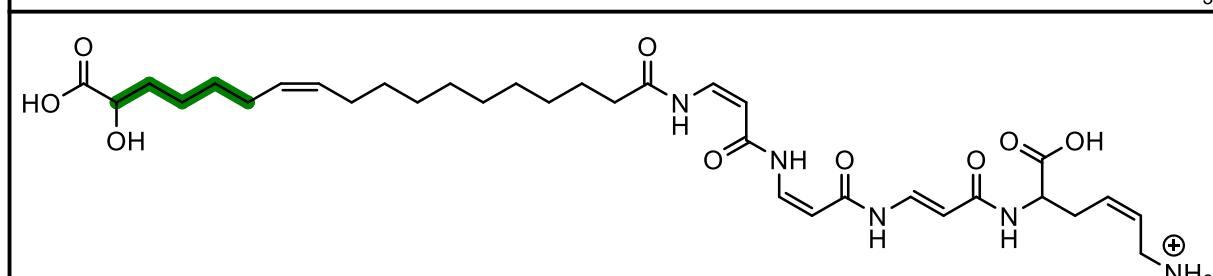	$C_{33}H_{52}N_5O_9^+$<br>**AFC-BC11-661**<br>(**5**)	$C_{33}H_{43}\mathbf{D_9}N_5O_9^+$<br>**AFC-BC11-661-$d_9$**<br>(**22**)
	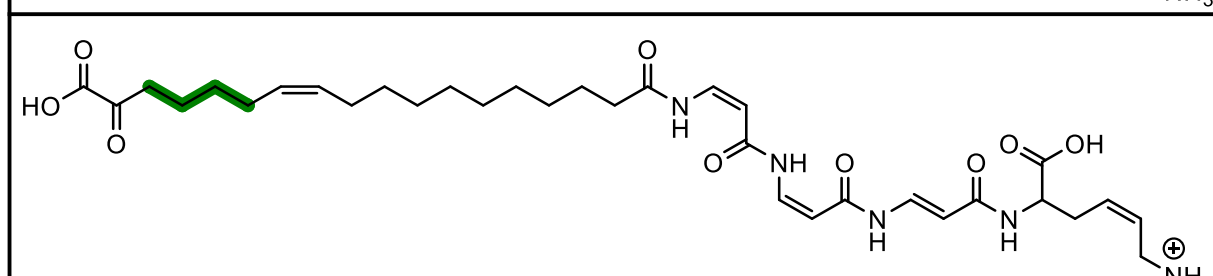	$C_{33}H_{50}N_5O_9^+$<br>**AFC-BC11-659**<br>(**7**)	$C_{33}H_{42}\mathbf{D_8}N_5O_9^+$<br>**AFC-BC11-659-$d_8$**<br>(**23**)
	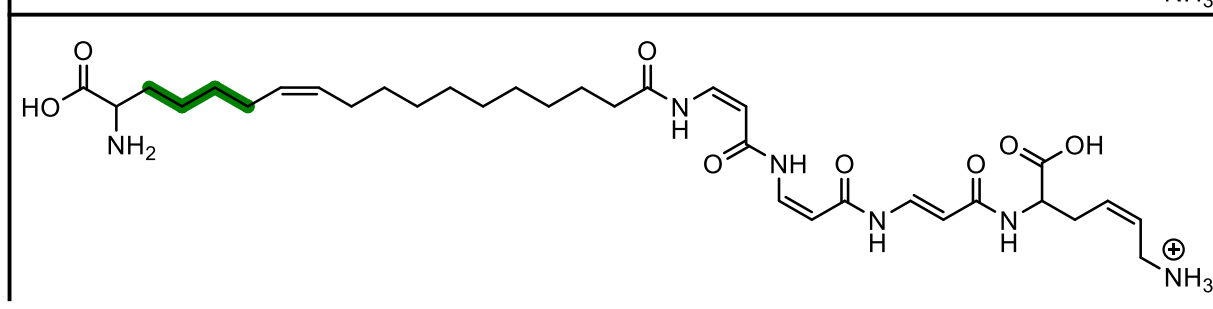	$C_{33}H_{53}N_6O_8^+$<br>**AFC-BC11-660**<br>(**9**)	$C_{33}H_{45}\mathbf{D_8}N_6O_8^+$<br>**AFC-BC11-660-$d_8$**<br>(**24**)

Table S4. Colony morphology and antifungal activity against *R. solani* of the *afc* mutants used in this study.

Strain	Colony morphology	Antifungal Activity		Reference
**AFC-producing**				
WT	Rough	Yes	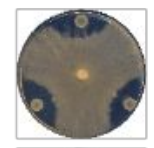	Subramoni *et al*., 2013
Δ *afcB*	Rough	Yes	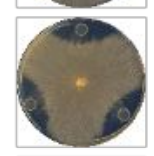	This study
Δ *afcG*	Rough	Yes	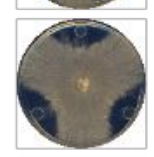	This study
Δ *afcH*	Rough	Yes	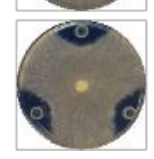	This study
Δ *afcI*	Rough	Yes	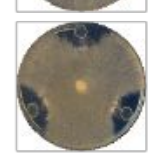	This study
Δ *afcS*	Rough	No	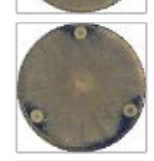	This study
Δ *afcF*	Rough	No	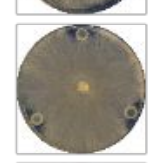	This study
Δ *afcU*	Rough	No	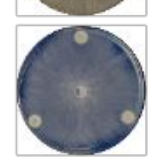	This study
**AFC-non-producing**				
Δ *afc*	Shiny	No	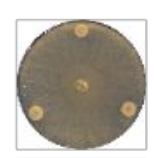	This study
Δ *afcA*	Shiny	No	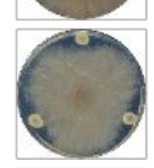	This study
Δ *afcJ*	Shiny	No	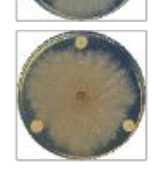	This study
Δ *afcK*	Shiny	No	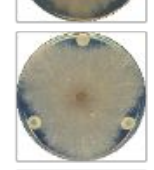	This study
Δ *afcL*	Shiny	No	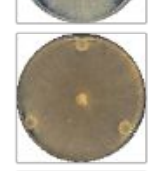	This study
Δ *afcM*	Shiny	No	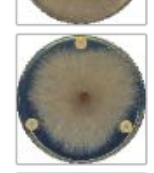	This study
Δ *afcO*	Shiny	No	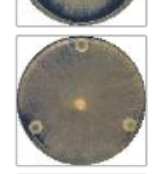	This study
Δ *afcP*	Shiny	No	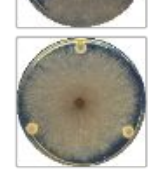	This study
Δ *afcQ*	Shiny	No	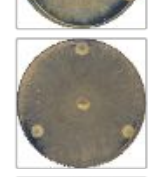	This study
Δ *afcR*	Shiny	No	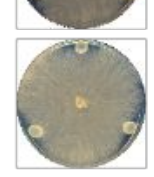	This study
Δ *afcV*	Shiny	No	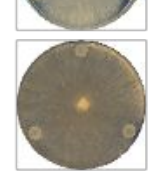	This study

Table S5. Bacterial strains

Strain	Genotype or Description	Reference
***E. coli* strains**		
SY327λpir	wild type; *araD*, Δ(*lac pro*) *argE*(Am) *recA56* Rif^R *nalA λpir*	15
DH5α	F- Φ80d*lacZ*Δ*M15* Δ(*lacZYA-argF*)U169 *recA1 endA1 hsdR17(rK- mK+) supE44 thi-1 relA1 gyrA96*	16
MC1061	F⁻ *araD139* Δ*(ara, leu)7697* Δ*(lacIPOZYA)X74 galK16 galE15 rpsL150 mcrA0 mcrB1 hsdR2 relA::IS2 spoT e14*⁻ λ⁻ (Sm^R)	17
***Burkholderia* strains**		
Burkholderia lata 383	Isolated from forest soil in Trinidad and Tobago	18
Burkholderia vietnamiensis LMG10929	Isolated from rice rhizosphere soil	19
Burkholderia vietnamiensis ABIP434	Rice rhizosphere soil isolate	20
Burkholderia thailandensis E264	isolated from soil of a rice field	21
Burkholderia puraquae DSM103137	isolated from haemodialysis water	22
Burkholderia cenocepacia H111	CF isolate from Germany, R-6282	23
***Burkholderia cenocepacia* K56-2 strains**		
K56-2	CF sputum isolate (Canada); ET12 lineage	24
Δ*afc*	K56-2 deletion mutant of the *afc* cluster	This study
Δ*afcA*	K56-2 deletion mutant of *afcA*	This study
Δ*afcB*	K56-2 deletion mutant of *afcB*	This study
Δ*afcG*	K56-2 deletion mutant of *afcG*	This study
Δ*afcH*	K56-2 deletion mutant of *afcH*	This study
Δ*afcI*	K56-2 deletion mutant of *afcI*	This study
Δ*afcJ*	K56-2 deletion mutant of *afcJ*	This study
Δ*afcK*	K56-2 deletion mutant of *afcK*	This study
Δ*afcL*	K56-2 deletion mutant of *afcL*	This study
Δ*afcM*	K56-2 deletion mutant of *afcM*	This study
Δ*afcO*	K56-2 deletion mutant of *afcO*	This study
Δ*afcP*	K56-2 deletion mutant of *afcP*	This study
Δ*afcQ*	K56-2 deletion mutant of *afcQ*	This study
Δ*rep*	K56-2 deletion mutant of the duplicated *afcREST* genes	This study
Δ*rep*Δ*afcR*	K56-2 Δ*rep* deletion mutant of *afcR*	This study
Δ*afcS*	K56-2 Δ*rep* derivative with *afcS* deleted	This study
Δ*afcU*	K56-2 deletion mutant of *afcU*	This study
Δ*afcV*	K56-2 deletion mutant of *afcV*	This study
Δ*afcF*	K56-2 Δ*rep*Δ*afcR* derivative with *afcF* deleted bearing pBBR-afcR	This study
**Other strains**		
Bacillus subtilis 168	With plasmid pHY300-zsGreen-term to confer resistance to Tc	3

Table S6. Plasmids

Plasmids	Description	Reference
pRK2013	Helper plasmid; RK2 derivative, mob+ tra+ ori ColE1; Km^R	25
pGPI-SceI	Suicide plasmid with oriR6K, mob+, I-SceI restriction site; Tp^R	Elisabeth Steiner, Laboratory collection
pDAI-SceI	pDA17 plasmid carrying the I-SceI nuclease gene; Tc^R	1
pBBR1MCS-3	Mobilisable BHR cloning vector; IncP and ColE1 compatible; Tc^R)	26
pBBR-afcR	pBBR1MCS-3 containing the *afcR* gene	This study

Table S7. Primers used in this study.

Name	Sequence (5´→3´)[a]	Construction
upXhoF	gcgcctcgagTTCTGATCTTCCTCGTGCTC	Δ*afc*
upBglIIR	gcgcagatctGCAACGCAATCAGAACACC	Δ*afc*
afcKOdownPstF	gcgcctgcagATTCATCTTGACGGTCGTCG	Δ*afc*
afcKOdownBamR	gcgcggatccTTGATCGTACTGGCTGAAGT	Δ*afc*
afcA up XbaI	cgcgTCTAGACTGGTACGCATGAAAC	Δ*afcA*, XbaI site
afcA SOE R	TCATGCGAACGCTCCCTGAGGATTCTCCCCTTG	Δ*afcA*
afcA SOE F	CAAGGGGAGAATCCTCAGGGAGCGTTCGCATGA	Δ*afcA*
afcA down EcoRI	gcgcGAATTCAATCAGAAAGGCGGTGC	Δ*afcA*, EcoRI site
afcB_up_XbaI_F	gcgcTCTAGACTACCTGAACCCCGACGA	Δ*afcB*, XbaI site
afcB_SOE_R	AACGTCGACCAGTTCATCATGCGAACGCTCCC	Δ*afcB*
afcB_SOE_F	GGGAGCGTTCGCATGATGAACTGGTCGACGTT	Δ*afcB*
afcB_down_SmaI_R	gcgcCCCGGGACCGTGTGATAGTCGAAGAA	Δ*afcB*, SmaI site
220_up_XbaI_F	gcgcTCTAGATGTTCCAGCAATTCAACCTG	Δ*afcG*, XbaI site
220_SOE_R	GACGAATGCGATTCATTACGCCACGCAAACGT	Δ*afcG*
220_SOE_F	ACGTTTGCGTGGCGTAATGAATCGCATTCGTC	Δ*afcG*
220_down_SmaI_R	gcgcCCCGGGAGCTTGATCGTCGGGTAGA	Δ*afcG*, SmaI site
219_up_XbaI_F	gcgcTCTAGAGTATCGCTCTATCACCAGGT	Δ*afcH*, XbaI site
219_SOE_R	CGTACTCACTGGCCATCATGGGGCGAGTCCTT	Δ*afcH*
219_SOE_F	AAGGACTCGCCCCATGATGGCCAGTGAGTACG	Δ*afcH*
219_down_SmaI_R	gcgcCCCGGGCCCATTTCGCTTCGACGA	Δ*afcH*, SmaI site
218_up_XbaI_F	gcgcTCTAGAGAGAACGACGGCCAGAAAT	Δ*afcI*, XbaI site
218_SOE_R	CATGGCAGGGTTCCTGTCACTGGCCATCGGCG	Δ*afcI*
218_SOE_F	CGCCGATGGCCAGTGACAGGAACCCTGCCATG	Δ*afcI*
218_down_SmaI_R	gcgcCCCGGGGCCGTCGCTCATCAGGAA	Δ*afcI,* SmaI site
217 up XbaI	gcgcTCTAGACCGCAACGATCGCCAATATA	Δ*afcJ*, XbaI site
217 SOE R	CGGCCTGTCGCGTGGAGGCAGGGTTCCTGTCA	Δ*afcJ*
217 SOE F	TGACAGGAACCCTGCCTCCACGCGACAGGCCG	Δ*afcJ*
217 down EcoRI	gcgcGAATTCATGTTCAGCGCTTCCGGAT	Δ*afcJ*, EcoRI site
216 up XbaI	gcgcTCTAGACCCACCGTGTTCAACAAATA	Δ*afcK*, XbaI site
216 SOE R	CGCGAGCATGGTCGGAGAGGGGAGCTCCTGAA	Δ*afcK*
216 SOE F	TTCAGGAGCTCCCCTCTCCGACCATGCTCGCG	Δ*afcK*
216 down EcoRI	gcgcGAATTCCGAATCGTTCATCAGCAGCA	Δ*afcK*, EcoRI site
215_up_XbaI_F	gcgcTCTAGAAAGCCGGCAGTTGATCC	Δ*afcL*, XbaI site
215_SOE_R	AACGGGTCGCACCATGGGTCGGATCAGGCCGC	Δ*afcL*
215_SOE_F	GCGGCCTGATCCGACCCATGGTGCGACCCGTT	Δ*afcL*
215_down_EcoRI_R	GCAGCACACGCGGTTAT	Δ*afcL*
214 up XbaI	tataTCTAGAGCGACATCCGCACGTTCT	Δ*afcM*, XbaI site
214 SOE F	GACGGCGAGGCGTGACCATGAATGACACGCTT	Δ*afcM*
214 SOE R	AAGCGTGTCATTCATGGTCACGCCTCGCCGTC	Δ*afcM*

214 down EcoRI	gcgcGAATTCTTCGACGCACAGATCA	Δ*afcM*, EcoRI site
212_up_XbaI_F	gcgcTCTAGAACCTGTTCCTGCGAATCC	Δ*afcO*, XbaI site
212_SOE_R	GGGCGCTCAGGCGTCATCAGTTGAGCGGCATC	Δ*afcO*
212_SOE_F	GATGCCGCTCAACTGATGACGCCTGAGCGCCC	Δ*afcO*
212_down_EcoRI_R	gcgcGAATTCGTCATCCCGAAGTGGTCGT	Δ*afcO*, EcoRI site
211 up XbaI	gcgcTCTAGAATCATGATGGAGTGGCCGT	Δ*afcP*, XbaI site
211 SOE R	GATCGGCTCCTCGGGTTCAGGCGTCATGGCGG	Δ*afcP*
211 SOE F	CCGCCATGACGCCTGAACCCGAGGAGCCGATC	Δ*afcP*
211 down EcoRI	gcgcGAATTCGTGGATTGACCGGCACGAA	Δ*afcP*, EcoRI site
210_up_XbaI_F	gcgcTCTAGATTCGAACGCTATGCCGCC	Δ*afcQ*, XbaI site
210_SOE_R	AGCGTCGACAGGGTCATCAGGCCGGCCGCACG	Δ*afcQ*
210_SOE_F	CGTGCGGCCGGCCTGATGACCCTGTCGACGCT	Δ*afcQ*
210_down_EcoRI_R	gcgcGAATTCCCGATCCCTTCGTTGAGC	Δ*afcQ*, EcoRI site
rep_up_XbaI_F	gcgcTCTAGAGCGATGCGTTGCCCACCTACAT	Δ*rep*, XbaI site
rep_SOE_R	GTCGGGCGTCAGCGCGCCCATGCCGCGCGGCC	Δ*rep*
rep_SOE_F	GGCCGCGCGGCATGGGCGCGCTGACGCCCGAC	Δ*rep*
rep_down_EcoRI_R	gcgcGAATTCAACTGGCGGAAGAACGGGT	Δ*rep*, EcoRI site
209 up XbaI	gcgcTCTAGATCGAGGACGTGCTGTT	Δ*afcR*, XbaI site
209 SOE R	ACCTTGTATGCGCTCATCATGCGGCGGTCTCC	Δ*afcR*
209 SOE F	GGAGACCGCCGCATGATGAGCGCATACAAGGT	Δ*afcR*
209 down EcoRI	gcgcGAATTCGCCCATGAACATCTCG	Δ*afcR*, EcoRI site
207_up_XbaI_F	gcgcTCTAGACCAGTACTACCGCGACGAA	Δ*afcS*, XbaI site
207_SOE_R	CGAAAAGGGTGAGGGTGTCATTCCACCGCGGC	Δ*afcS*
207_SOE_F	GCCGCGGTGGAATGACACCCTCACCCTTTTCG	Δ*afcS*
207_down_EcoRI_R	cgcgGAATTCGGTACATCTCGGTCAGGC	Δ*afcS*, EcoRI site
205 up XbaI	gcgcTCTAGAGAGATGTACCGCGTGCTGG	Δ*afcU*, XbaI site
205 SOE R	CTCATCGTCGGCATCCCGCTCAGGCCAGTGCC	Δ*afcU*
205 SOE F	GGCACTGGCCTGAGCGGGATGCCGACGATGAG	Δ*afcU*
205 down EcoRI	gcgcGAATTCACTCGACCTTCTGCAATT	Δ*afcU*, EcoRI site
204_up_XbaI_F	gcgcTCTAGAGACGCGAACCCGAACCTG	Δ*afcV*, XbaI site
204_SOE_R	GGAACACCTTAGTCATCAGTCGAACAGCATGA	Δ*afcV*
204_SOE_F	TCATGCTGTTCGACTGATGACTAAGGTGTTCC	Δ*afcV*
204_down_SmaI_R	gcgcCCCGGGAGAATGCCGGTCGCGAAGAA	Δ*afcV*, SmaI site
201 up XbaI	gcgcTCTAGACGGCGATCGTGTTCCTGTT	Δ*afcF*, XbaI site
201 SOE R	GCCGTGAAGCGGCGCACGTCAGCTTGCCCGCG	Δ*afcF*
201 SOE F	CGCGGGCAAGCTGACGTGCGCCGCTTCACGGC	Δ*afcF*
201 down EcoRI	gcgcGAATTCTCAATCCTGCCGTCCATCCG	Δ*afcF*, EcoRI site
209 C up XhoI	atatCTCGAGATGACCCTGTCGACGC	pBBR-*afcR*, XhoI site
209 C down XbaI	cgcgTCTAGATCATGCGTCTCCTTCG	pBBR-*afcR*, XbaI site

a: SOE primers designed according to Horton *et al.*[27] Not underlined nucleotides at the 5' end are the overhangs.

Supplementary figures

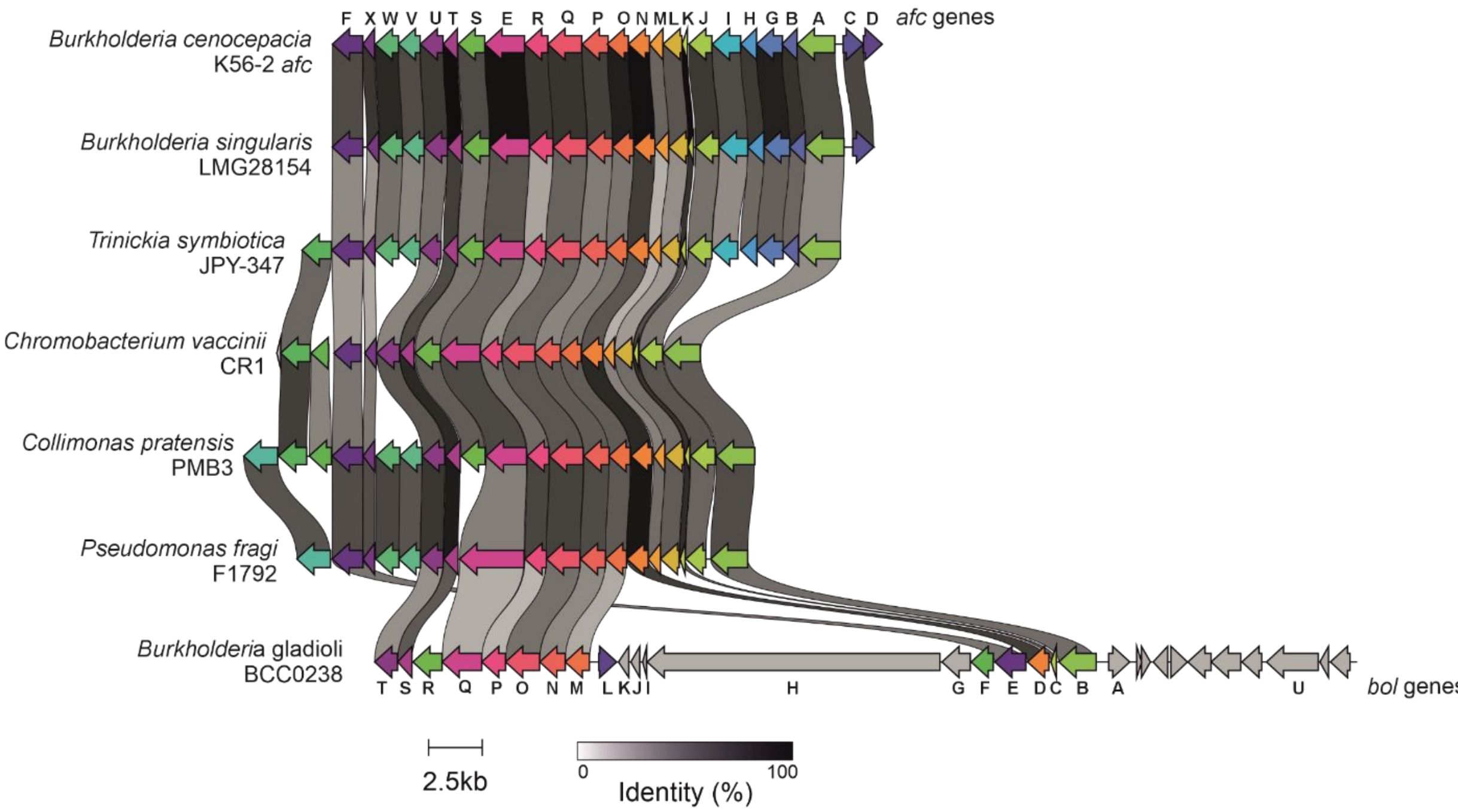

Figure S1. Alignment of the *B. cenocepacia* K56-2 *afc* gene cluster (top) and homologous gene clusters from diverse bacteria, including the *bol* cluster from *B. gladioli* BCC0238 (bottom). Gene designations for *afc* and *bol* are shown according to Zhong and Dashti, respectively.[6,28] Image generated using Clinker.[29]

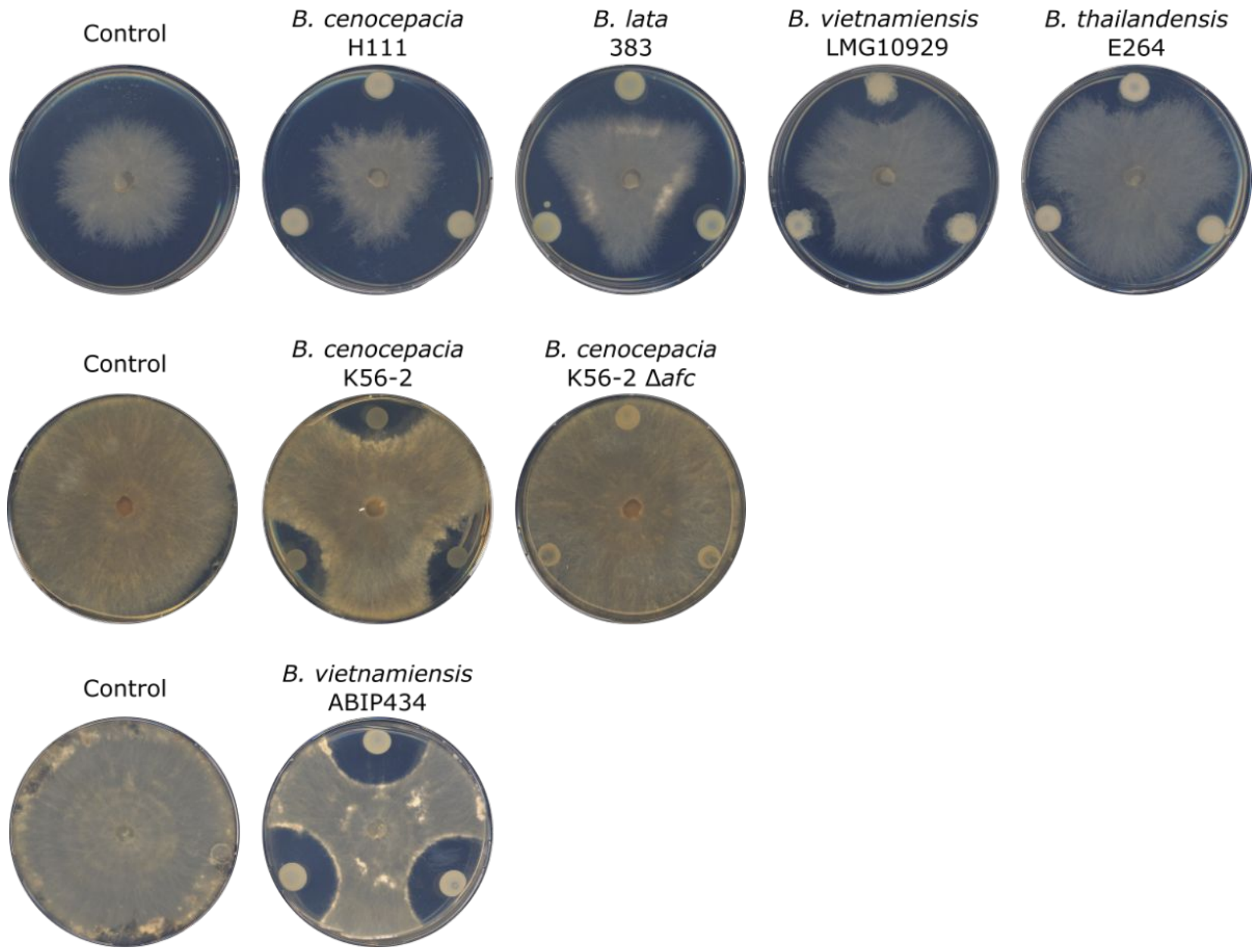

Figure S2. Antifungal activity of selected *Burkholderia* strains against *R. solani*.

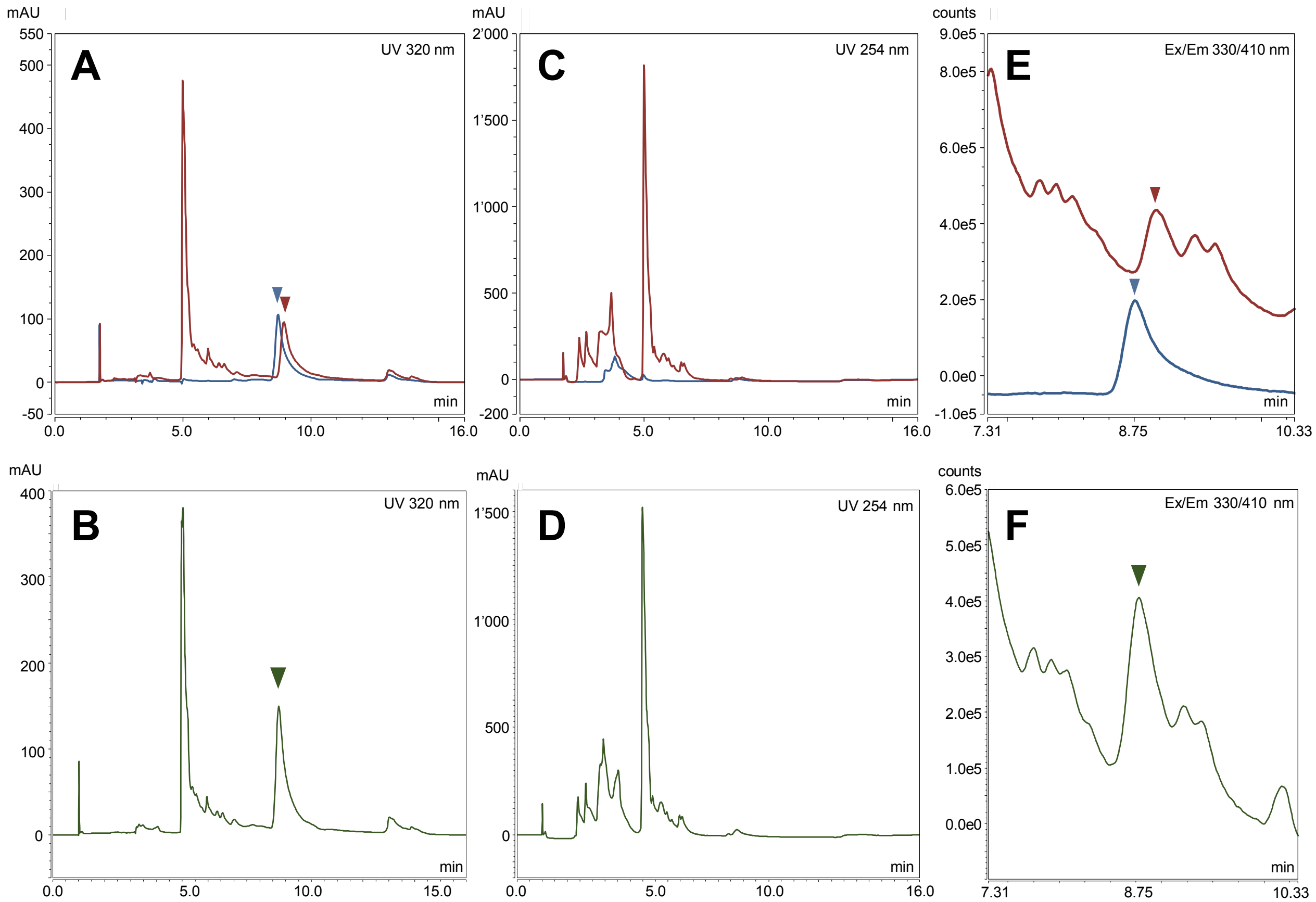

Figure S3. Comparison of the extraction methods described in this study (blue), in Zhong *et al.*[6] (maroon) and a 1:1 mixture of the two (green) by analytical HPLC. (**A-B**) UV absorption at λ = 320 nm (**C-D**) UV absorption at λ = 254 nm. (**E-F**) Enlarged emission peak at λ_{em} = 410 nm with λ_{ex} = 330 nm at the time of elution of AFC-BC11 (**1**). Peaks of AFC-BC11 are indicated with arrows.

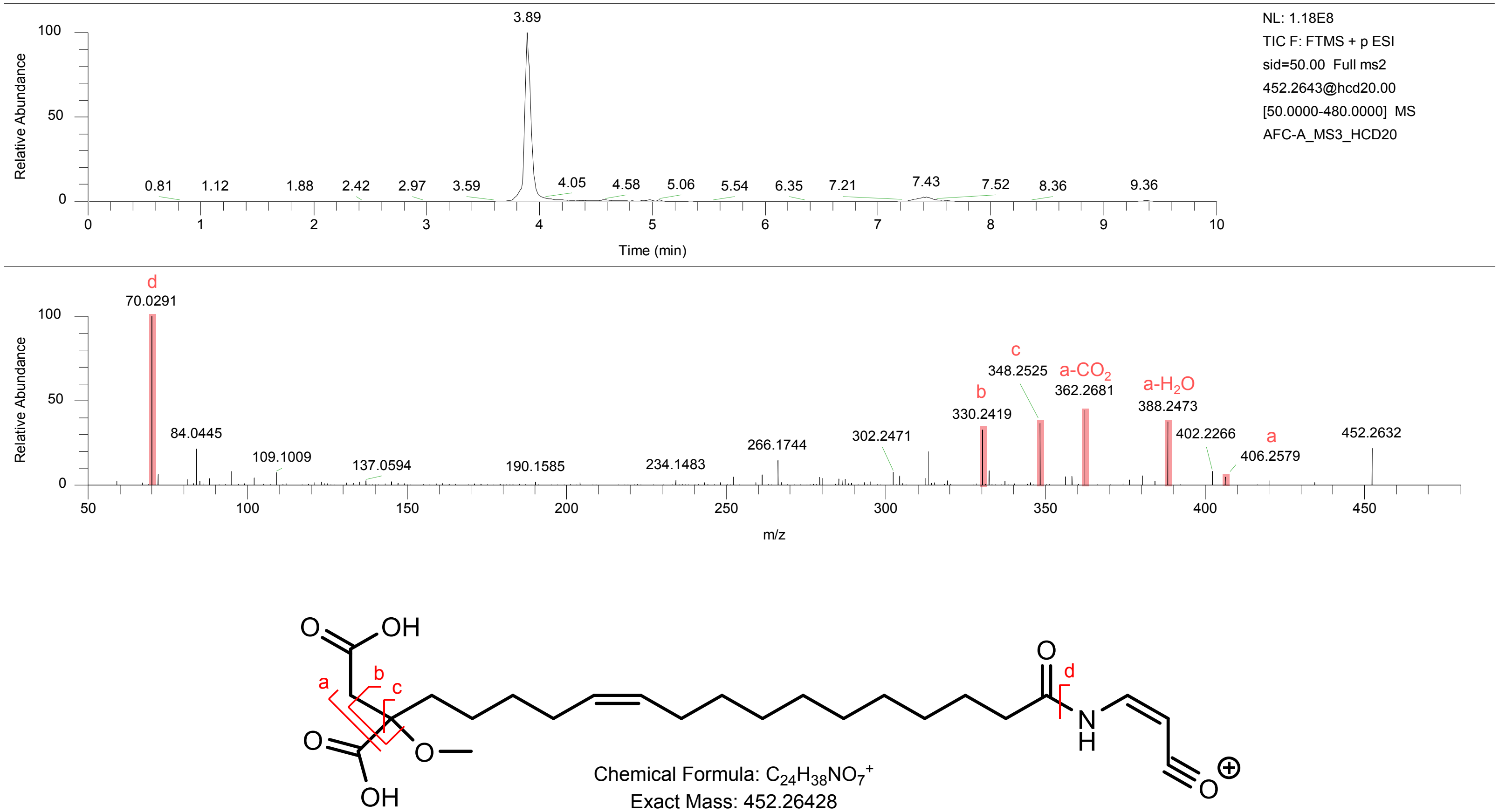

Figure S4. Pseudo-MS³ identification of the fatty acid tail fragment of AFC-BC11 (**1**). The identification of the functional group on the fatty acid tail was carried out by Q Exactive UHPLC-HR-MS/MS in positive mode. In-source fragmentation was applied, followed by fragmentation of the tail ion (*m/z* = 452.2643) with HCD at 20 eV. The identified fragments are shown in red.

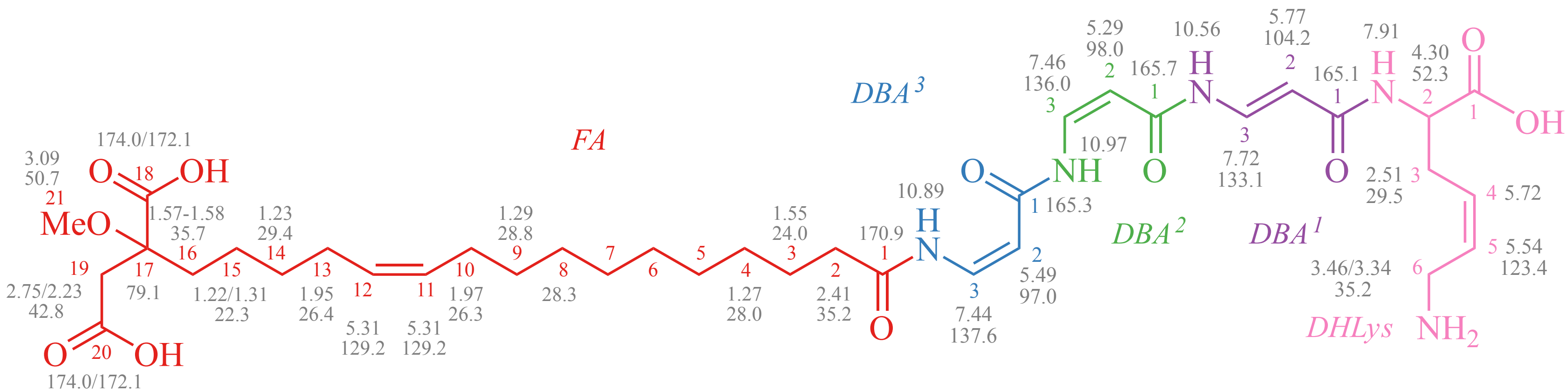

Figure S5. Summary of the ¹H and ¹³C-NMR chemical shift values for AFC-BC11 (**1,** DMSO-d_6, 600 and 1200 MHz).

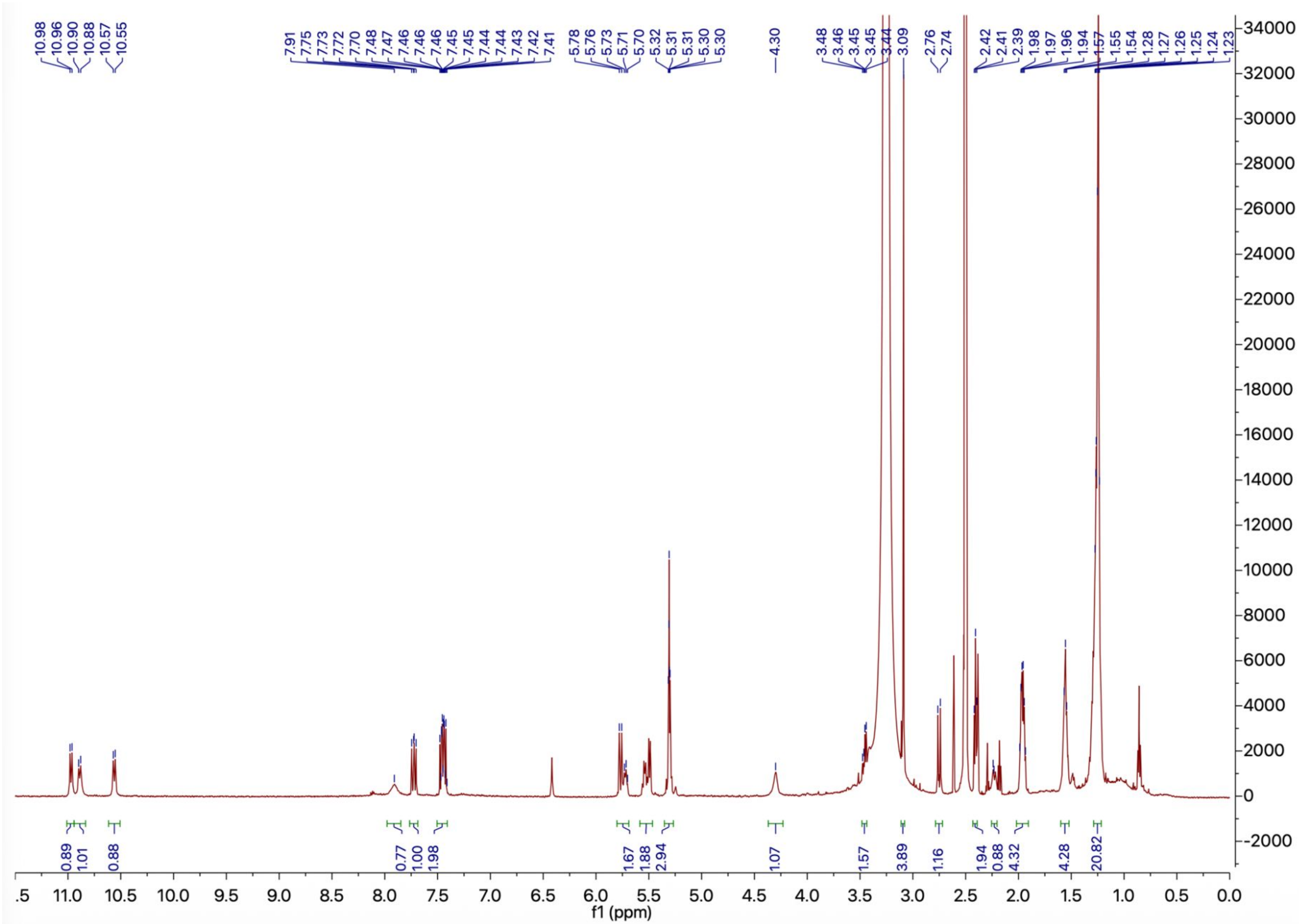

10.98
10.96
10.90
10.88
10.57
10.55
7.91
7.75
7.73
7.72
7.70
7.48
7.47
7.46
7.46
7.45
7.45
7.44
7.44
7.43
7.42
7.41
5.78
5.76
5.73
5.71
5.70
5.32
5.31
5.31
5.30
5.30
4.30
3.48
3.46
3.45
3.45
3.44
3.09
2.76
2.74
2.42
2.41
2.39
1.98
1.97
1.96
1.94
1.57
1.55
1.54
1.28
1.27
1.26
1.25
1.24
1.23
0.89
1.01
0.88
0.77
1.00
1.98
1.67
1.88
2.94
1.07
1.57
3.89
1.16
1.94
0.88
4.32
4.28
20.82
f1 (ppm)

Figure S6. ^{1}H NMR Spectrum of AFC-BC11 (**1**), (DMSO-d_6, 600 MHz, 313K)

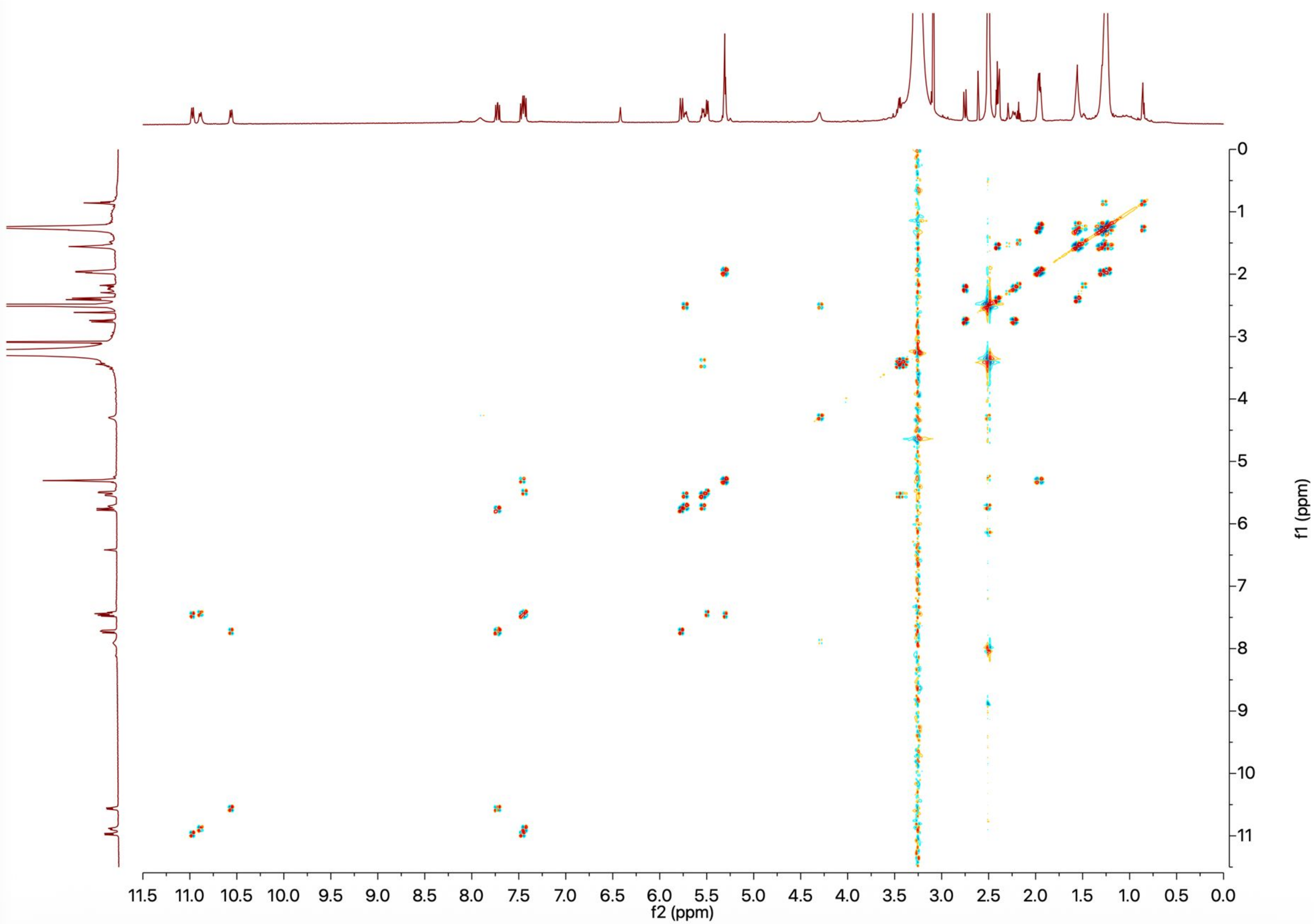

Figure S7. ^{1}H–^{1}H COSY Spectrum of AFC-BC11 (**1**), (DMSO-d_6, 600 MHz, 313K)

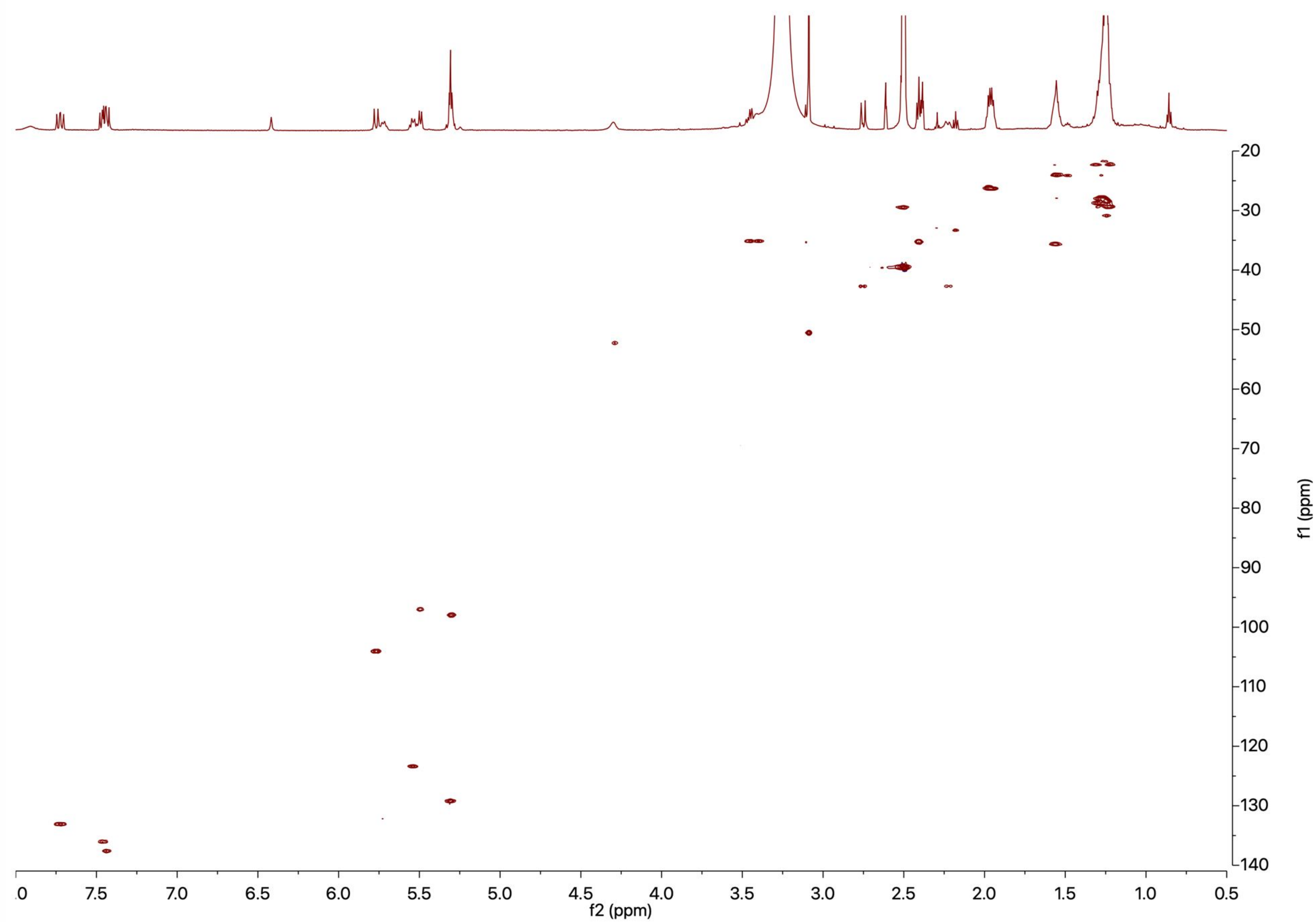
f2 (ppm)
f1 (ppm)

Figure S8. ^{1}H–^{13}C HSQC Spectrum of AFC-BC11 (**1**), (DMSO-d_6, 600 MHz, 313K)

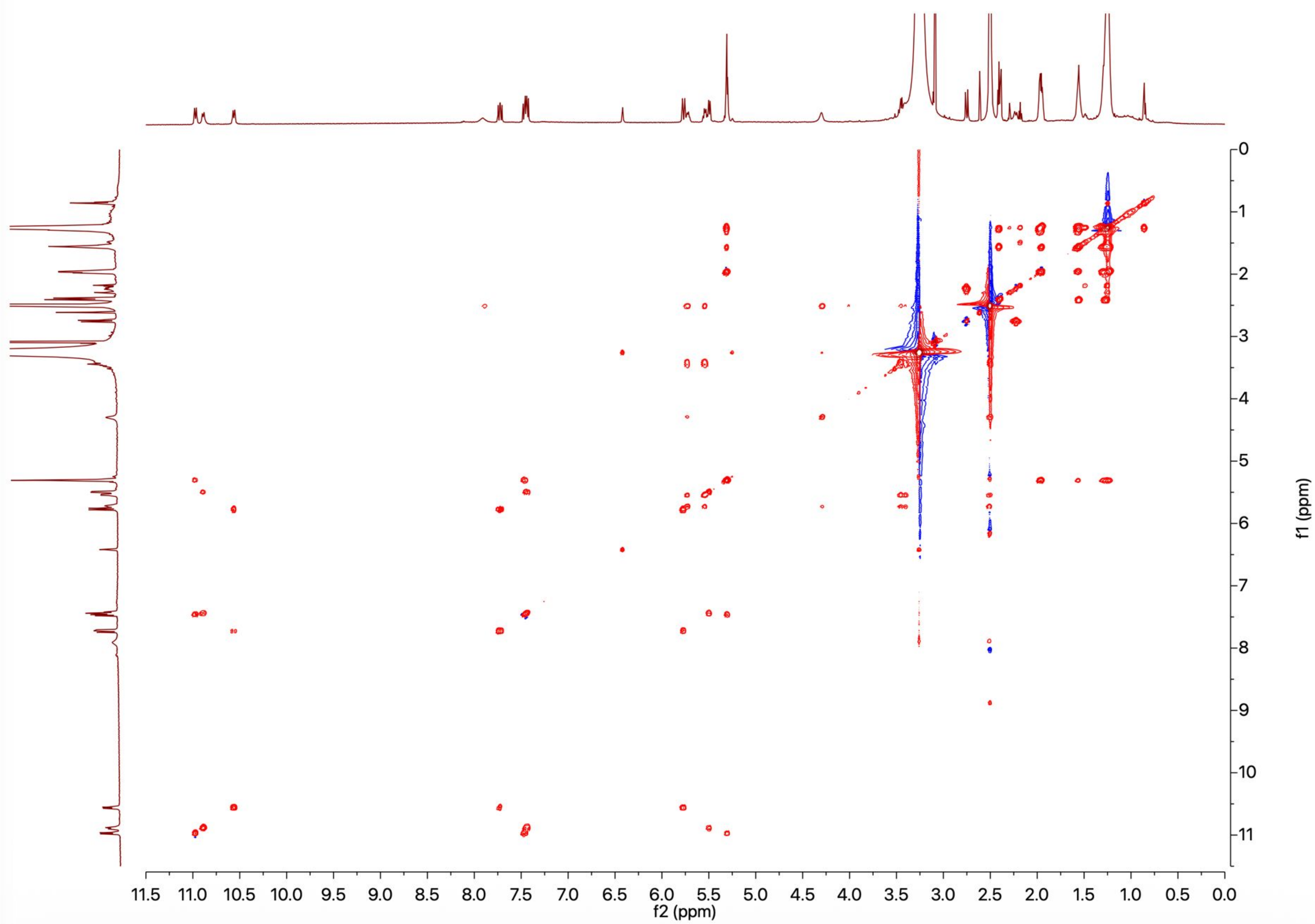

Figure S9. ^{1}H–^{1}H TOCSY Spectrum (100 ms) of AFC-BC11 (**1**), (DMSO-d_6, 600 MHz, 313K)

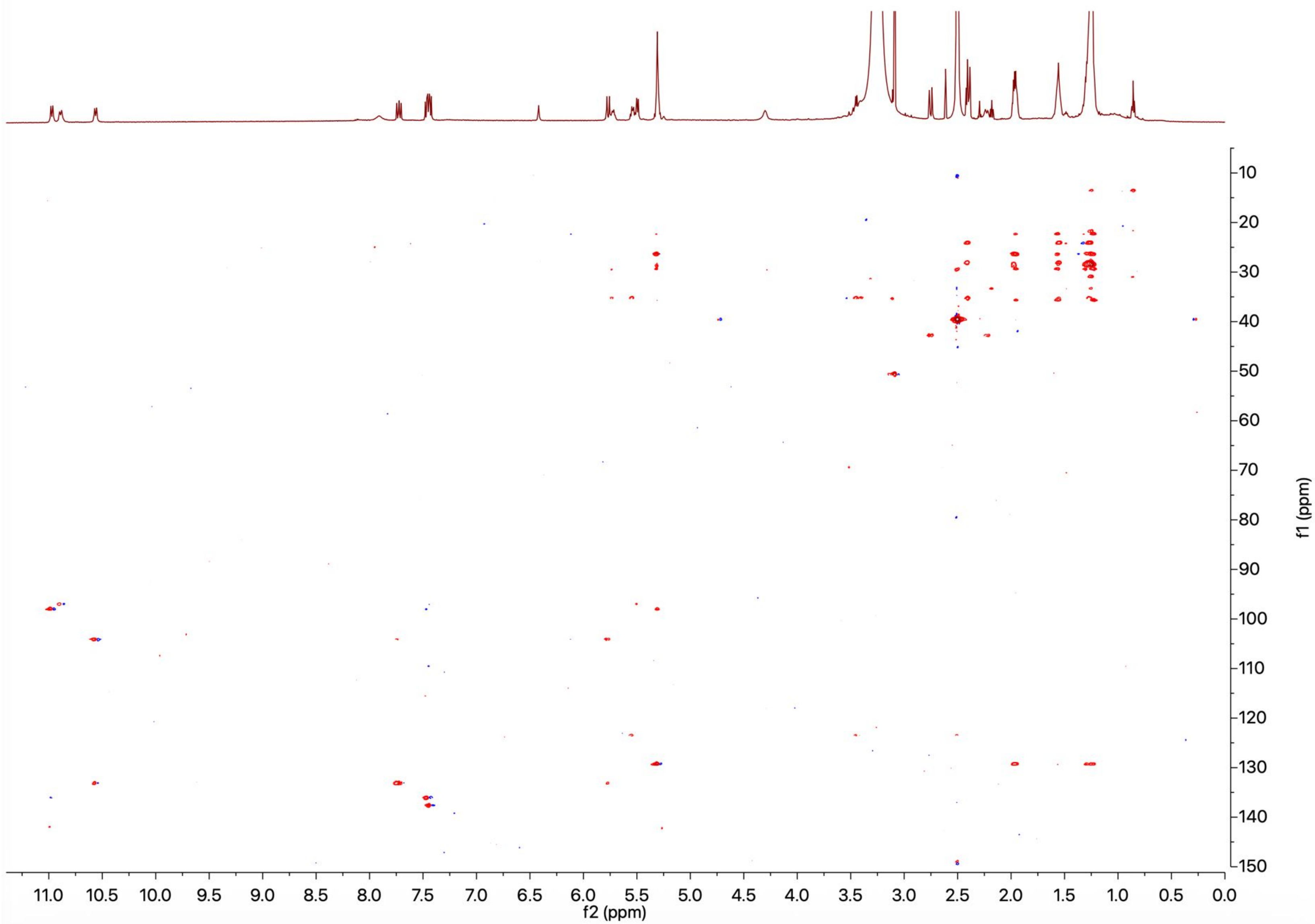

Figure S10. ^{1}H–^{13}C HSQC-TOCSY Spectrum (60 ms) of AFC-BC11 (**1**), (DMSO-d_6, 600 MHz, 313K)

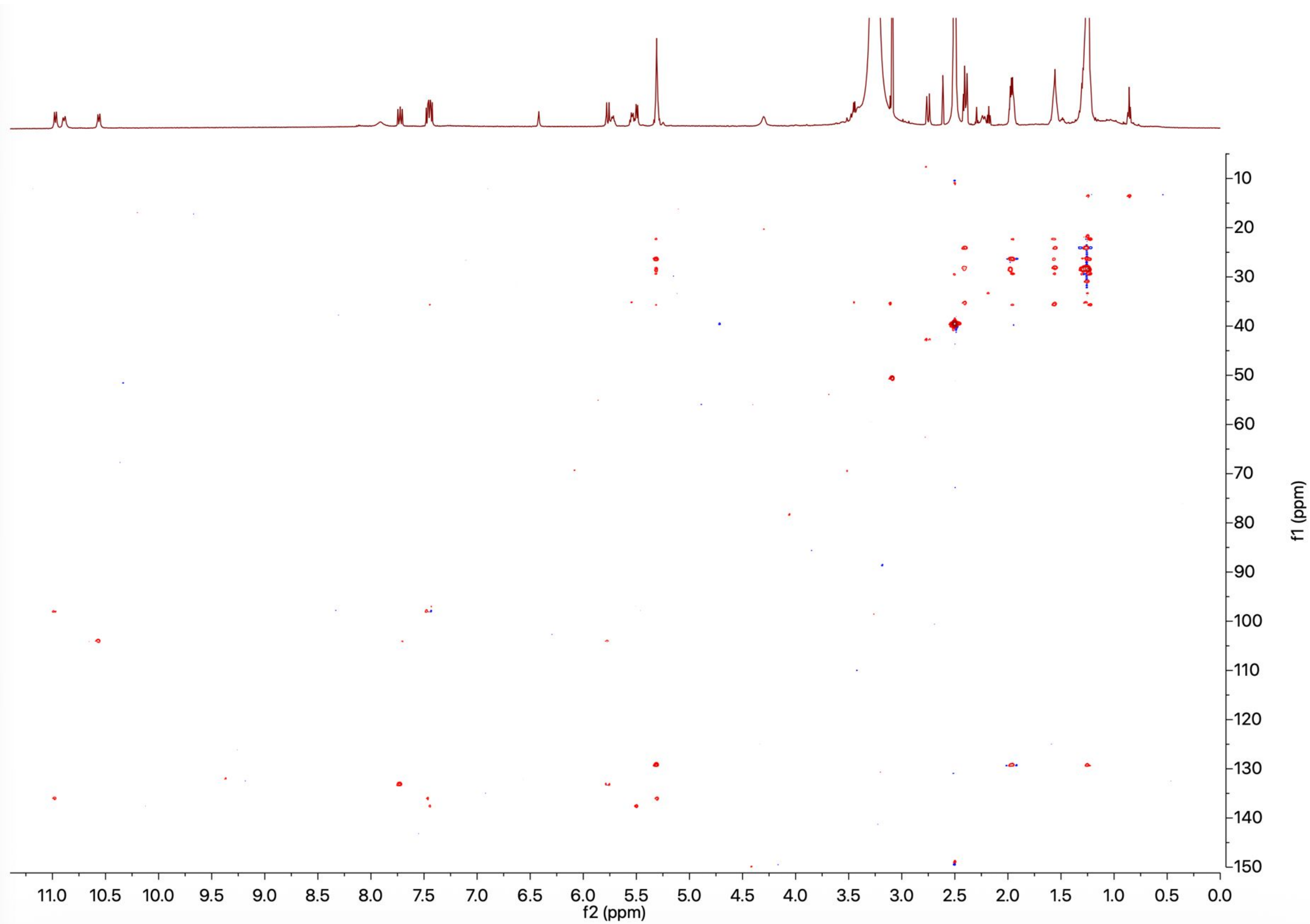

Figure S11. ^{1}H–^{13}C HSQC-TOCSY Spectrum (90 ms) of AFC-BC11 (**1**), (DMSO-d_6, 600 MHz, 313K)

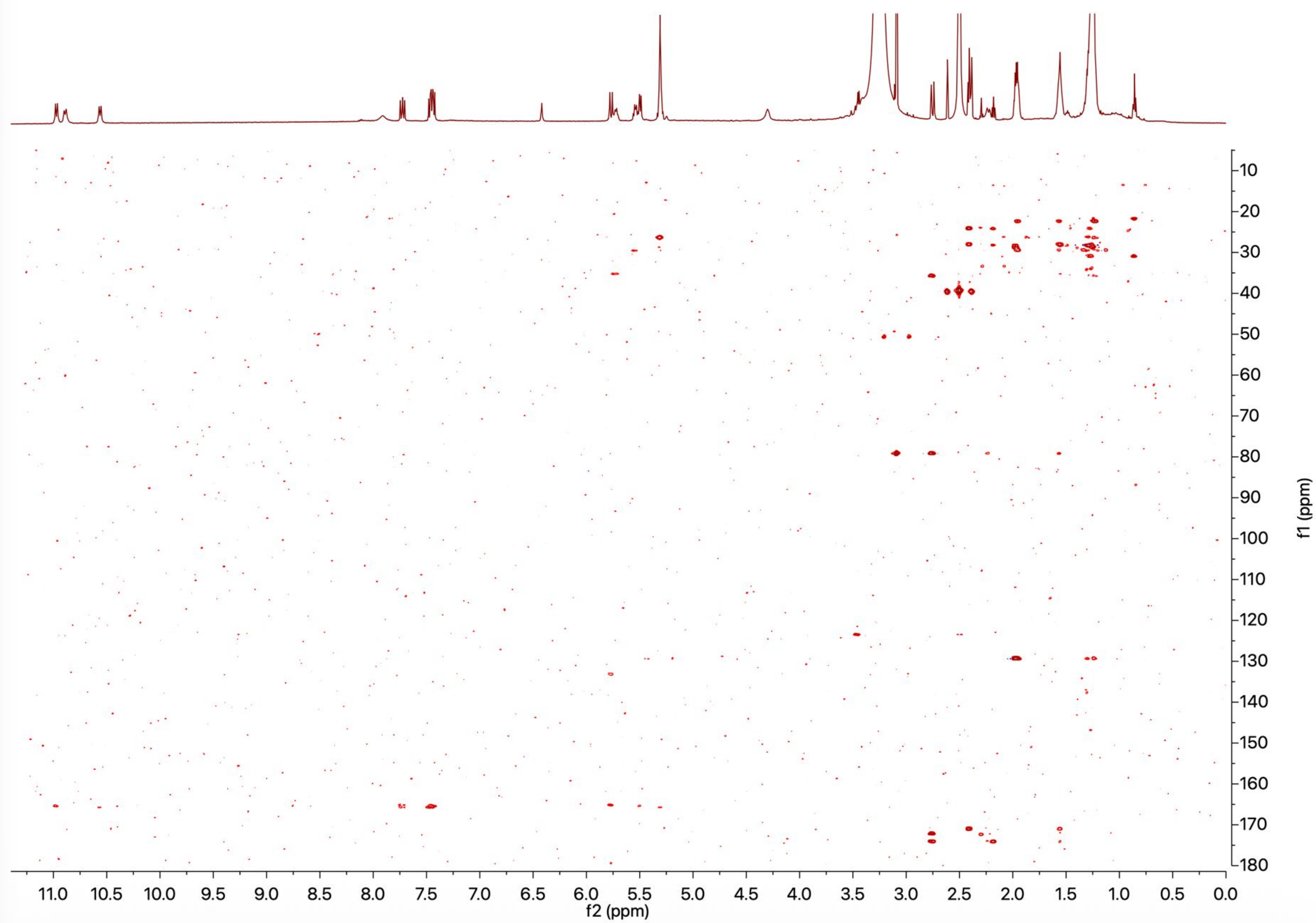

Figure S12. ^1H–^{13}C HMBC Spectrum of AFC-BC11 (**1**), (DMSO-d_6, 600 MHz, 313K)

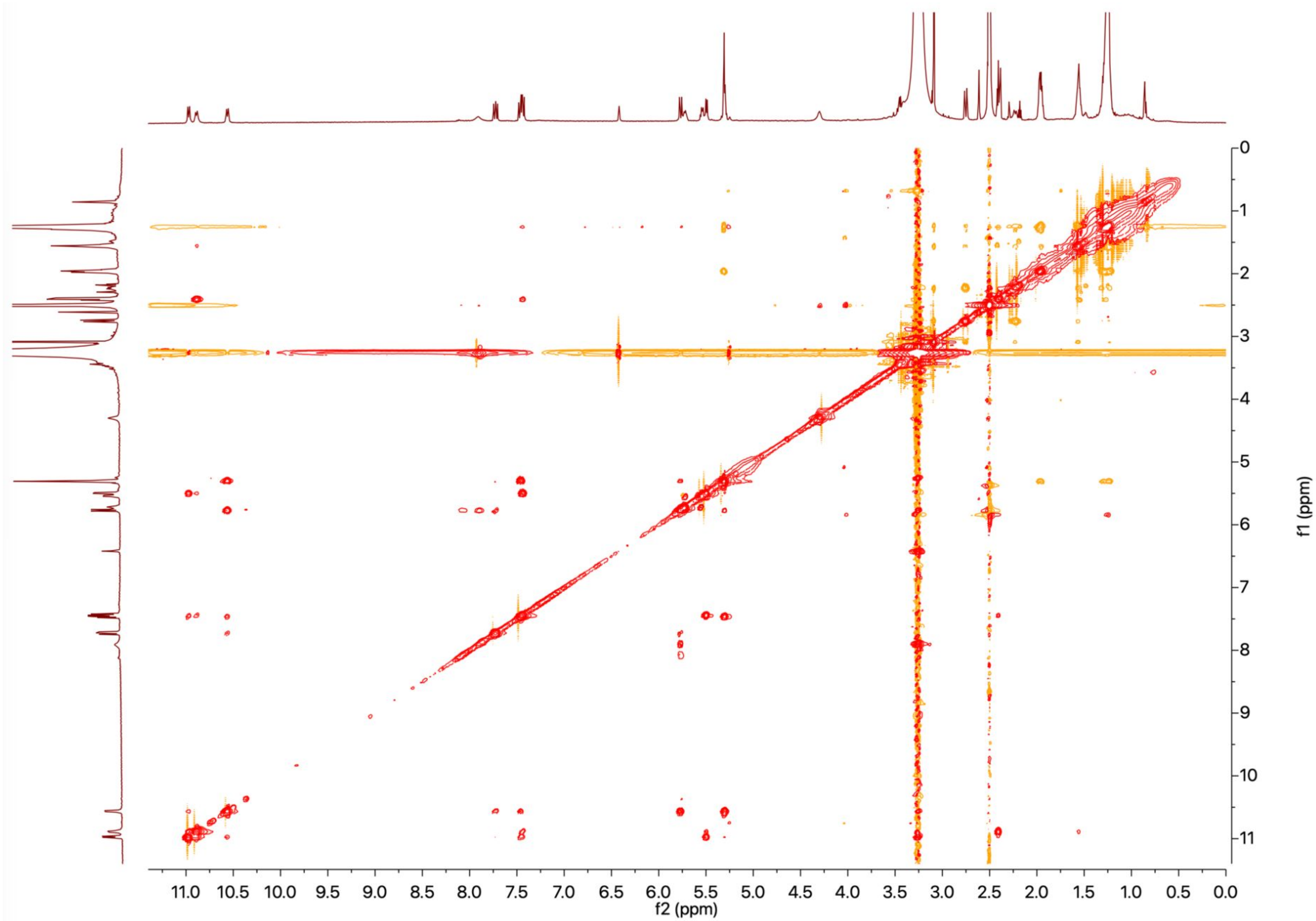

Figure S13. $^1H–^1H$ NOESY Spectrum of AFC-BC11 (**1**), (DMSO-d_6, 600 MHz, 313K)

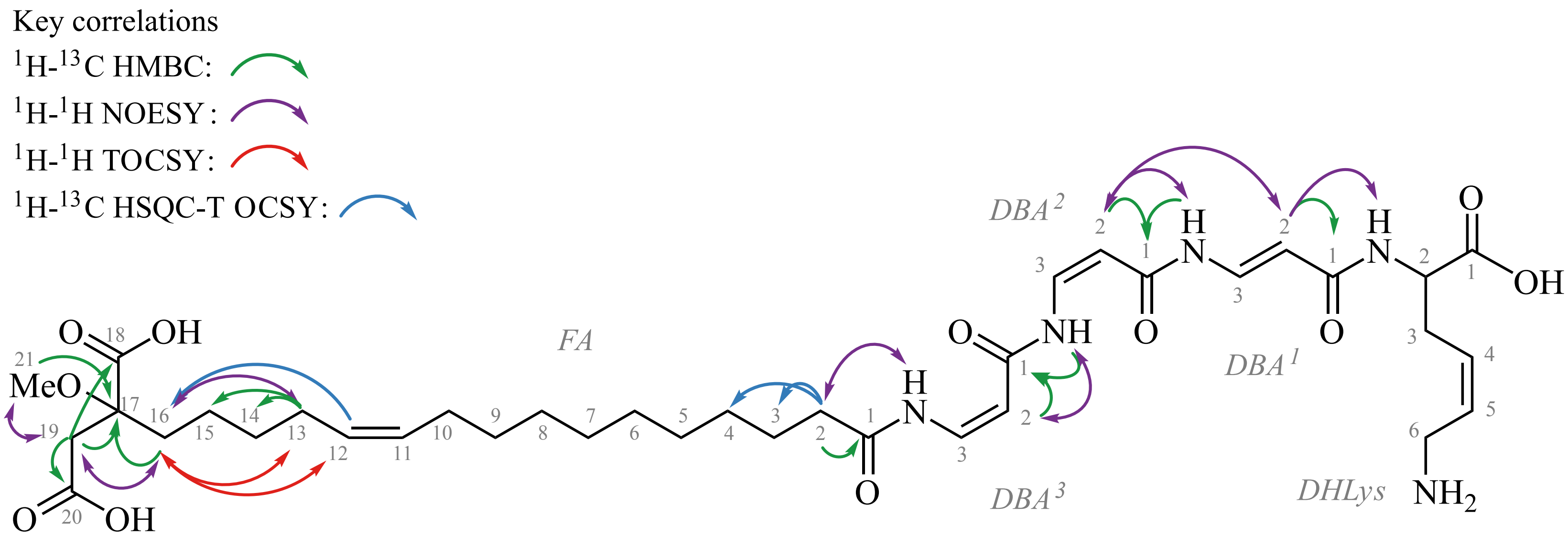

Figure S14. Key correlations for the structural elucidation of AFC-BC11 (**1**).

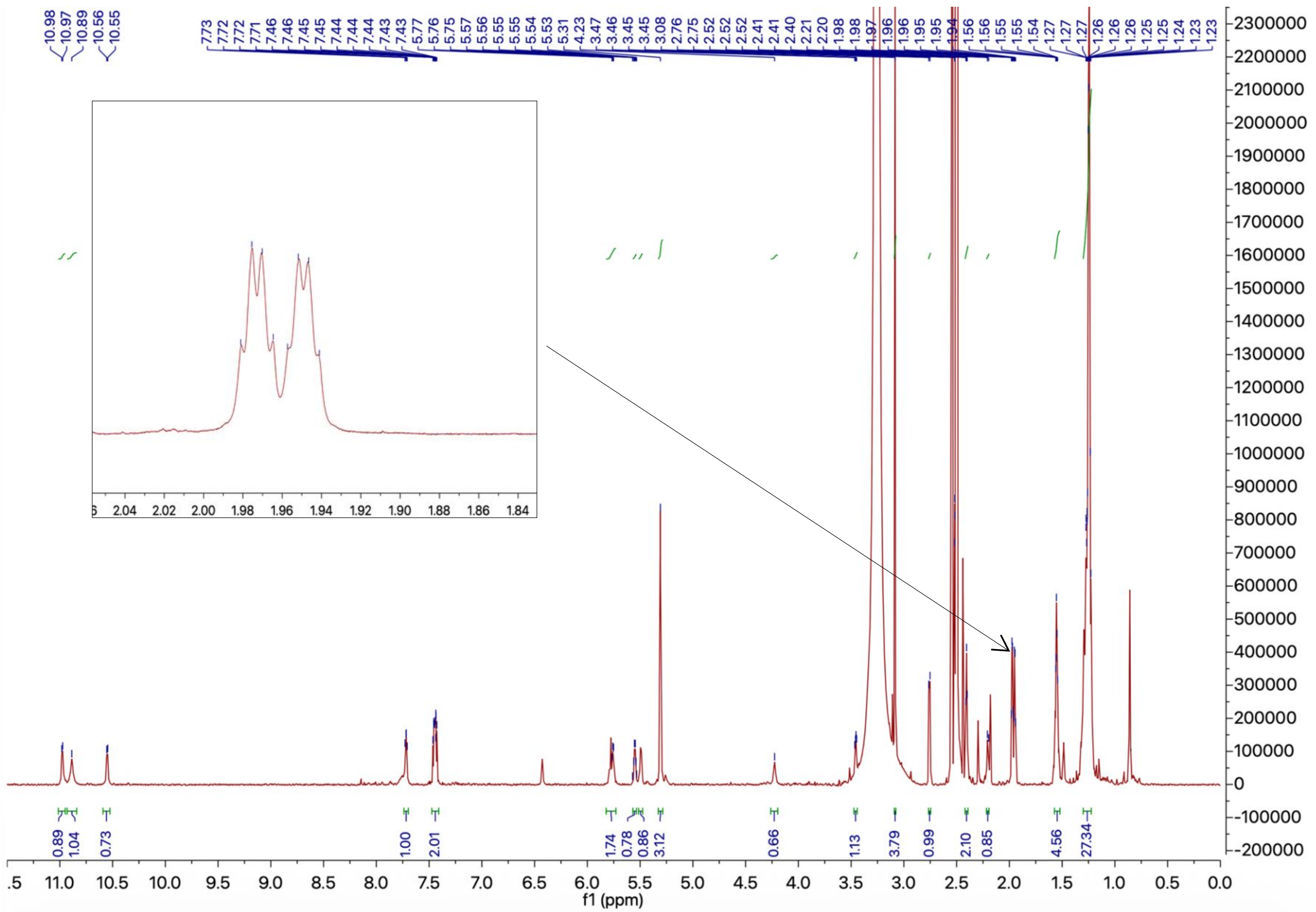

Figure S15. ^{1}H NMR Spectrum of AFC-BC11 (**1**), (DMSO-d_6, 1.2 GHz, 313K) with a highlighted region showing the resonances of protons H-10 and H-13.

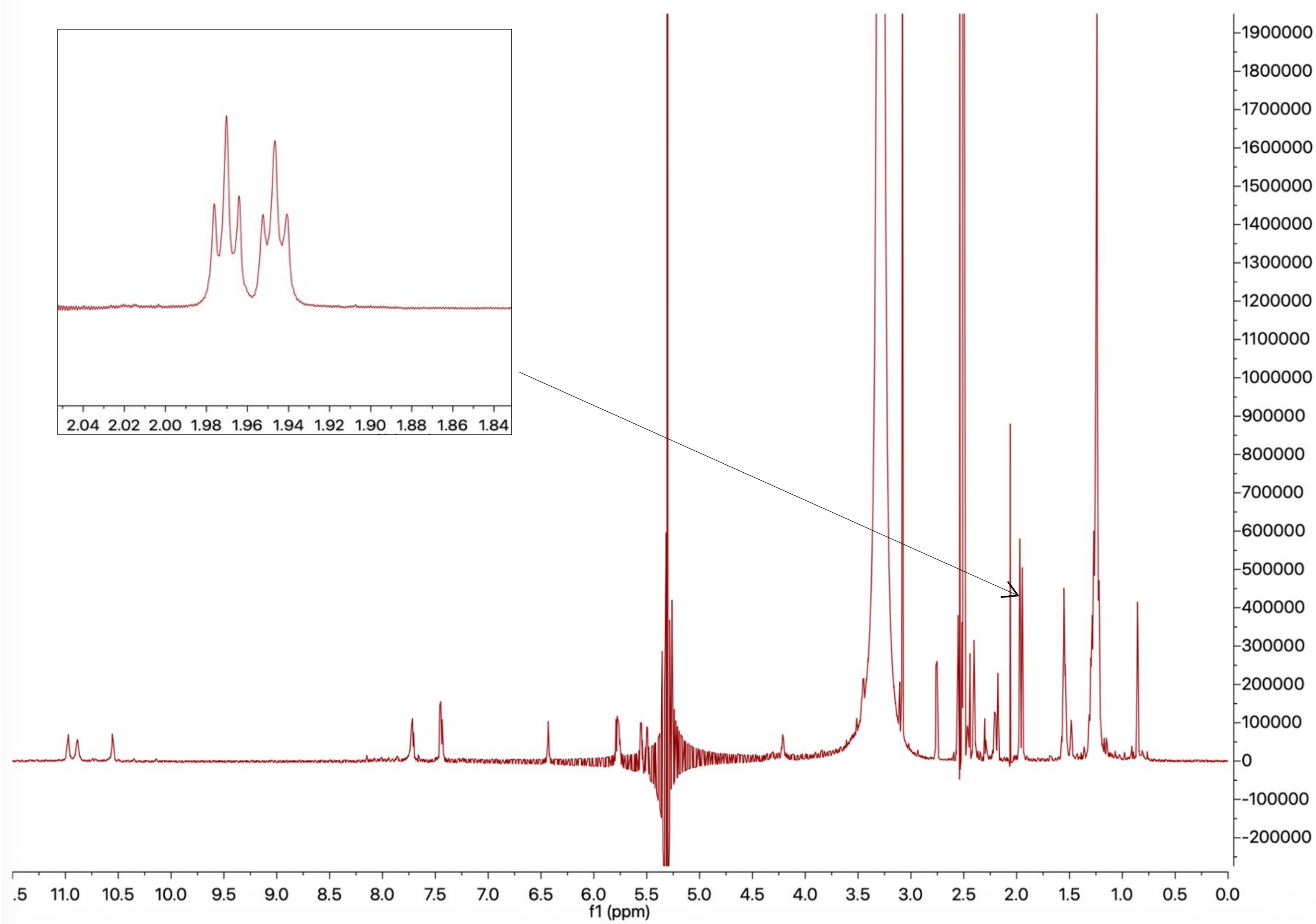

f1 (ppm)

Figure S16. ^{1}H NMR Spectrum recorded with homodecoupling at 5.3 ppm of AFC-BC11 (**1**), (DMSO-d_6, 1.2 GHz, 313K) with a highlighted region showing the resonances of protons H-10 and H-13.

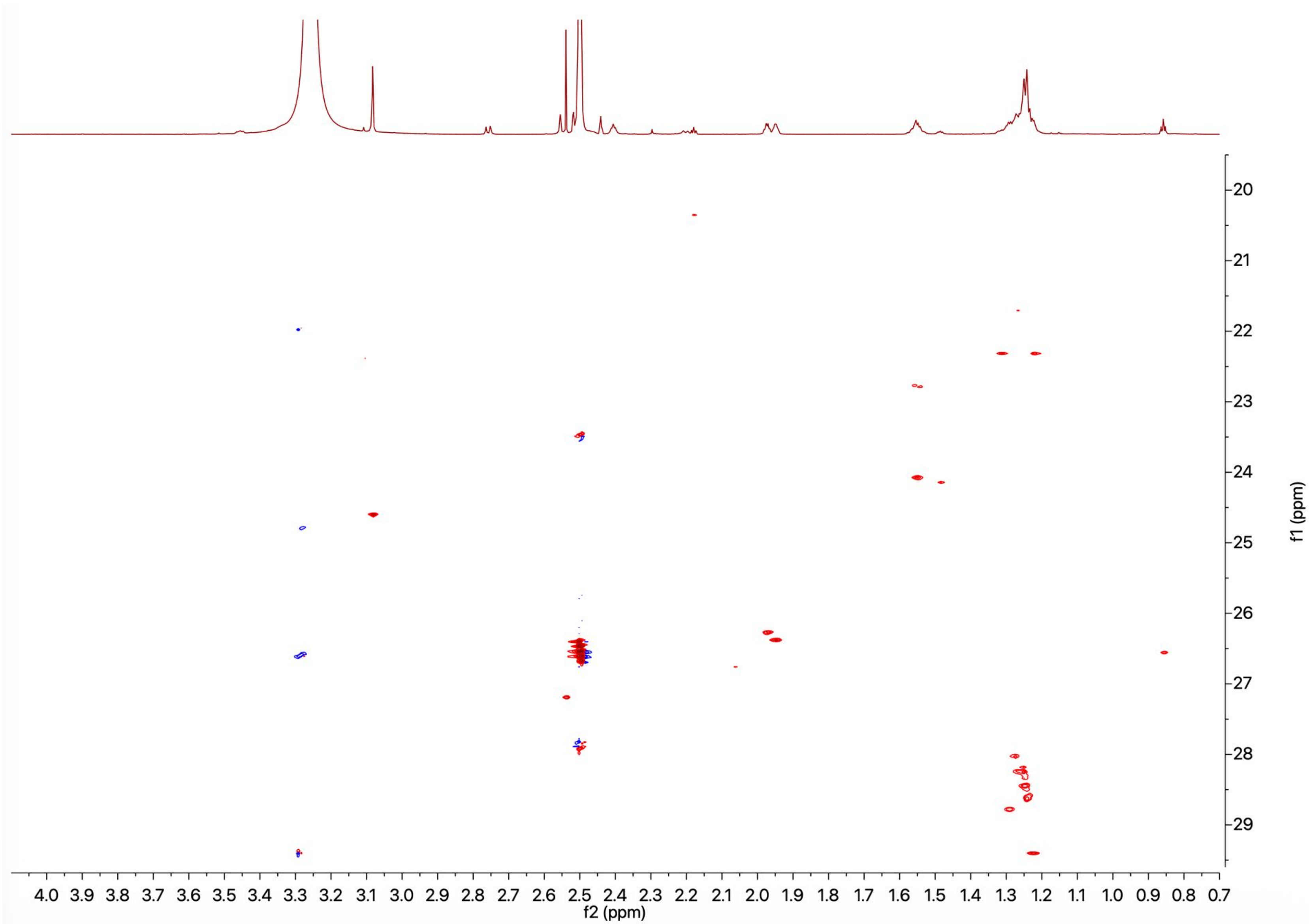

Figure S17. ^{1}H–^{13}C HSQC Spectrum of AFC-BC11 (**1**) in high resolution, (DMSO-d_6, 1.2 GHz, 313K)

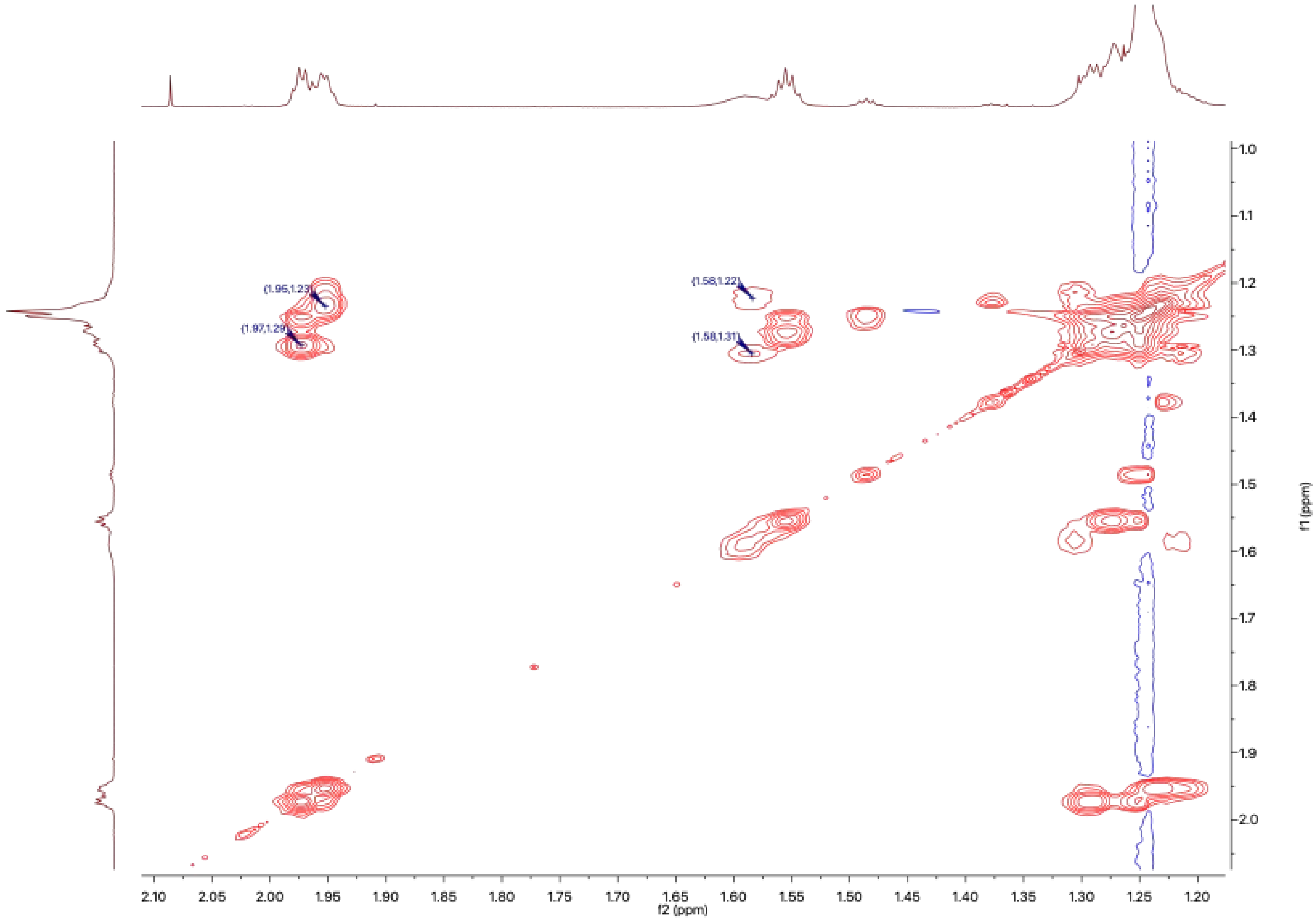

Figure S18. Highlighted region of the ^{1}H–^{1}H CLIP COSY spectrum of AFC-BC11 (**1**) recorded in high resolution, (DMSO-d_6, 1.2 GHz, 313K). The assignments of the highlighted correlations for the FA part are: 1.95,1.23 ppm for H13 to H14; 1.97,1.29 ppm for H10 to H9; 1.58,1.22 ppm for H16 to H15a; 1.58,1.31 ppm for H16 to H15b.

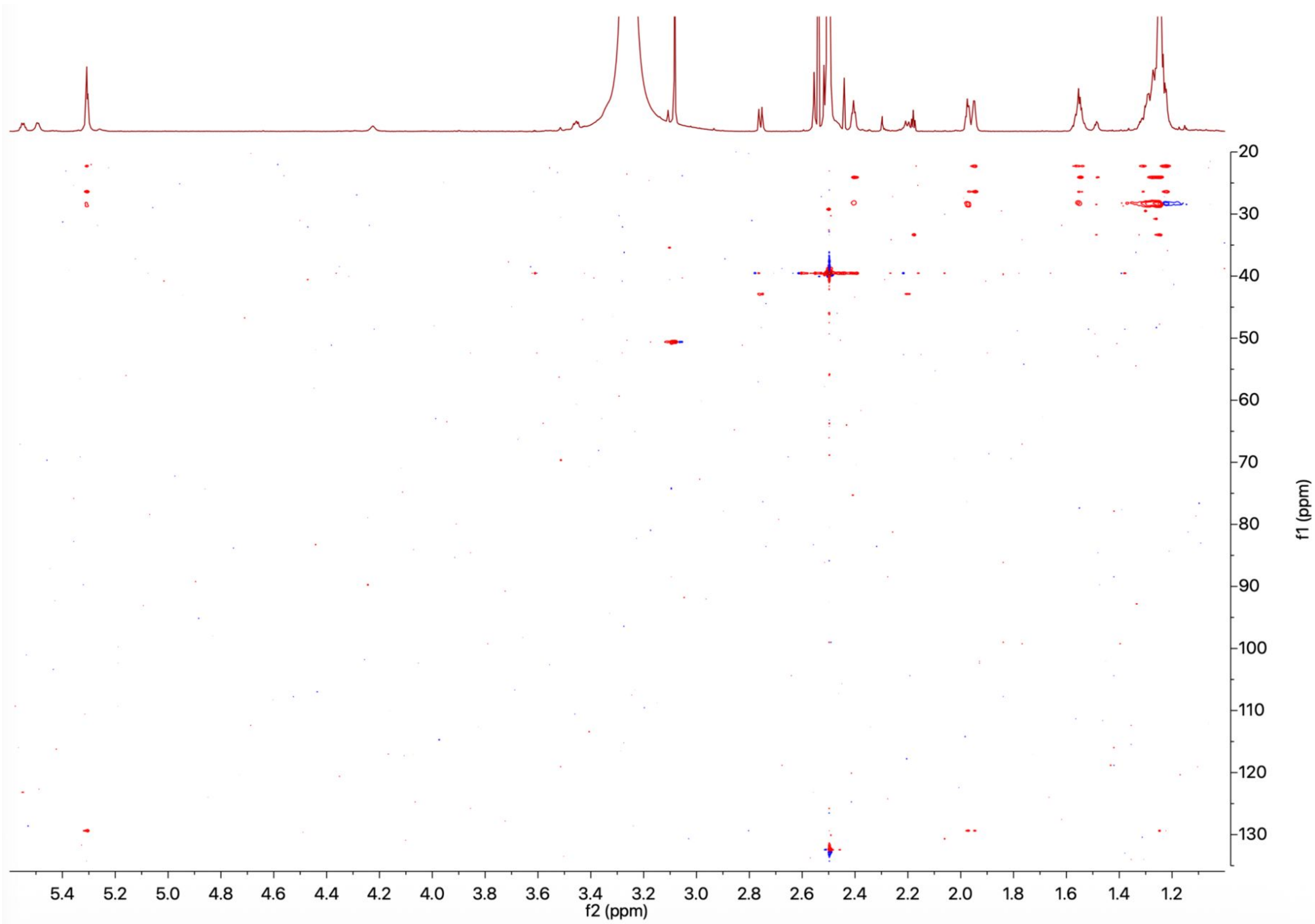

Figure S19. ^{1}H–^{13}C HSQC-TOCSY Spectrum (100 ms) of AFC-BC11 (**1**), (DMSO-d_6, 1.2 GHz, 313K) with the part of the spectrum showing the key correlations for the fatty acid moiety enlarged.

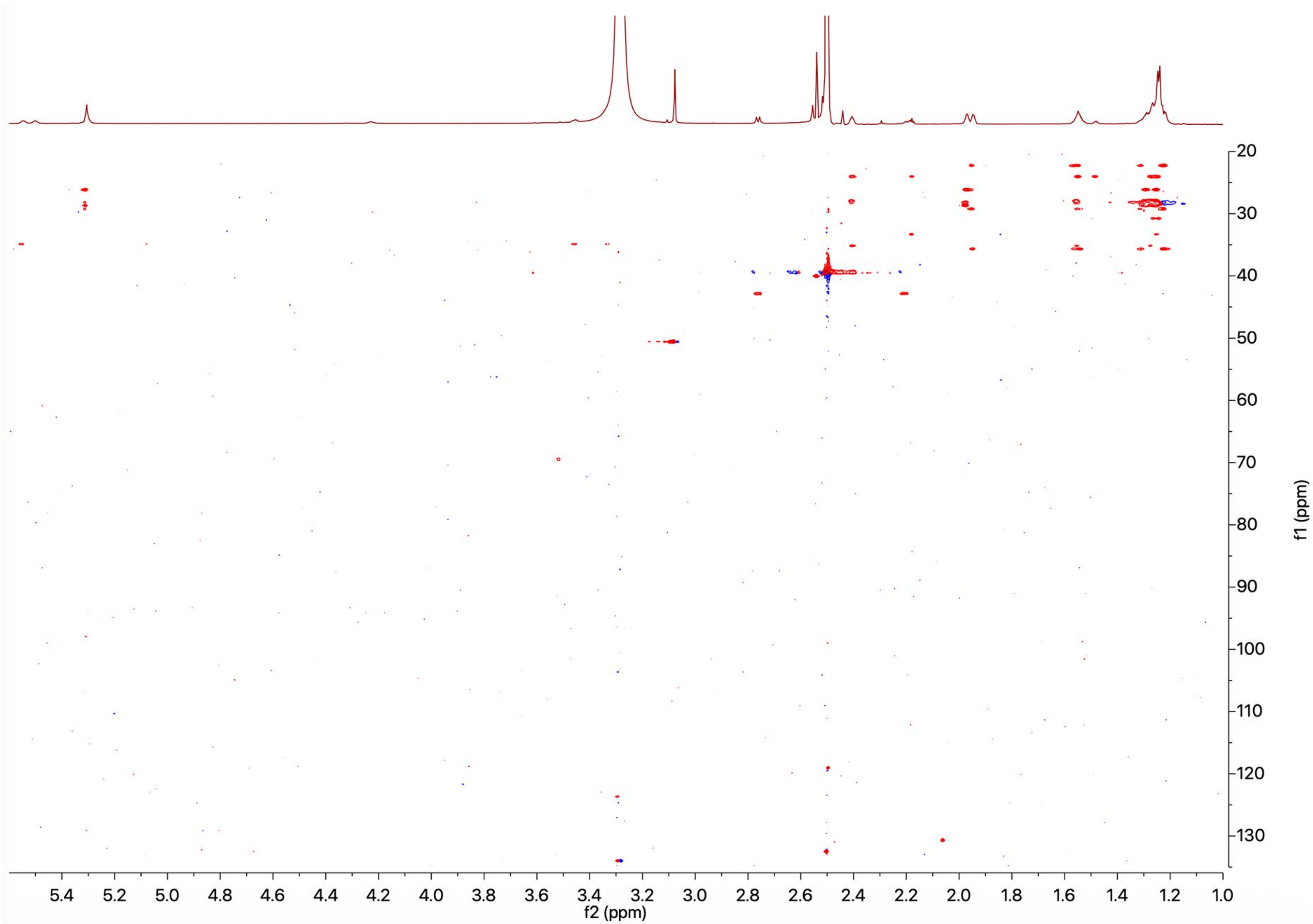

f2 (ppm)
f1 (ppm)

Figure S20. ^{1}H–^{13}C HSQC-TOCSY Spectrum (60 ms) of AFC-BC11 (**1**), (DMSO-d_6, 1.2 GHz, 313K) the part of the spectrum showing the key correlations for the fatty acid moiety has been enlarged.

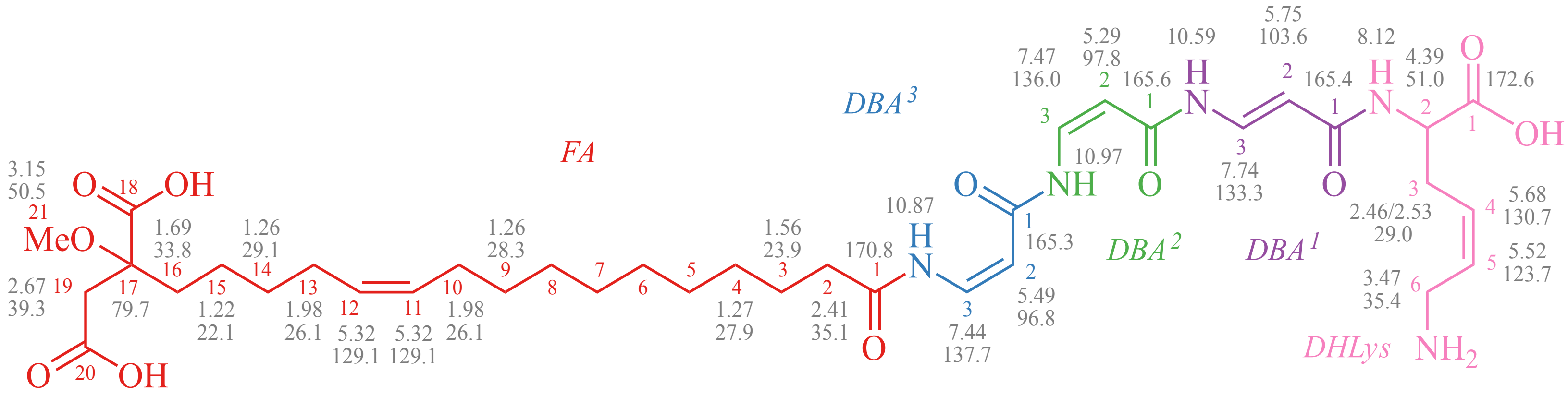

Figure S21. Summary of chemical shifts for AFC-BC11 using the sample obtained from LB medium.

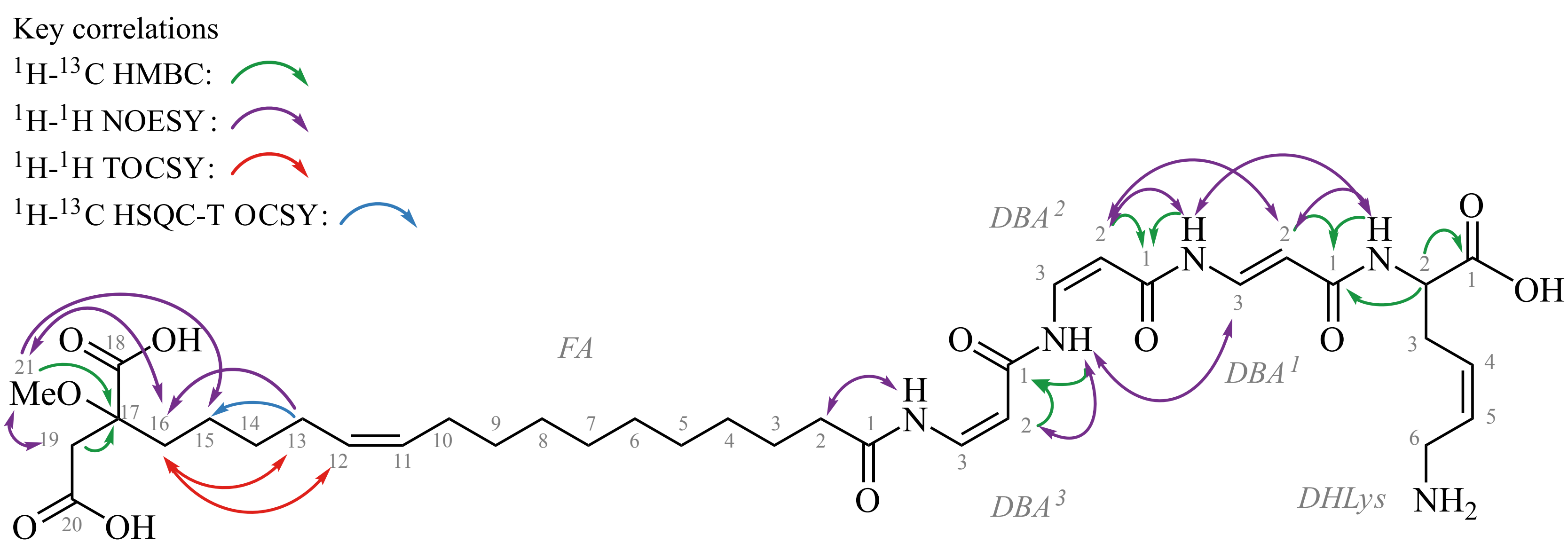

Figure S22. Key correlations used for the structure elucidation of AFC-BC11 using the sample obtained from LB medium.

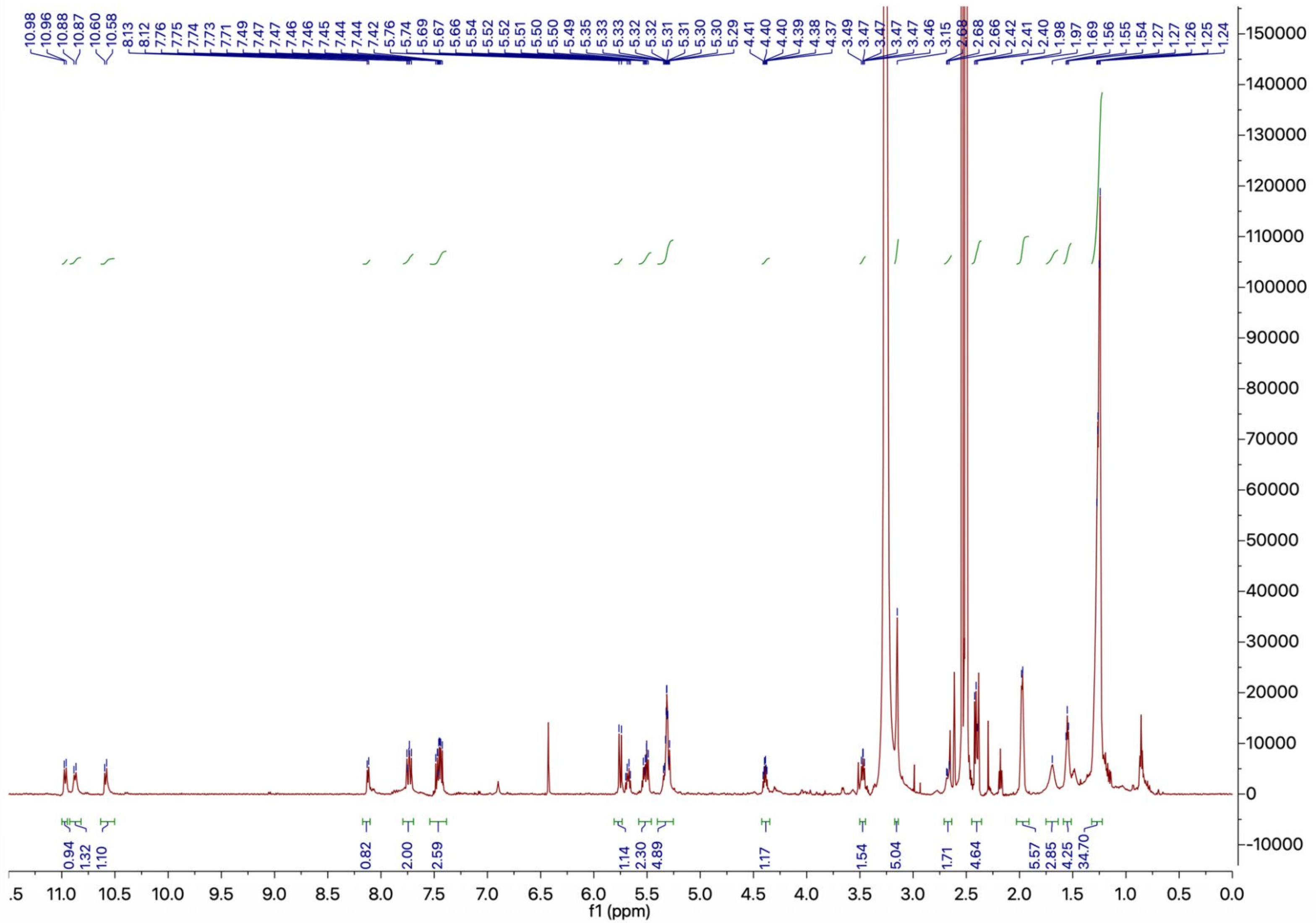
10.98 10.96 10.88 10.87 10.60 10.58 8.13 8.12 7.76 7.75 7.74 7.73 7.71 7.49 7.47 7.47 7.46 7.46 7.45 7.44 7.44 7.42 5.76 5.74 5.69 5.67 5.66 5.54 5.52 5.52 5.51 5.50 5.50 5.49 5.35 5.33 5.33 5.32 5.32 5.31 5.31 5.30 5.29 4.41 4.40 4.40 4.39 4.38 4.37 3.49 3.47 3.47 3.47 3.47 3.46 3.15 2.68 2.68 2.66 2.42 2.41 2.40 1.98 1.97 1.69 1.56 1.55 1.54 1.27 1.26 1.25 1.24
0.94 1.32 1.10 0.82 2.00 2.59 1.14 2.30 4.89 1.17 1.54 5.04 1.71 4.64 5.57 2.85 4.25 34.70
f1 (ppm)

Figure S23. ^{1}H NMR Spectrum of AFC-BC11 obtained from LB medium, (DMSO-d_6, 600 MHz)

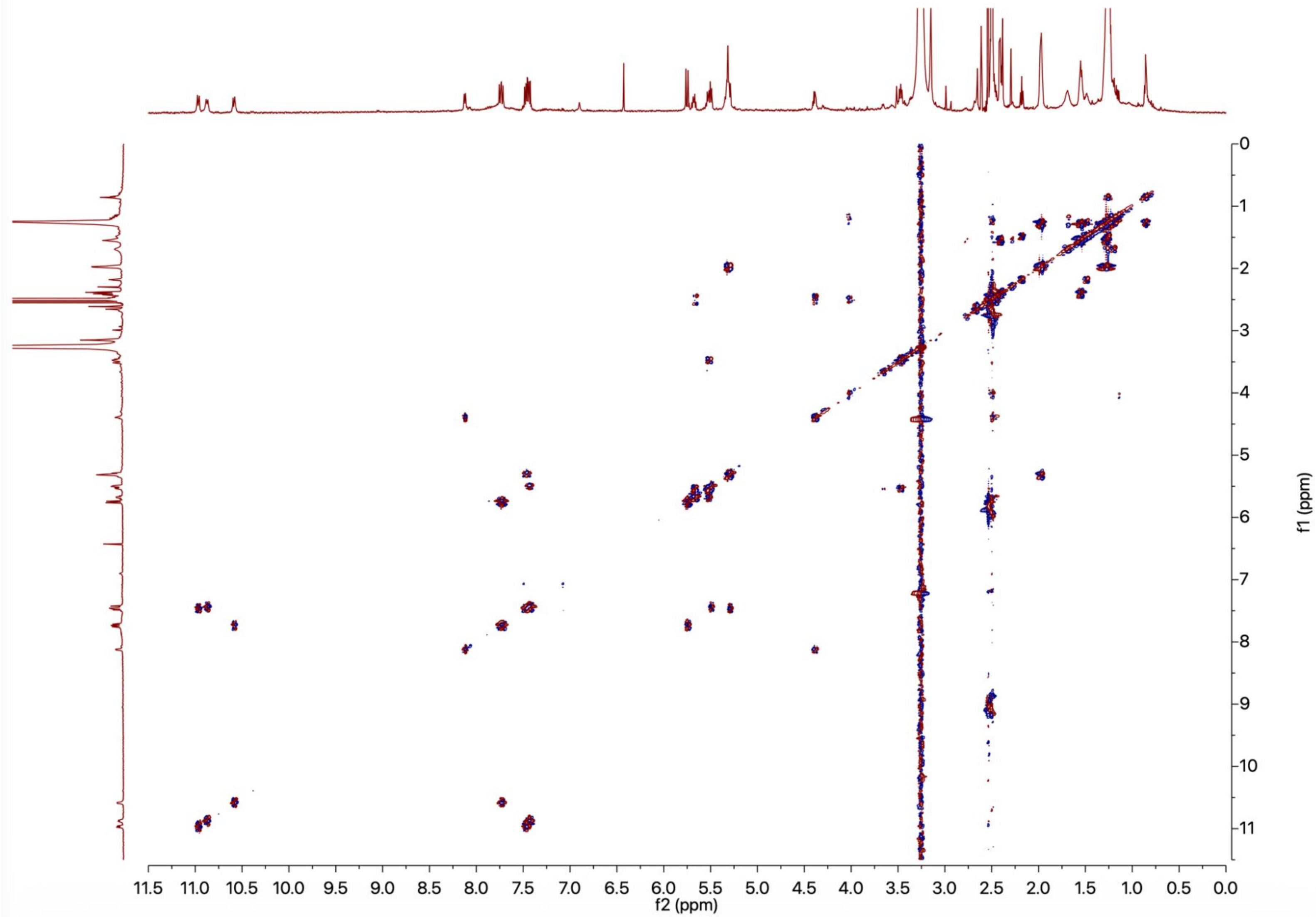

Figure S24. ^{1}H–^{1}H COSY Spectrum of AFC-BC11 obtained from LB medium, (DMSO-d_6, 600 MHz, 313K)

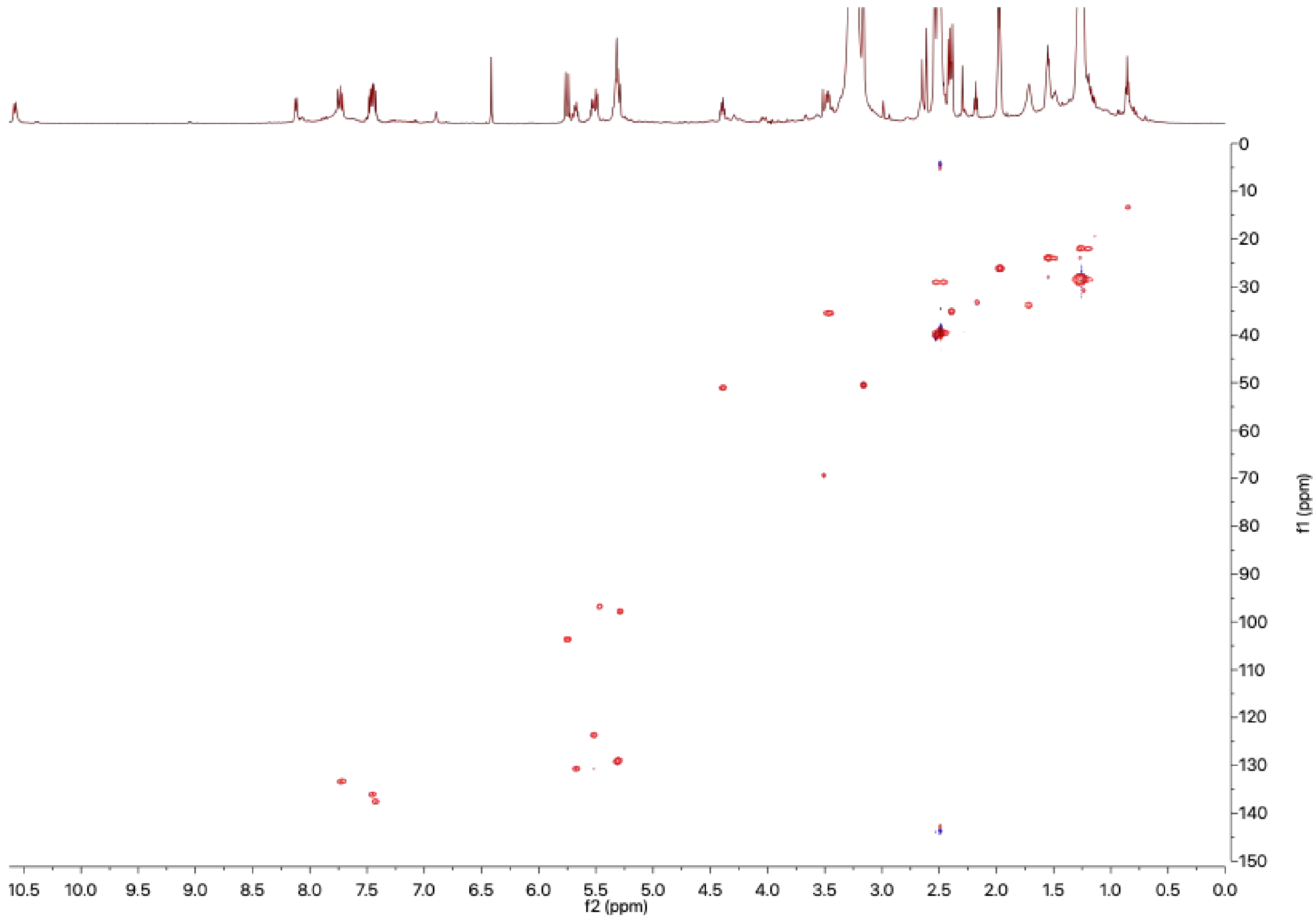

Figure S25. ^{1}H–^{13}C HSQC Spectrum of AFC-BC11 obtained from LB medium, (DMSO-d_6, 600 MHz, 313K)

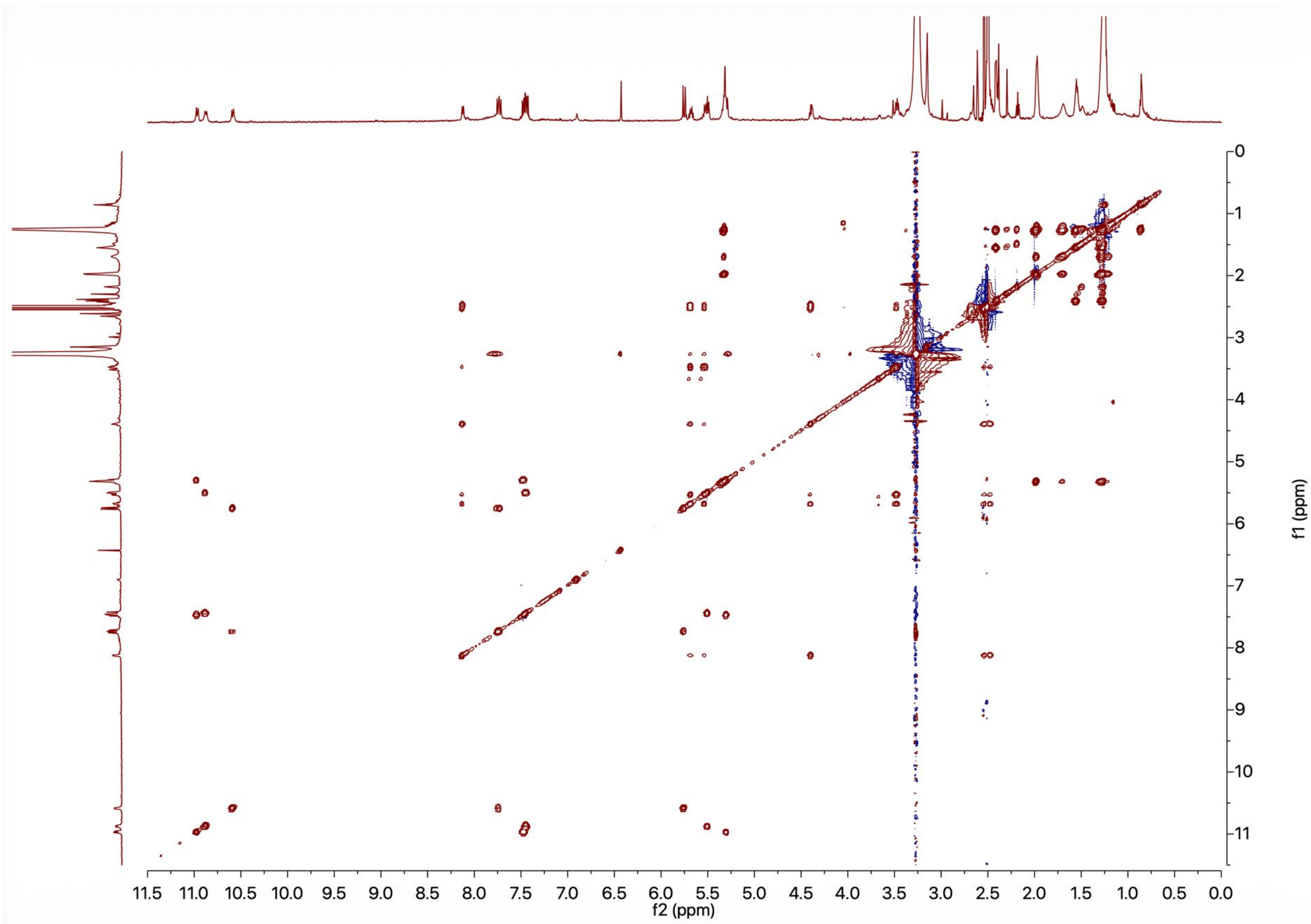

Figure S26. ^{1}H–^{1}H TOCSY Spectrum of AFC-BC11 obtained from LB medium, (DMSO-d_6, 600 MHz, 313K)

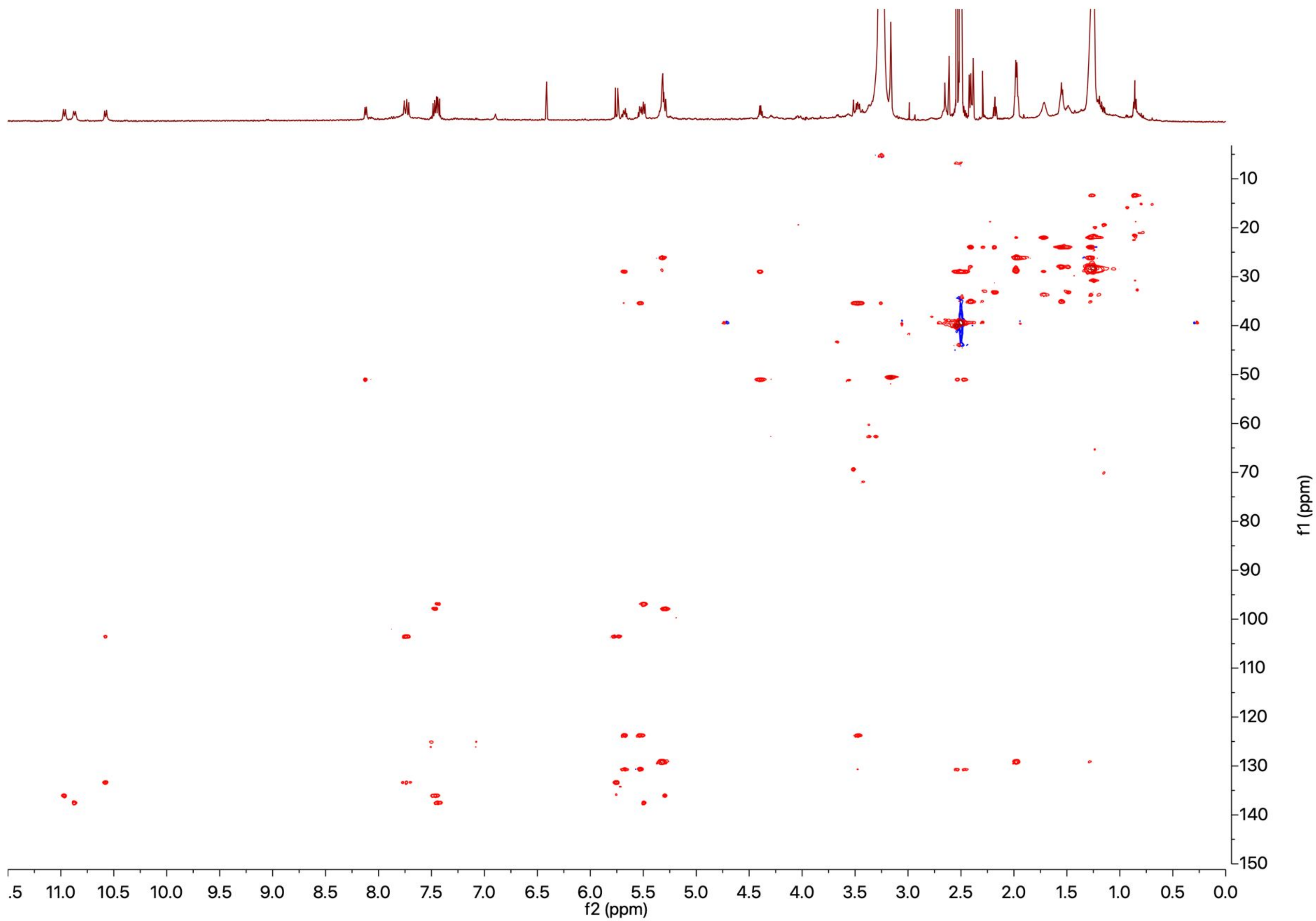

Figure S27. $^{1}H–^{13}C$ HSQC-TOCSY Spectrum (60 ms) of AFC-BC11 obtained from LB medium, (DMSO-d_6, 600 MHz, 313K)

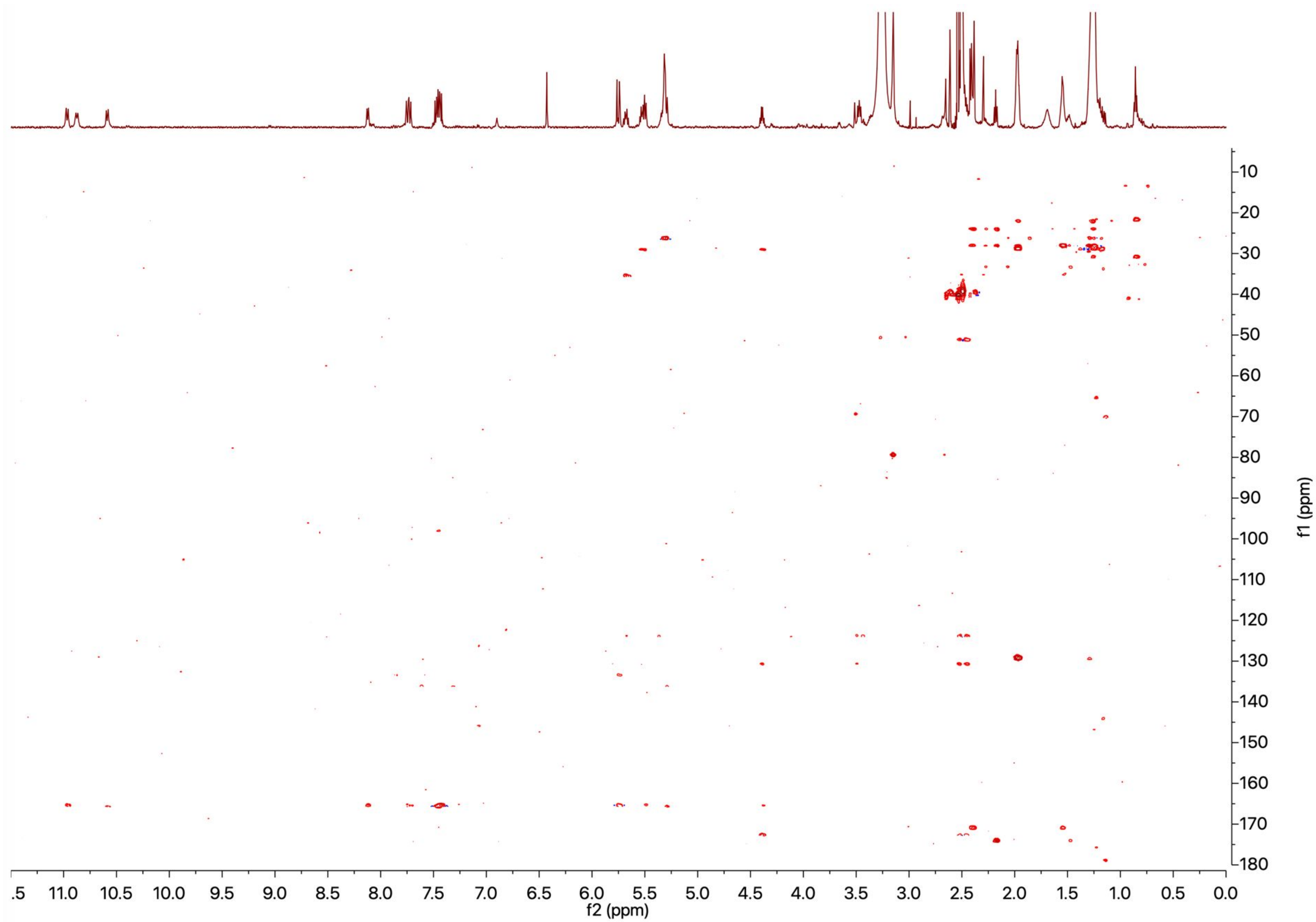

Figure S28. ^{1}H–^{13}C HMBC Spectrum of AFC-BC11 obtained from LB medium, (DMSO-d_6, 600 MHz, 313K)

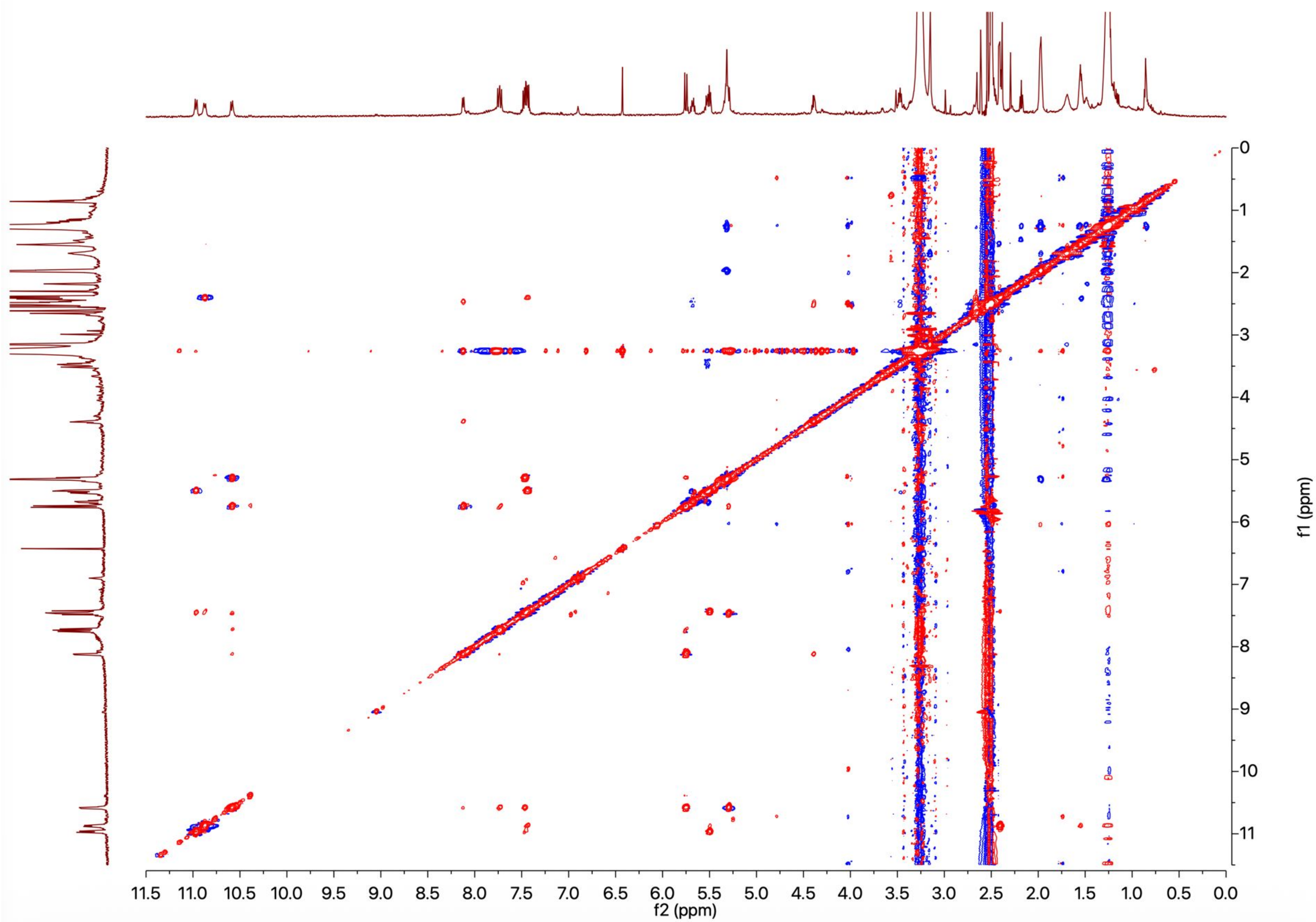

Figure S29. ^{1}H–^{1}H NOESY Spectrum of AFC-BC11 obtained from LB medium, (DMSO-d_6, 600 MHz, 313K)

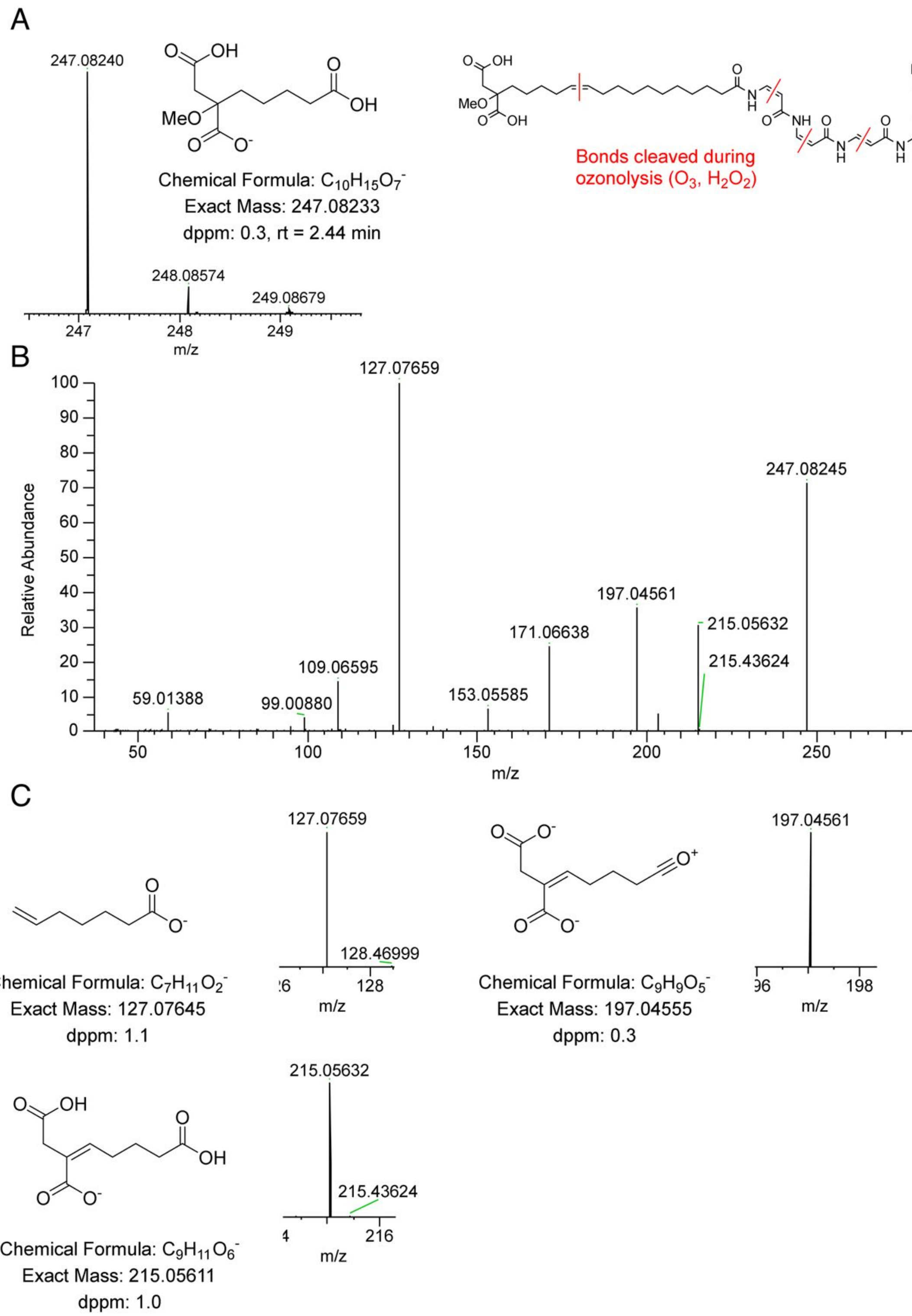

Figure S30. AFC-BC11 degradation product at 2.44 min after oxidation with ozone and oxidative workup of the compound produced by *B.* cenocepacia K56-2. (**A**) HR-MS spectrum, (**B**) HR-

MS/MS spectrum, and (**C**) proposed fragment structures with *m*/*z* extracted from HR-MS/MS spectrum. The results were obtained using Orbitrap Exploris 240 in negative mode.

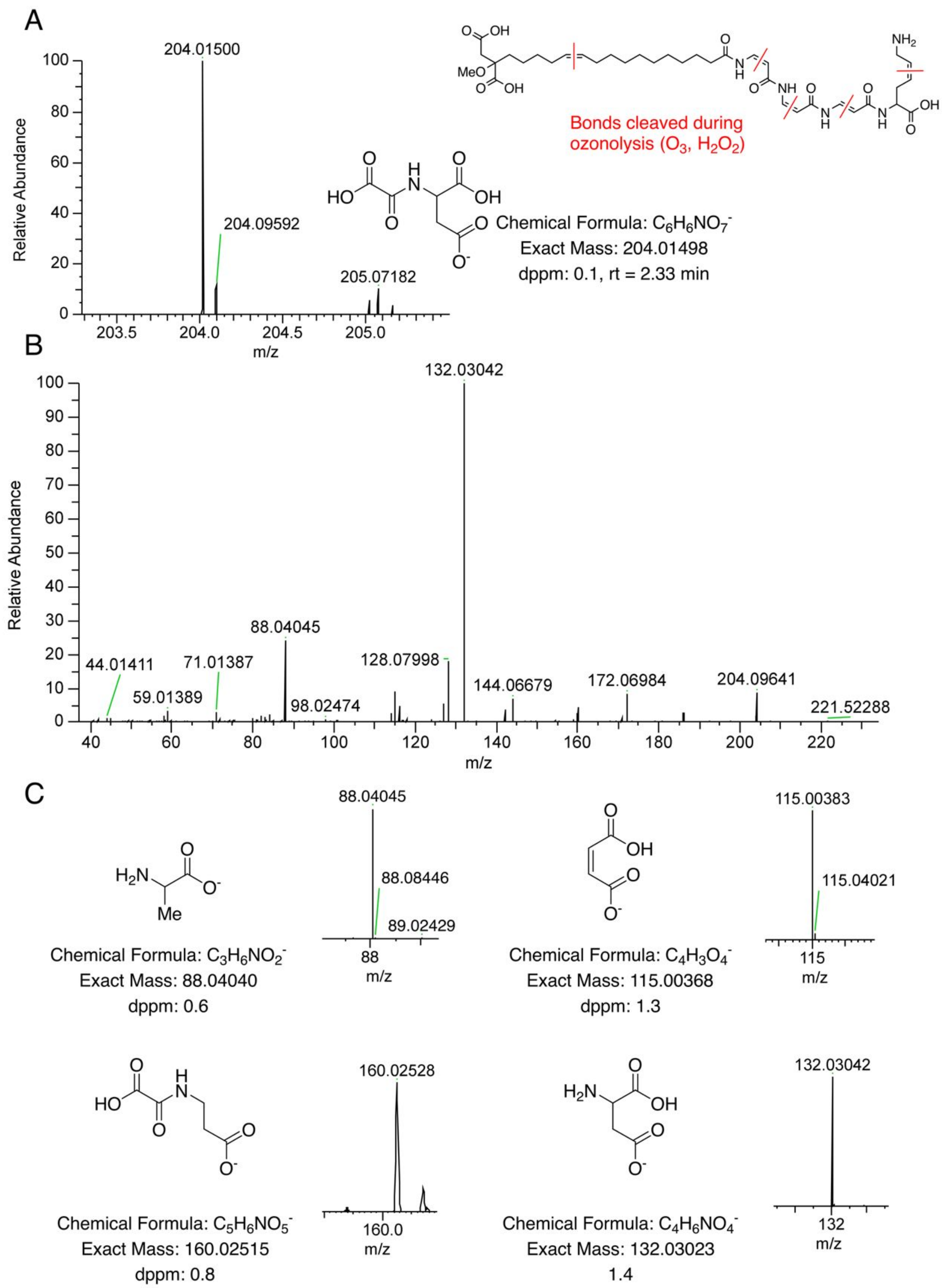

Figure S31. AFC-BC11 degradation product at 2.33 min after oxidation with ozone and oxidative workup of the compound produced by *B.* cenocepacia K56-2. (**A**) HR-MS spectrum, (**B**) HR-MS/MS spectrum, and (**C**) proposed fragment structures with *m*/*z* extracted from HR-MS/MS spectrum. The results were obtained using Orbitrap Exploris 240 in negative mode.

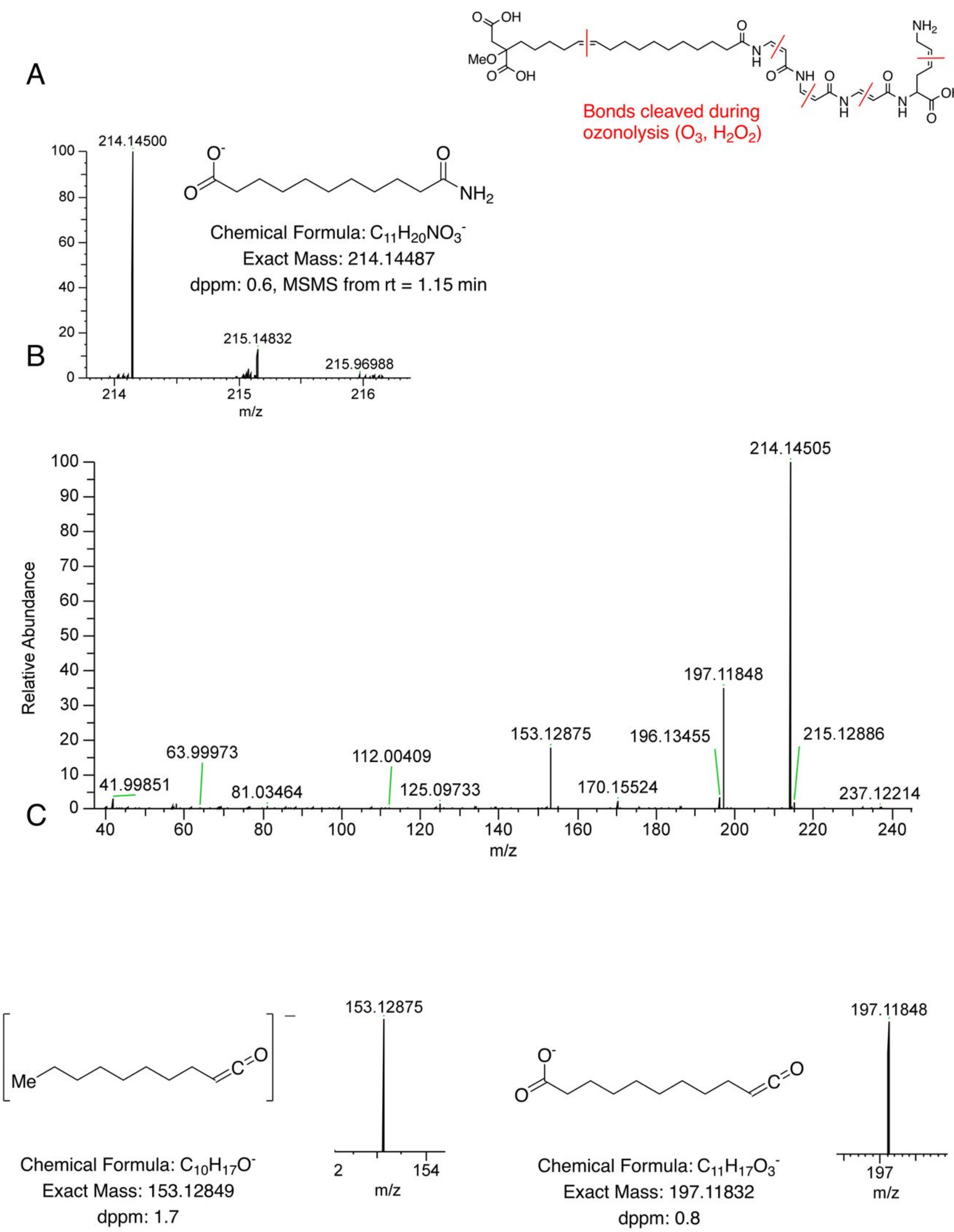

Figure S32. AFC-BC11 degradation product at 1.15 min after oxidation with ozone and oxidative workup of the compound produced by *B.* cenocepacia K56-2. (**A**) HR-MS spectrum, (**B**) HR-MS/MS spectrum, and (**C**) proposed fragment structures with *m/z* extracted from HR-MS/MS spectrum. The results were obtained using Orbitrap Exploris 240 in negative mode.

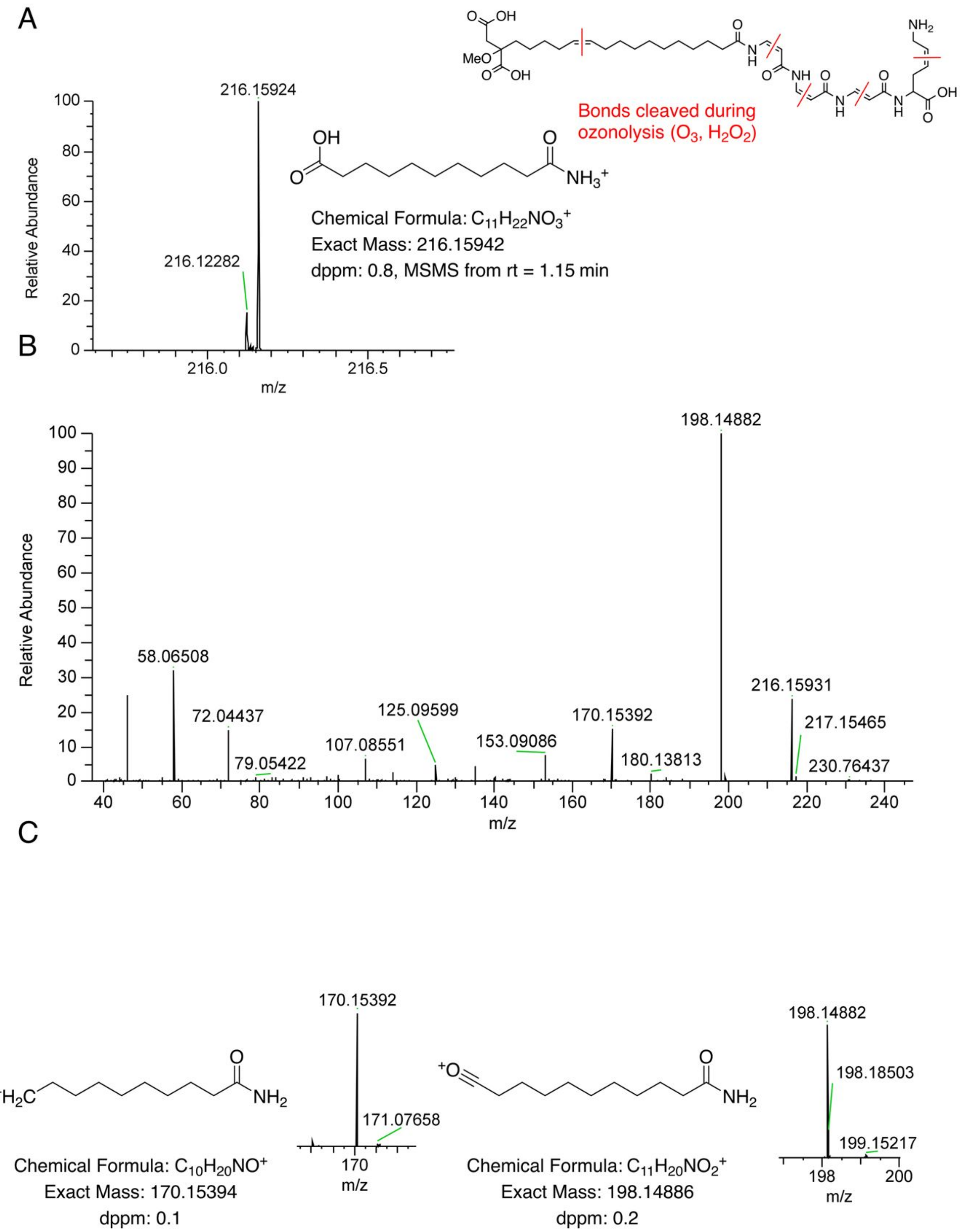

Figure S33. AFC-BC11 degradation product at 1.15 min after oxidation with ozone and oxidative workup of the compound produced by *B.* cenocepacia K56-2. (**A**) HR-MS spectrum, (**B**) HR-MS/MS spectrum, and (**C**) proposed fragment structures with *m/z* extracted from HR-MS/MS spectrum. The results were obtained using Orbitrap Exploris 240 in positive mode.

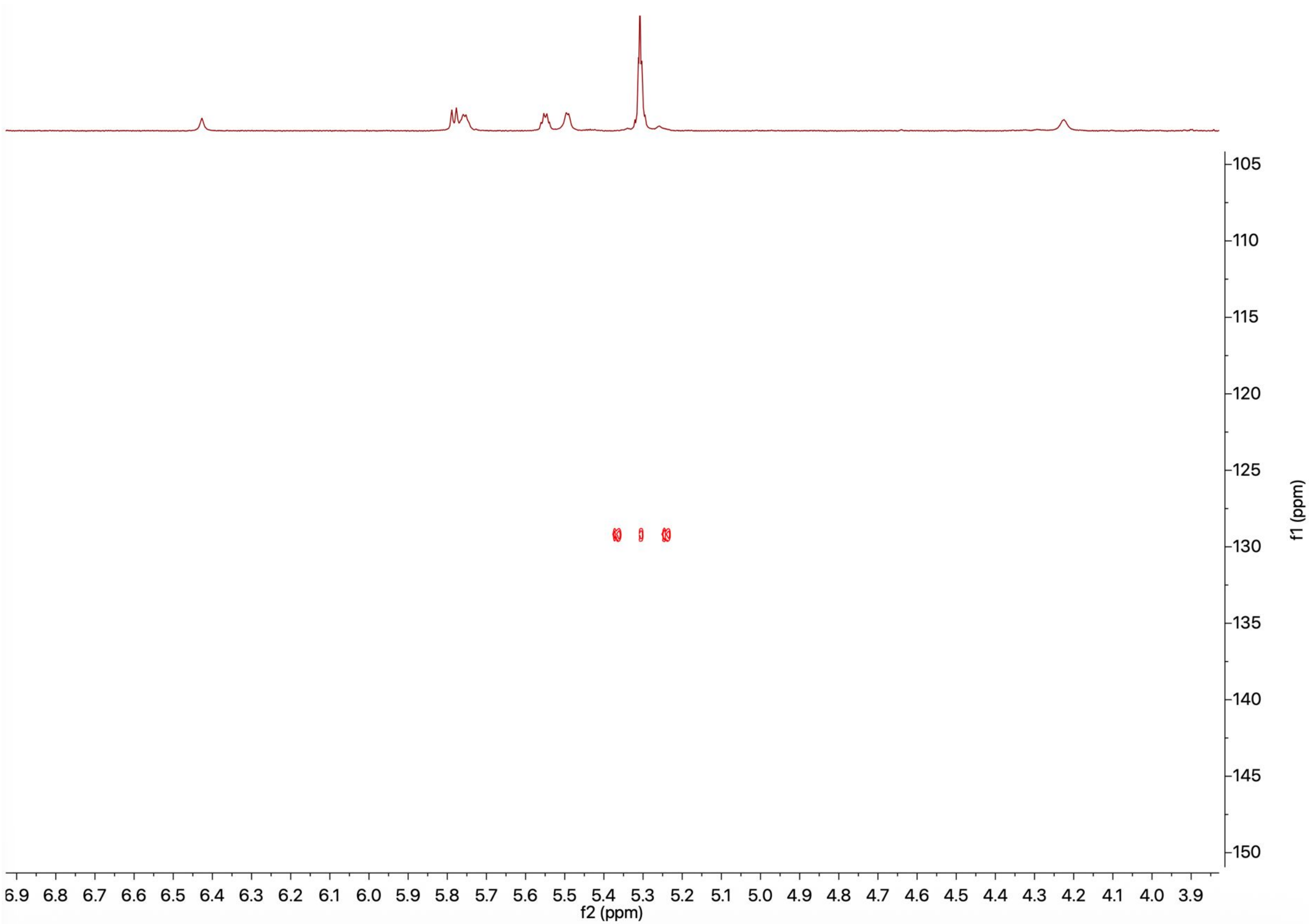

6.9 6.8 6.7 6.6 6.5 6.4 6.3 6.2 6.1 6.0 5.9 5.8 5.7 5.6 5.5 5.4 5.3 5.2 5.1 5.0 4.9 4.8 4.7 4.6 4.5 4.4 4.3 4.2 4.1 4.0 3.9
f2 (ppm)
105 110 115 120 125 130 135 140 145 150
f1 (ppm)

Figure S34. ^{1}H–^{13}C HSQC Spectrum of AFC-BC11 acquired without ^{13}C decoupling and with selective ^{1}H decoupling at 1.96 ppm during acquisition, (DMSO-d_6, 1.2 GHz, 313K).

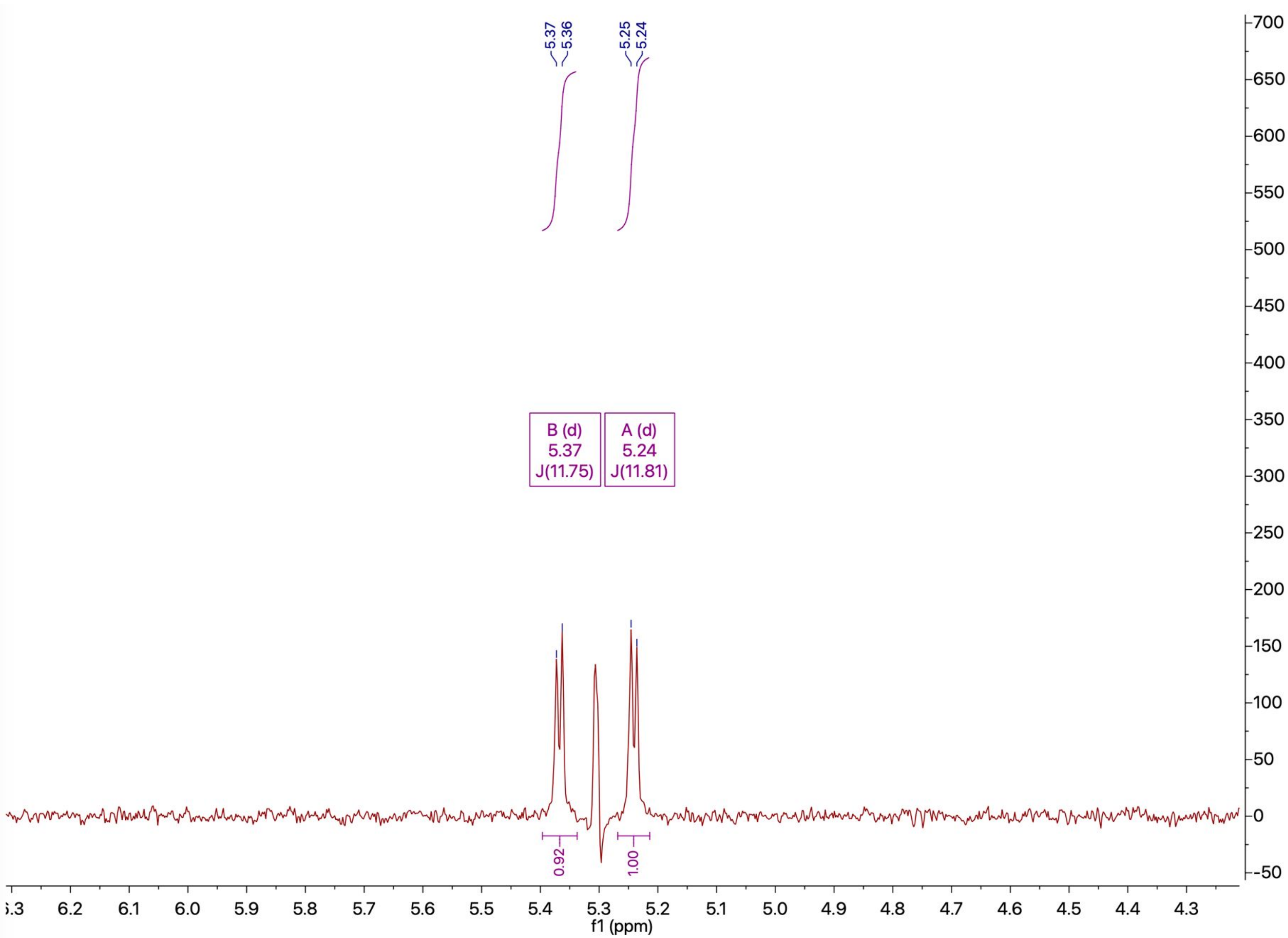
5.37
5.36
5.25
5.24
B (d)
5.37
J(11.75)
A (d)
5.24
J(11.81)
0.92
1.00
f1 (ppm)

Figure S35. Extracted trace at 129.2 ppm from the ^{1}H–^{13}C HSQC spectrum of AFC-BC11 acquired without ^{13}C decoupling and with selective ^{1}H decoupling at 1.96 ppm during acquisition, (DMSO-d_6, 1.2 GHz, 313K).

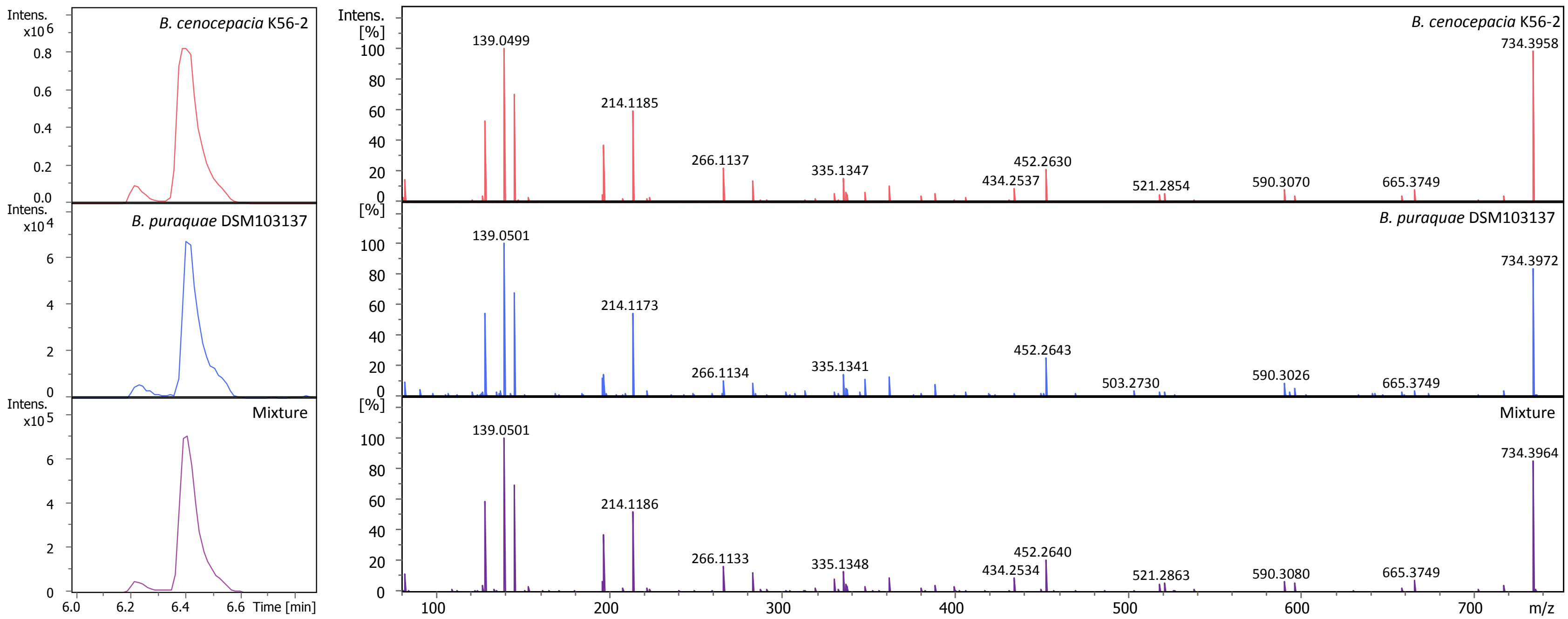

Figure S36. UHPLC-HR-MS/MS comparison of AFC-BC11 from *B. cenocepacia* K56-2 and *B. puraquae* DSM103137 grown in PDB.

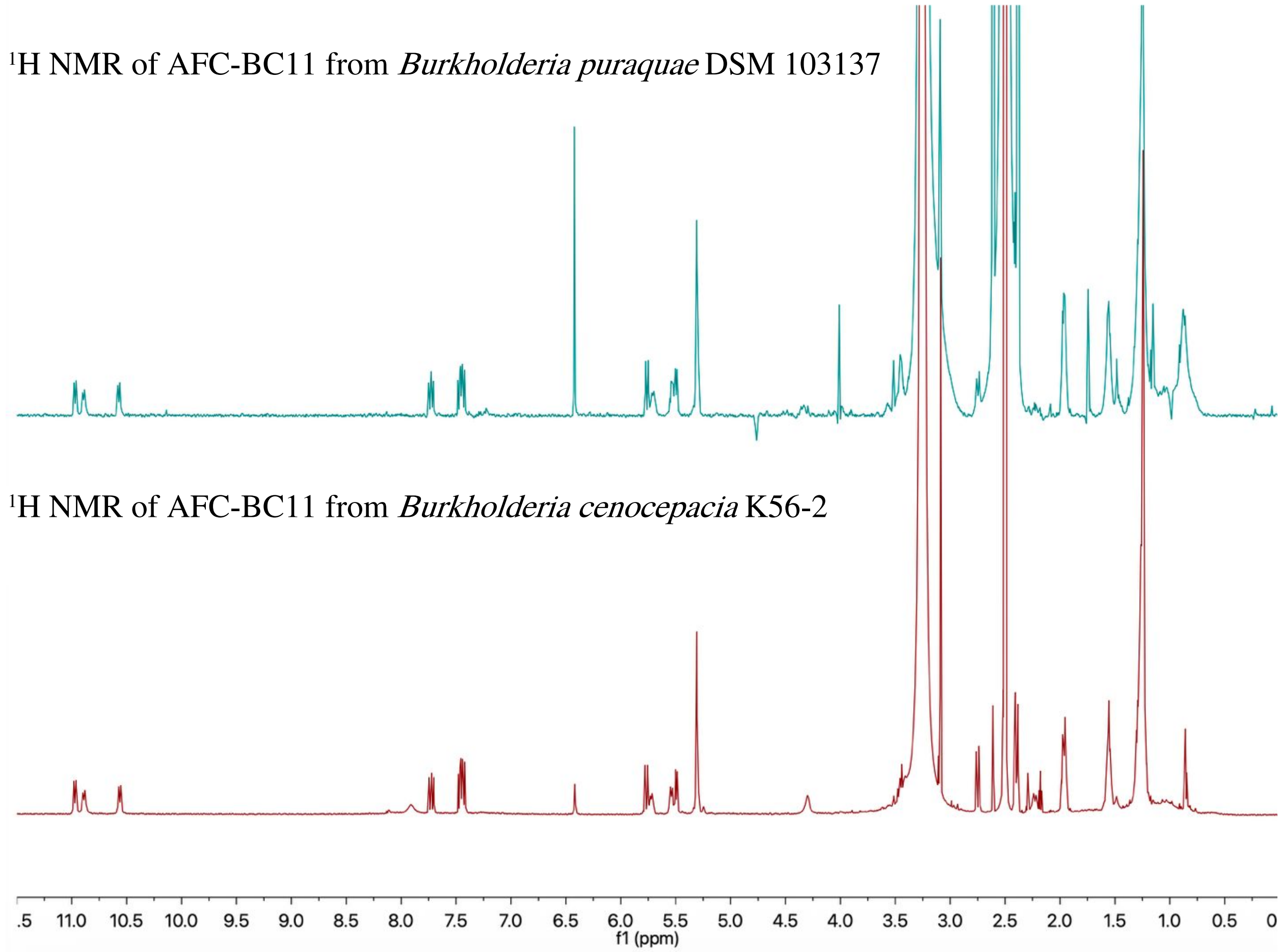

Figure S37. Comparison of the ^{1}H NMR spectra of AFC-BC11 extracted from *Burkholderia puraquae* DSM 103137 (top) and *Burkholderia cenocepacia* K56-2 (bottom). Spectra were acquired in DMSO-d_6 on a 600 MHz spectrometer at 313K.

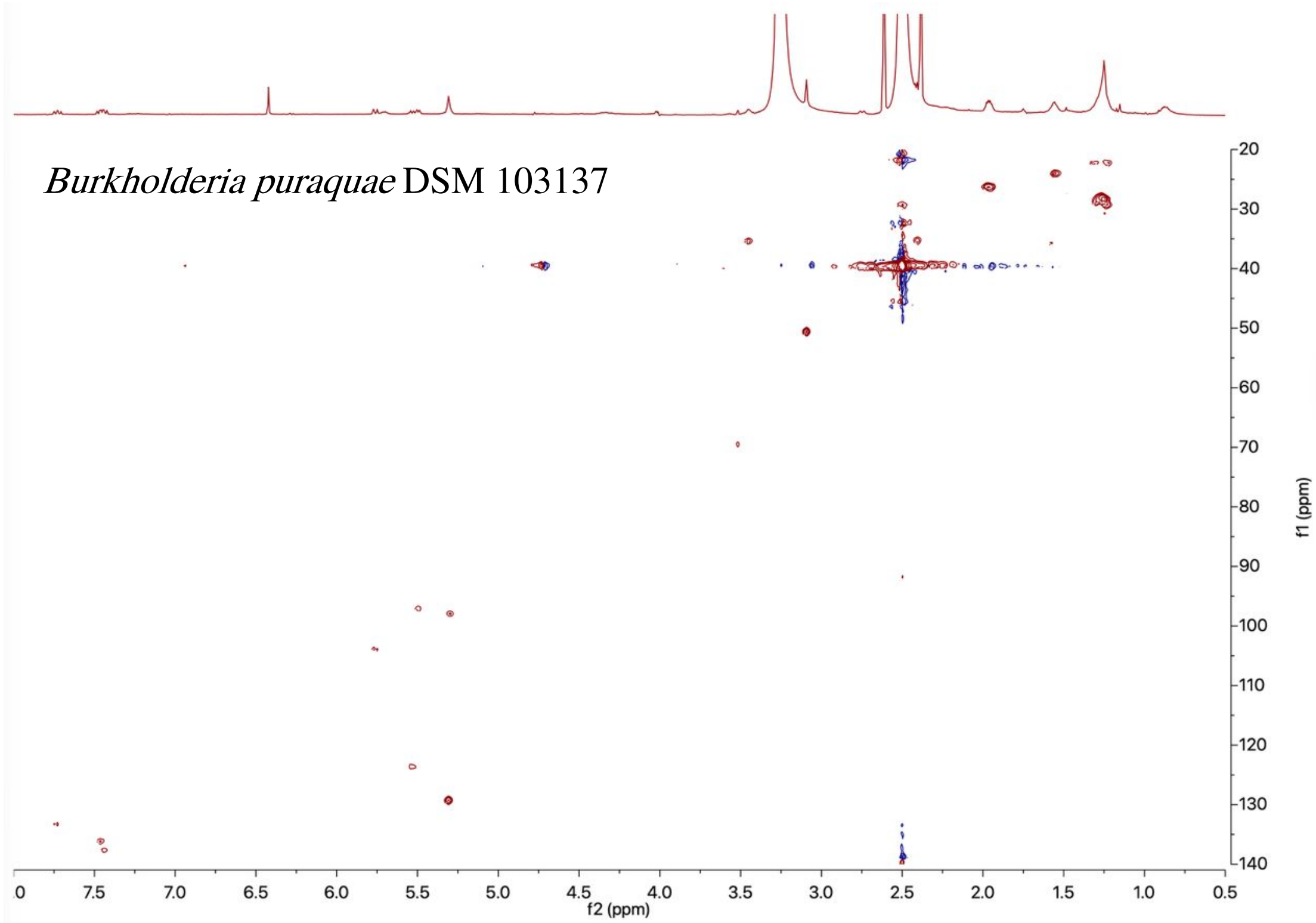

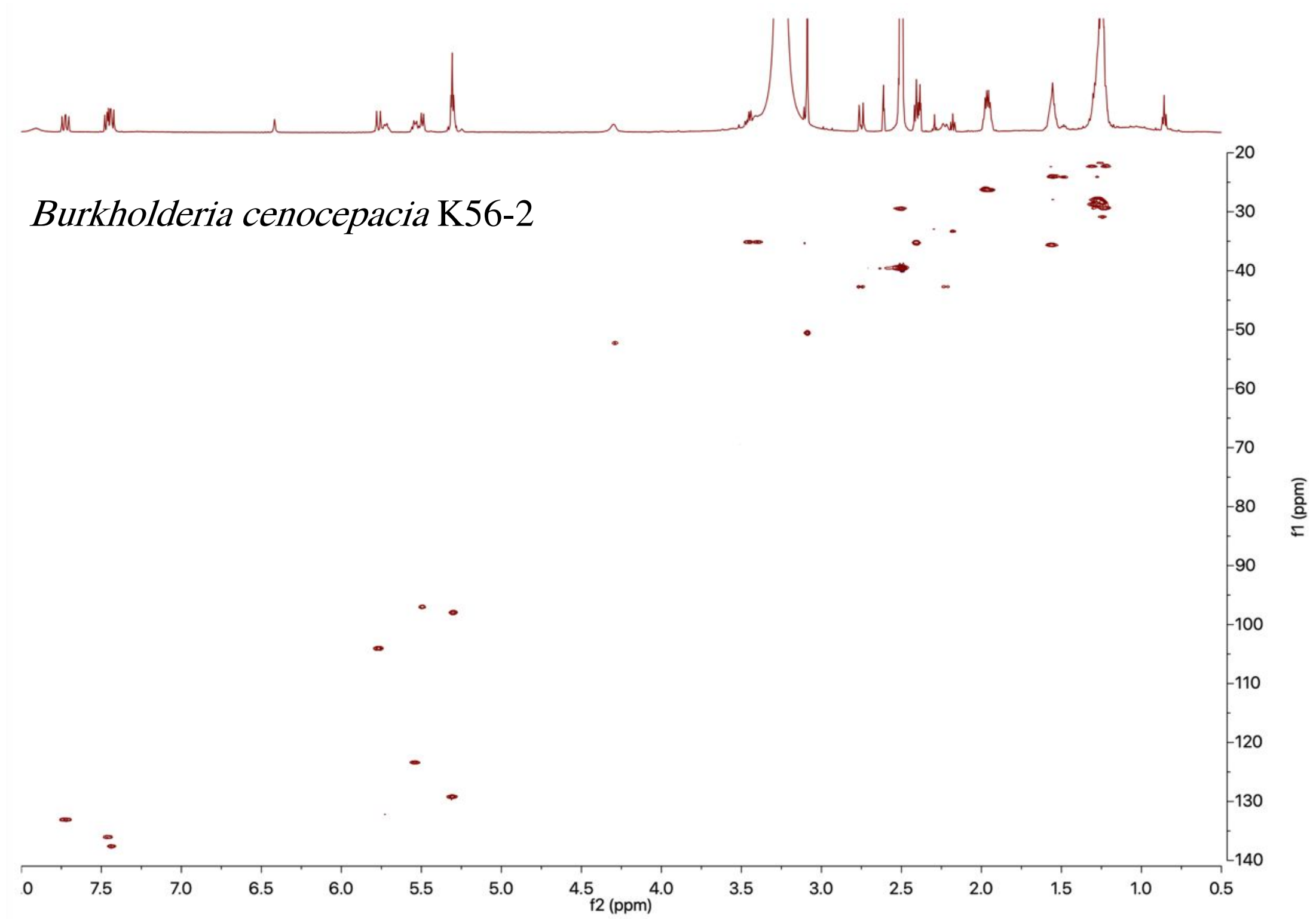

Figure S38. Comparison of the ¹H–¹³C HSQC spectra of AFC-BC11 extracted from *Burkholderia puraquae* DSM 103137 (top) and *Burkholderia cenocepacia* K56-2 (bottom). Spectra were acquired in DMSO-d_6 on a 600 MHz spectrometer at 313K.

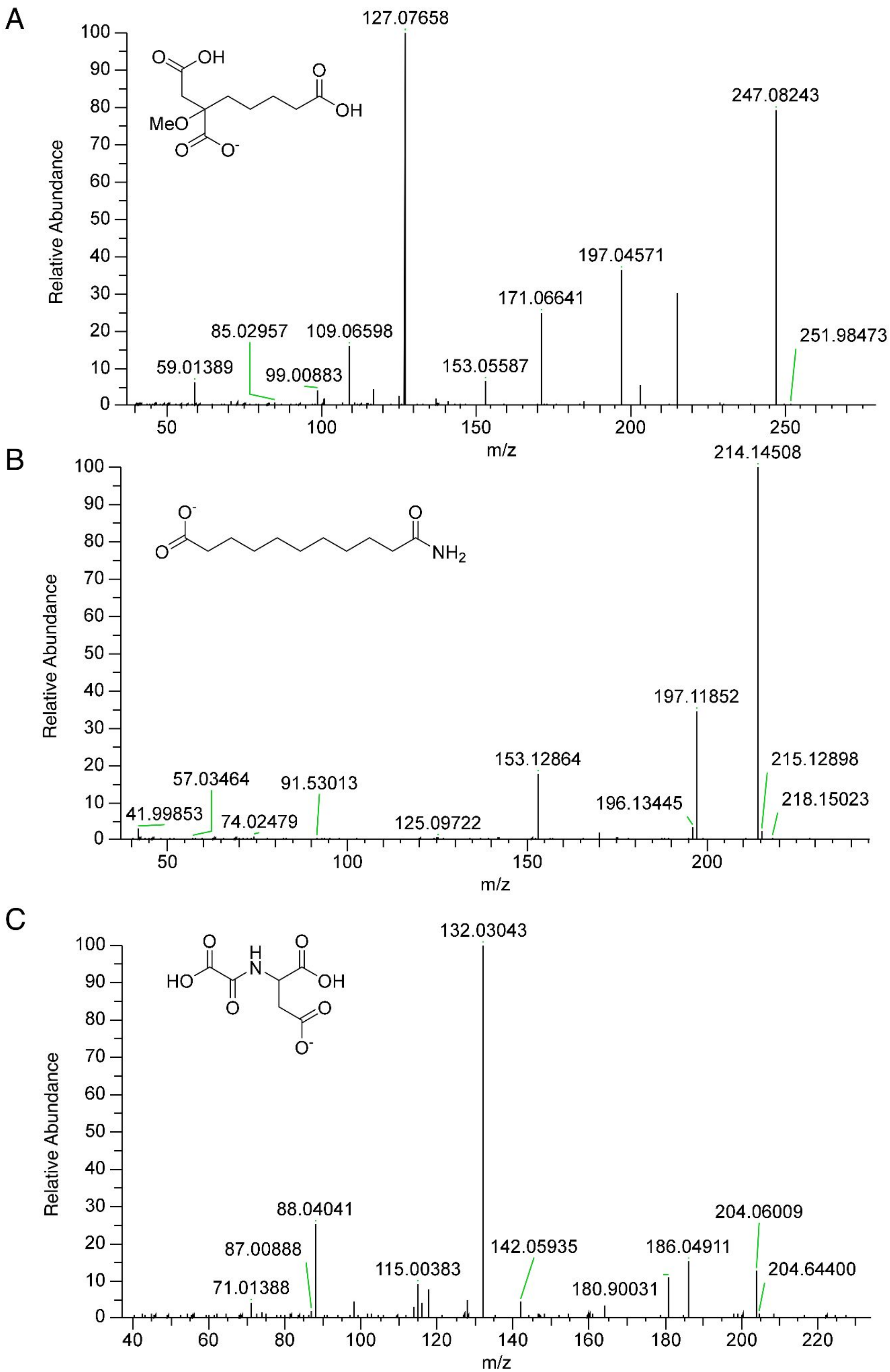
A
127.07658
247.08243
197.04571
171.06641
85.02957
109.06598
251.98473
59.01389
99.00883
153.05587
OH
MeO
Relative Abundance
m/z
B
214.14508
197.11852
153.12864
215.12898
57.03464
91.53013
218.15023
196.13445
41.99853
74.02479
125.09722
NH_2
Relative Abundance
m/z
C
132.03043
88.04041
204.06009
87.00888
142.05935
186.04911
115.00383
204.64400
71.01388
180.90031
HO
OH
Relative Abundance
m/z

D

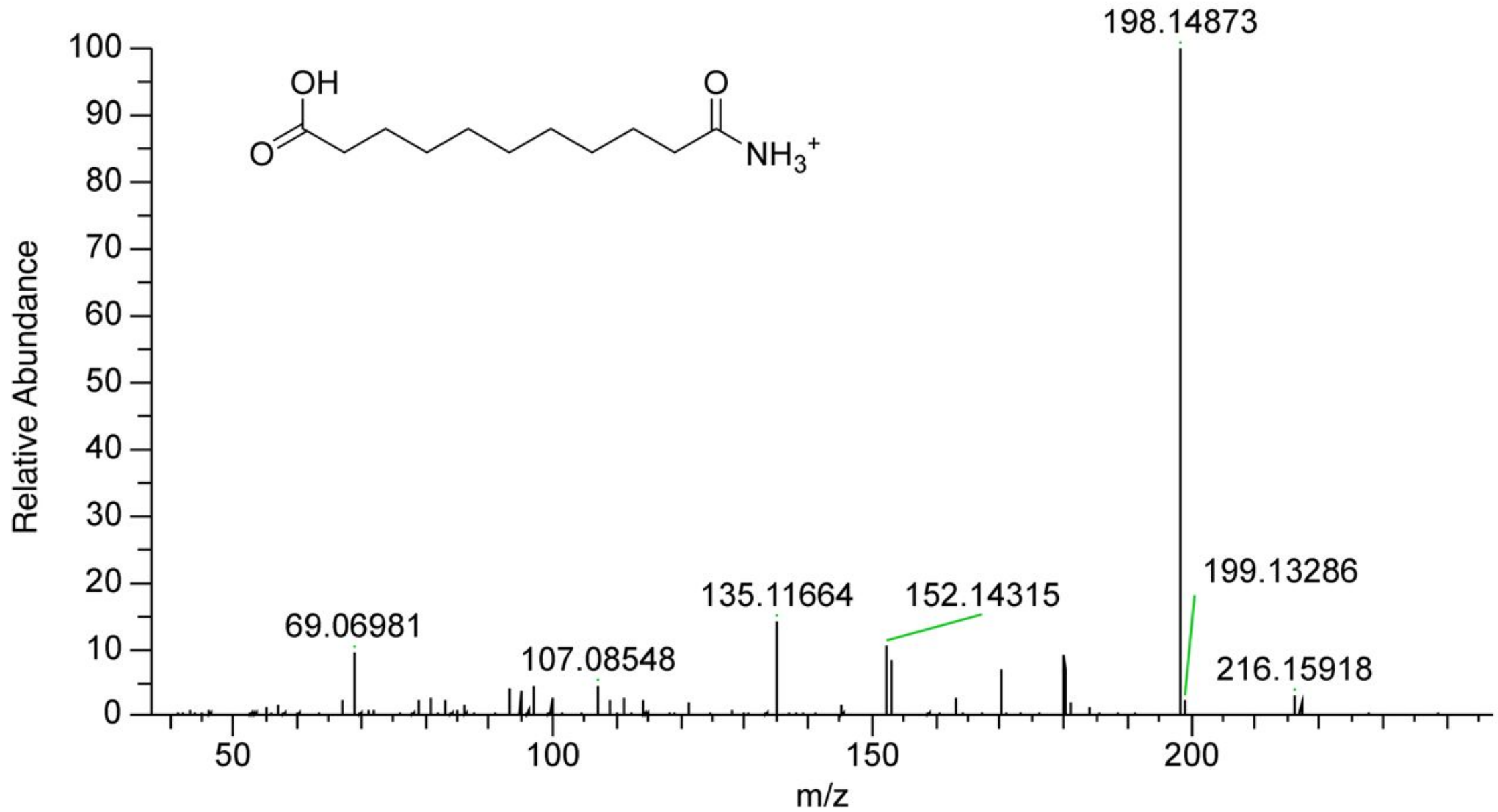

Figure S39. AFC-BC11 degradation products after oxidation with ozone and oxidative workup of the compound produced by *B. puraquae*. (**A**-**D**) HR-MS/MS spectra and proposed structures of compounds previously identified from the ozone and oxidative workup of AFC-BC11 (**1**) produced by *B.* cenocepacia K56-2. The results were obtained using Orbitrap Exploris 240 in negative (**A**-**C**) and positive mode (**D**).

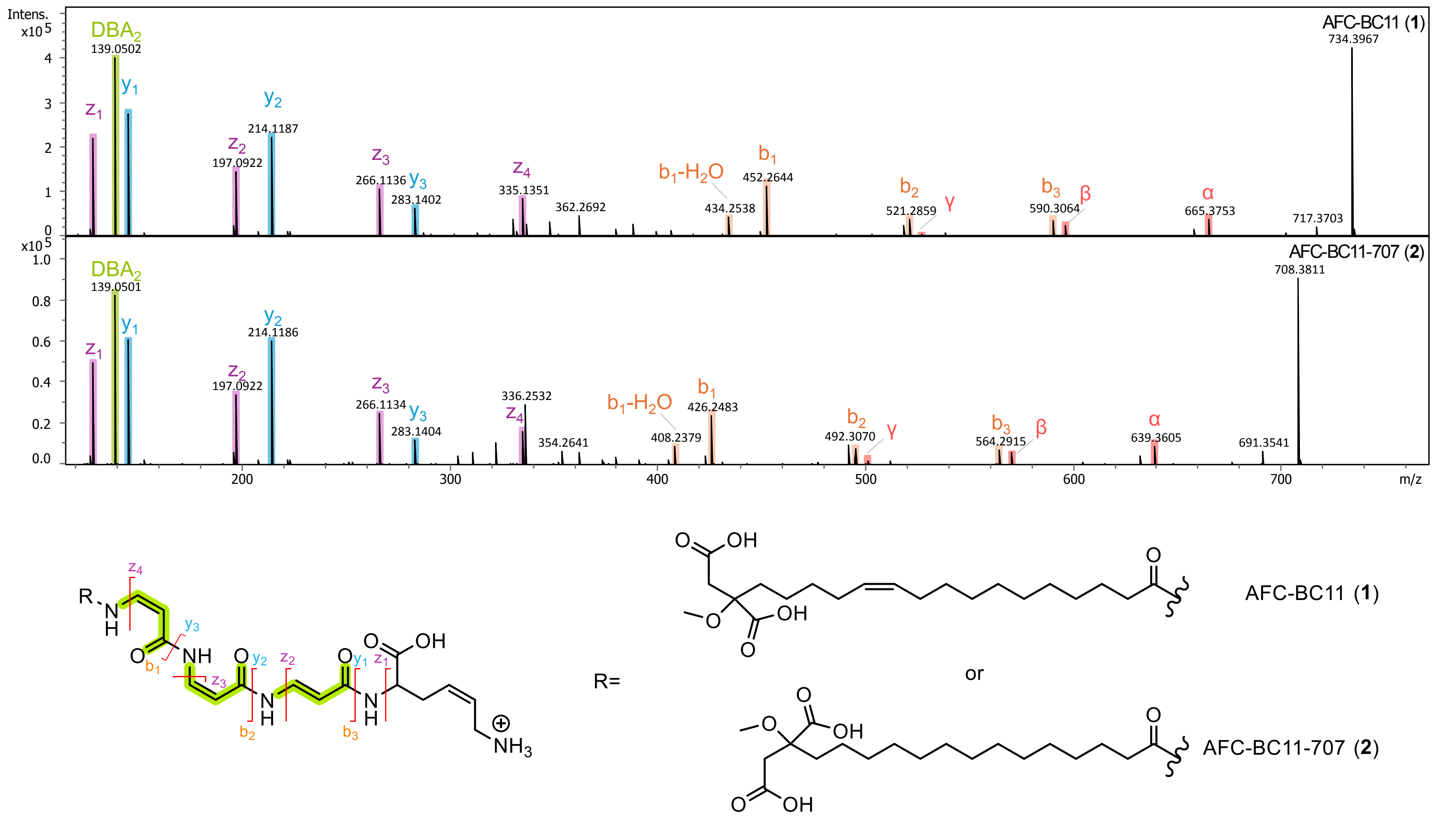

Figure S40. HR-MS/MS fragmentation pattern of MMFA-AFCs AFC-BC11 (**1**) and AFC-BC11-707 (**2**).

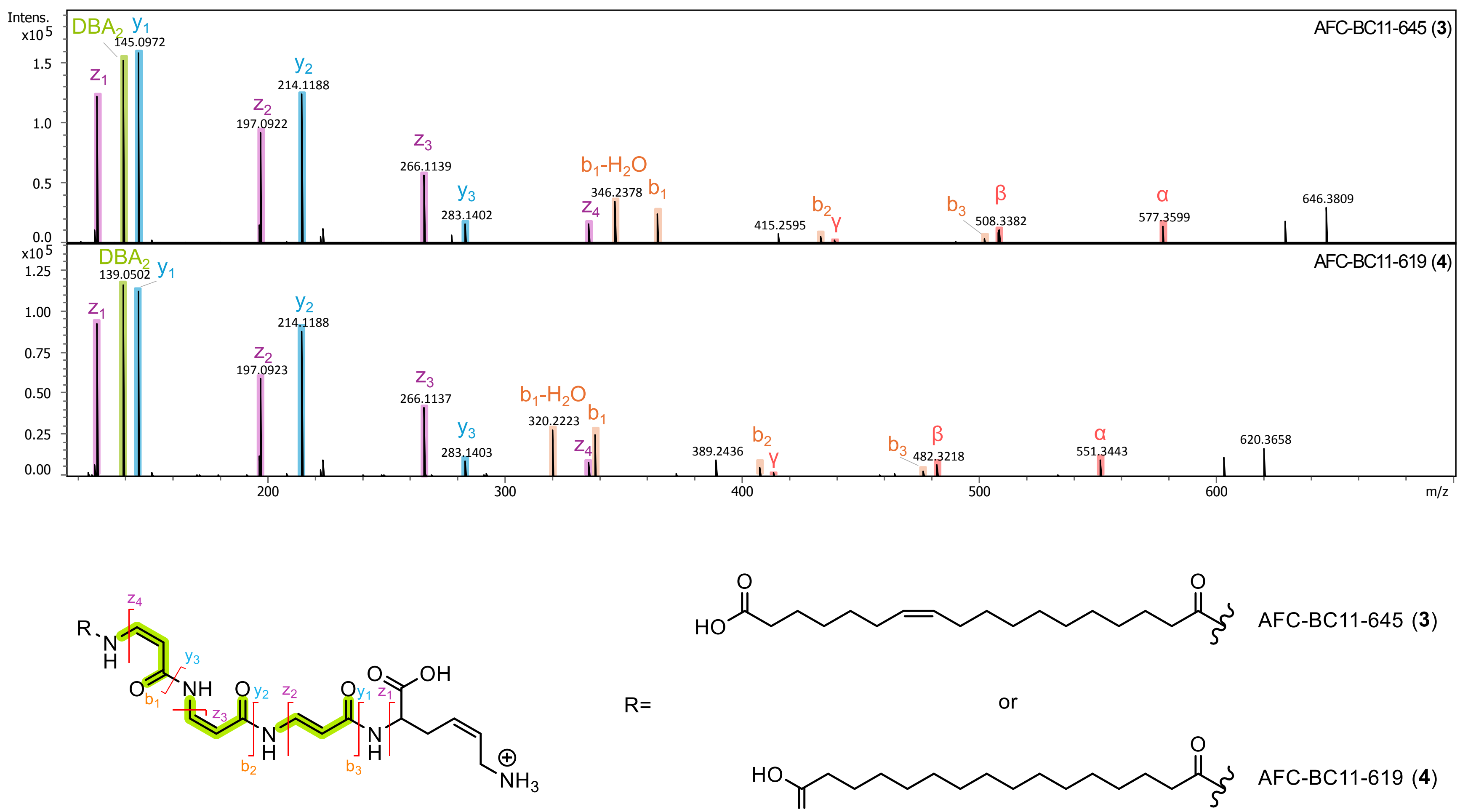

Figure S41. HR-MS/MS fragmentation pattern of ω-CFA-AFCs AFC-BC11-645 (**3**) and AFC-BC11-619 (**4**).

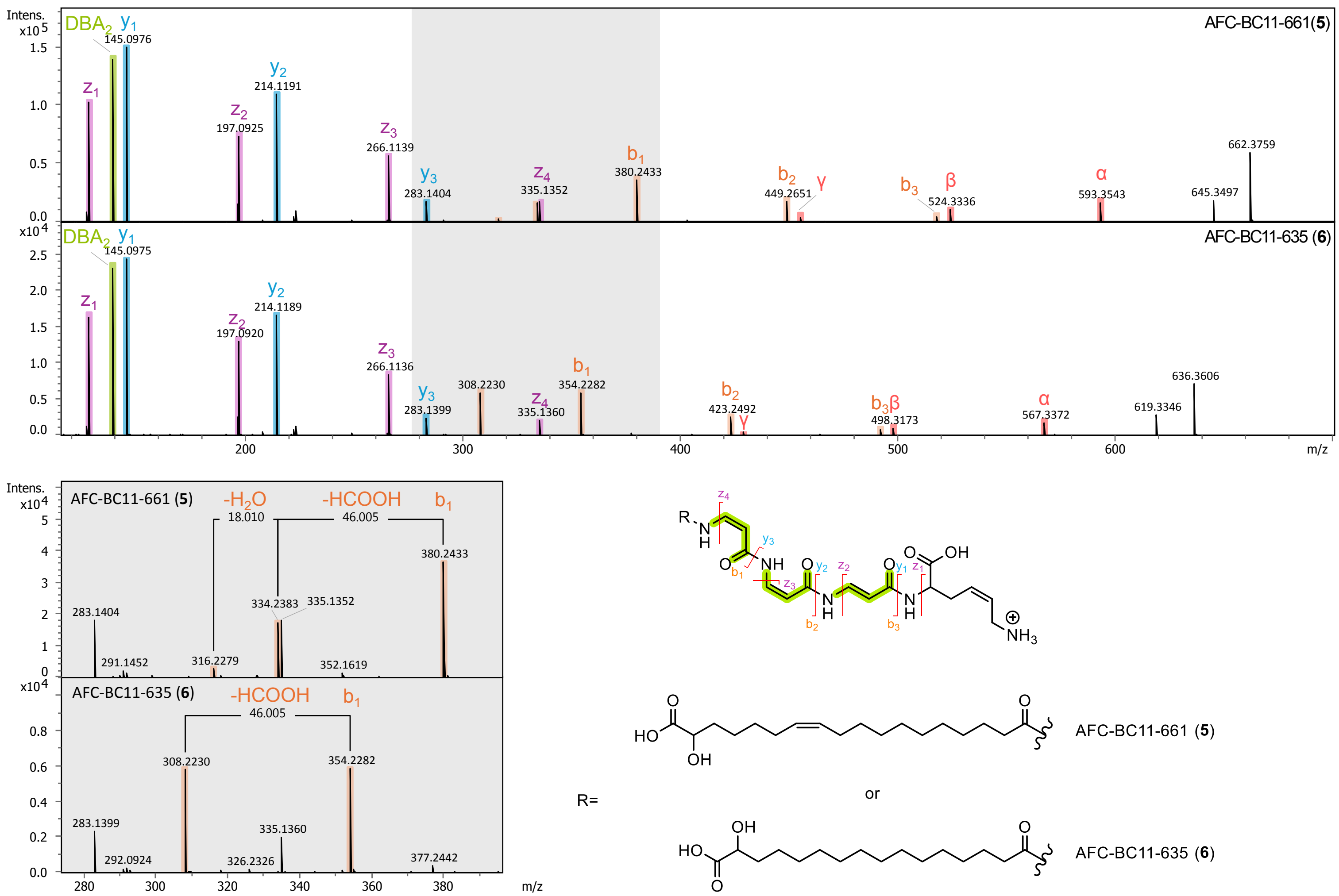

Intens. x10^5
DBA2
y1
145.0976
z1
y2
214.1191
z2
197.0925
z3
266.1139
y3
283.1404
z4
335.1352
b1
380.2433
b2
449.2651
γ
b3
β
524.3336
α
593.3543
645.3497
662.3759
AFC-BC11-661(5)
x10^4
DBA2
y1
145.0975
z1
y2
214.1189
z2
197.0920
z3
266.1136
y3
283.1399
308.2230
z4
335.1360
b1
354.2282
b2
423.2492
γ
b3β
498.3173
α
567.3372
619.3346
636.3606
AFC-BC11-635 (6)
m/z
Intens. x10^4
AFC-BC11-661 (5)
-H2O
18.010
-HCOOH
46.005
b1
380.2433
283.1404
291.1452
316.2279
334.2383
335.1352
352.1619
x10^4
AFC-BC11-635 (6)
-HCOOH
46.005
b1
308.2230
354.2282
283.1399
292.0924
335.1360
326.2326
377.2442
m/z
R
NH
O
OH
NH3
AFC-BC11-661 (5)
HO
OH
or
R=
AFC-BC11-635 (6)

Figure S42. HR-MS/MS fragmentation pattern of α-hydroxy ω-CFA AFCs AFC-BC11-661 (**5**) and AFC-BC11-635 (**6**). The enlarged spectrum highlights the sequential loss of terminal carboxy and hydroxy groups from the b_1 ion (shaded in grey).

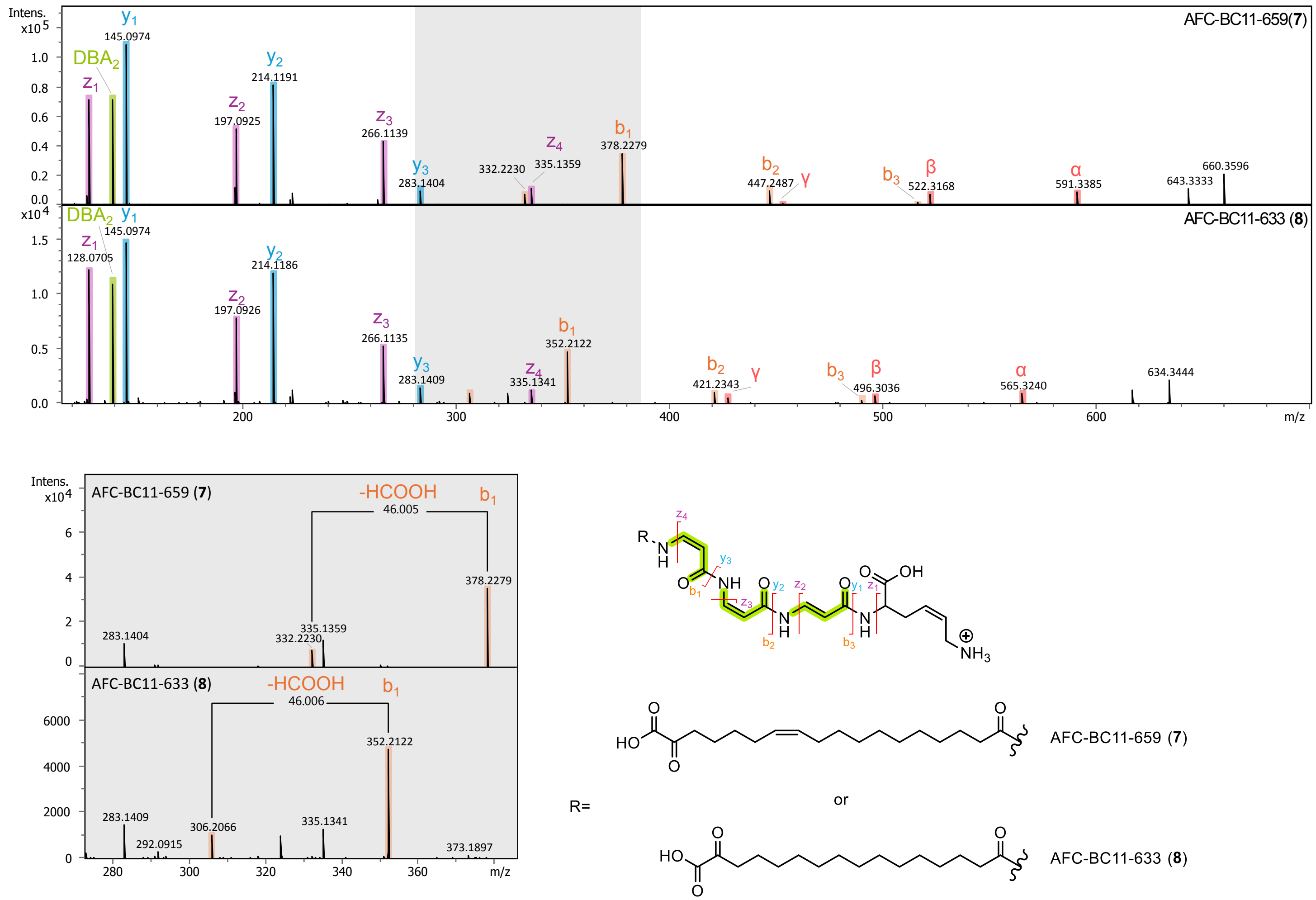

AFC-BC11-659(7)
AFC-BC11-633 (8)
Intens.
m/z
-HCOOH
46.005
46.006
R=
or
AFC-BC11-659 (7)
AFC-BC11-633 (8)

Figure S43. HR-MS/MS fragmentation pattern of α-oxo ω-CFA AFC-BC11-659 (**7**) and AFC-BC11-633 (**8**). The enlarged spectrum highlights the loss of terminal carboxy group from the b_1 ion (shaded in grey).

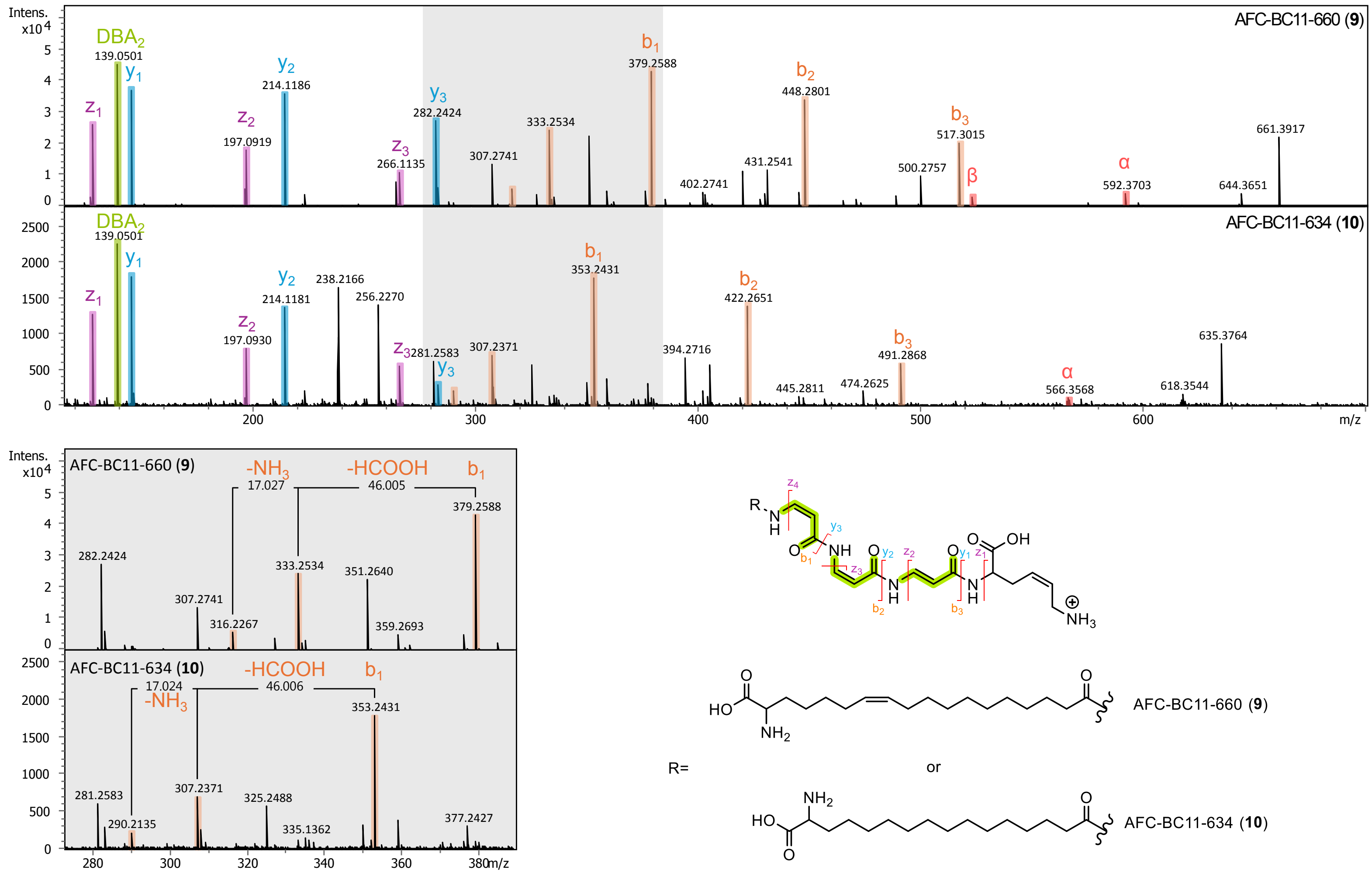

Figure S44. HR-MS/MS fragmentation pattern of α-amino ω-CFA AFCs AFC-BC11-660 (**9**) and AFC-BC11-634 (**10**). The enlarged spectrum highlights the sequential loss of terminal carboxy and amino groups from the b_1 ion.

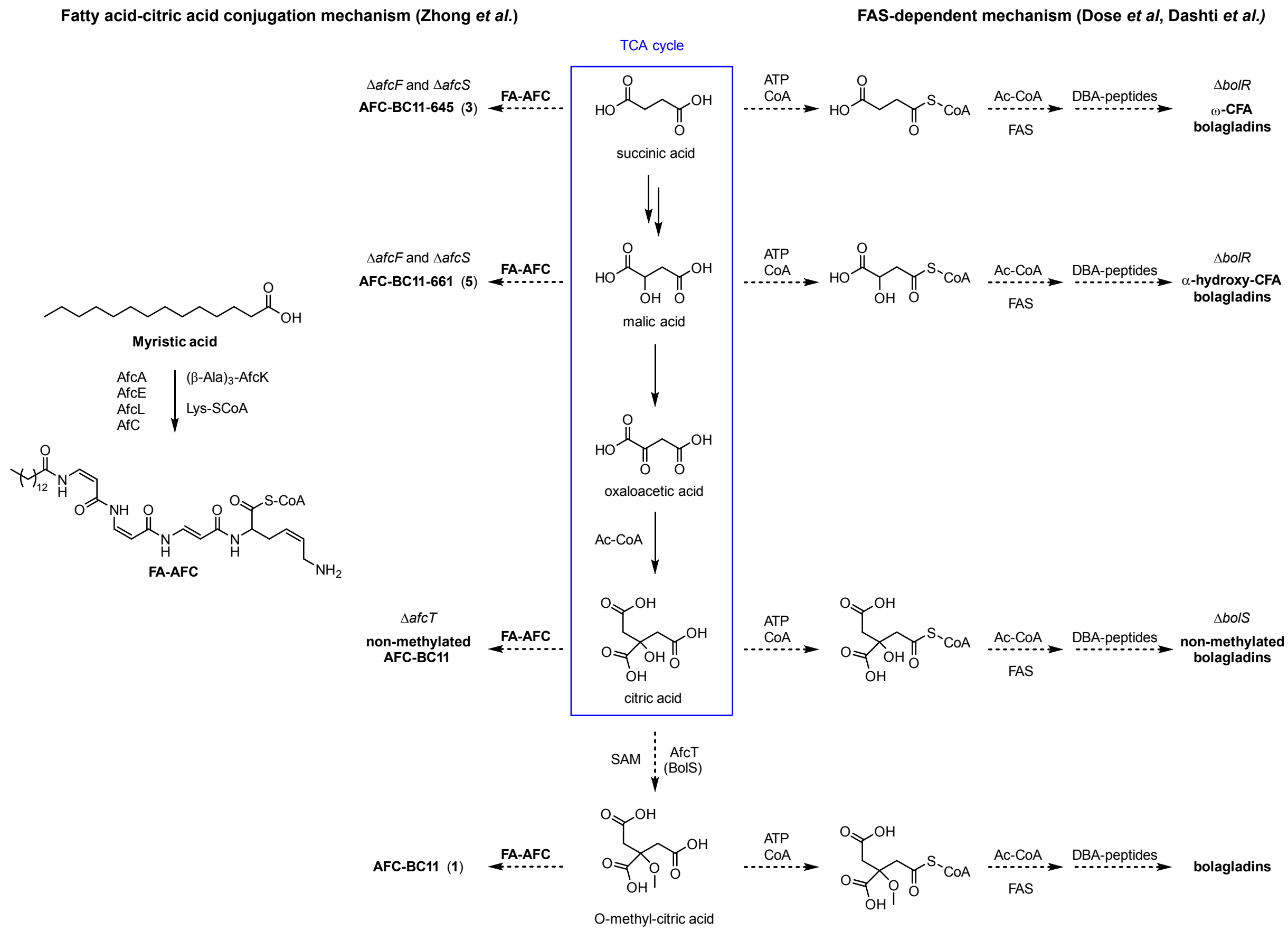

Figure S45. Previously proposed biosynthetic pathways of AFC-BC11 (**1**) and bolagladins. Based on earlier studies, AFCs biosynthesis may proceed either via fatty acid-citric acid conjugation[6] (left) or through a FAS-dependent pathway (right) as proposed for bolagladin biosynthesis.[28,30] Reactions shared with the TCA cycle are shown in blue, and proposed reactions are represented with dashed arrows.

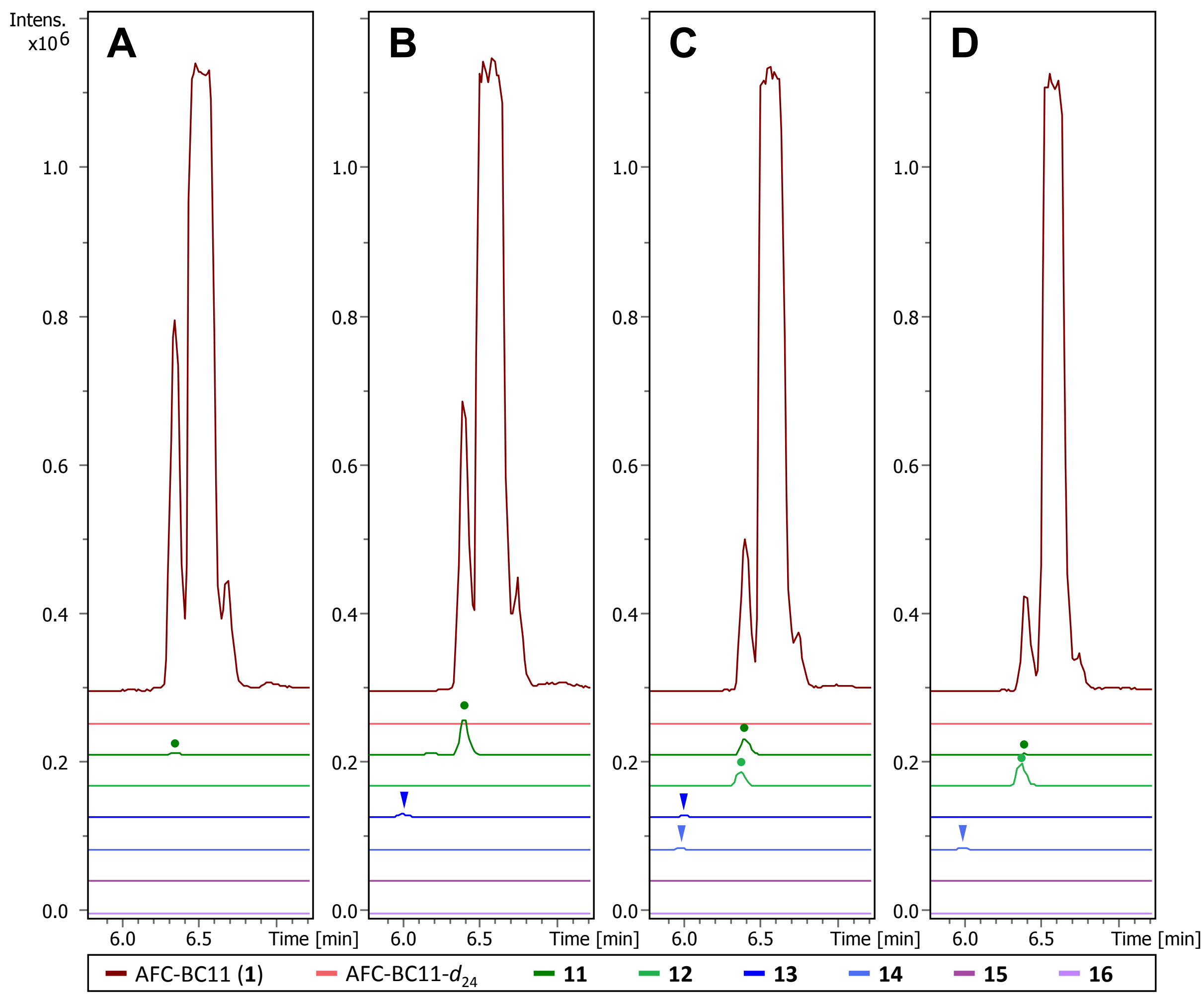

Figure S46. Extracted ion chromatograph of AFCs production following supplementation with myristic acids. PDB cultures of wild-type K56-2 were treated with (**A**) DMSO, (**B**) myristic acid, (**C**) myristic acid:myristic acid-d_{27}=1:1, and (**D**) myristic acid-d_{27}. Peaks marked with dots indicate accumulated ω-CFA myristic acid-AFC intermediates (**11** and **12**; green lines), while arrows denote minor unfavored final MMFA products (**13** and **14**; blue lines). No α-hydroxy ω-CFA myristic acid-AFCs were detected (**15** and **16**; purple lines).

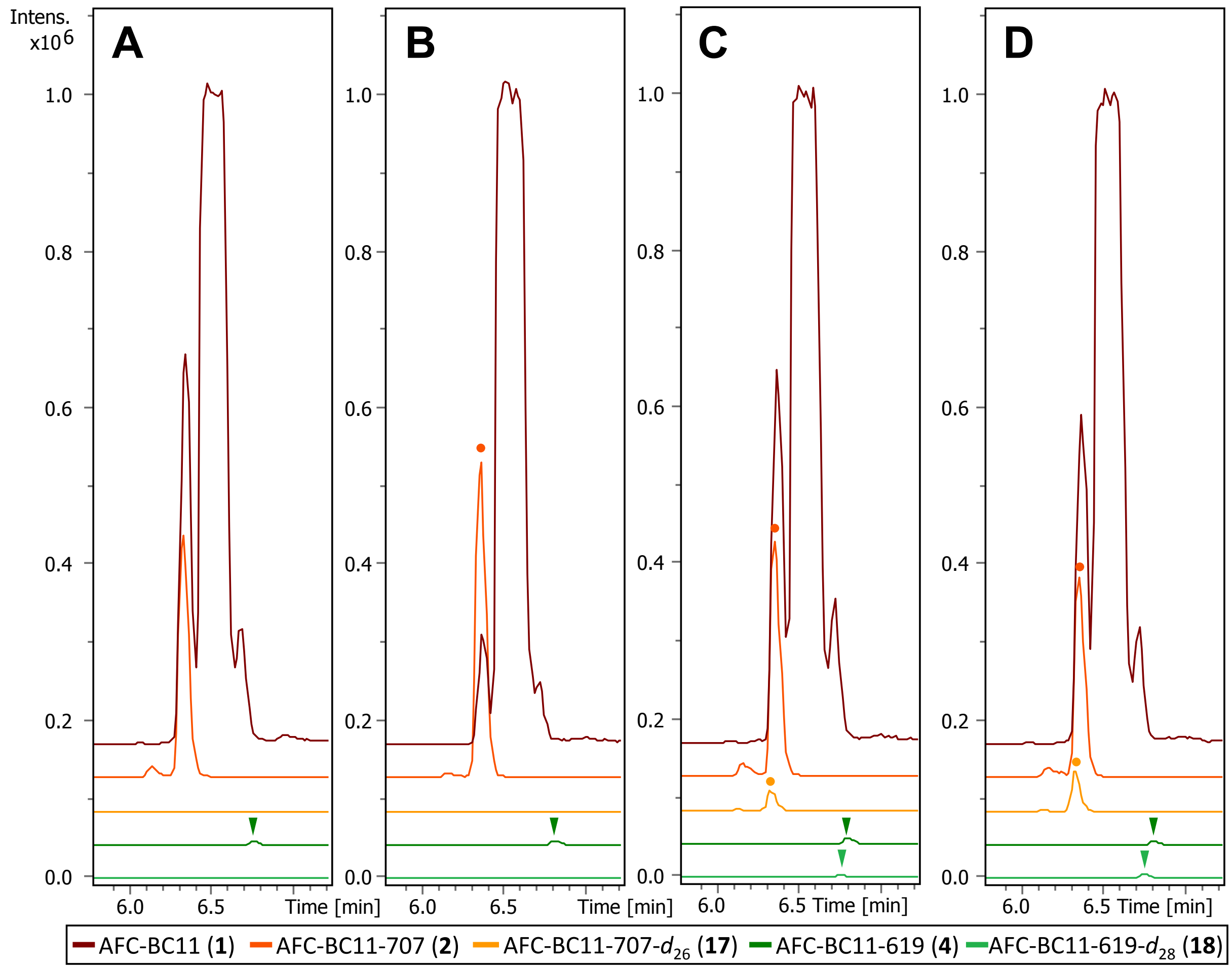

Figure S47. Extracted ion chromatograph of AFCs production following supplementation with palmitic acids. PDB cultures of wild-type K56-2 were treated with (**A**) DMSO, (**B**) palmitic acid, (**C**) palmitic acid:palmitic acid-d_{31}=1:1, and (**D**) palmitic acid-d_{31}. Peaks marked with dots indicate MMFA palmitic acid-AFCs (**2** and **17**; orange lines), while arrows denote minor ω-CFA palmitic acid-AFC derivatives (**4** and **18**; green lines).

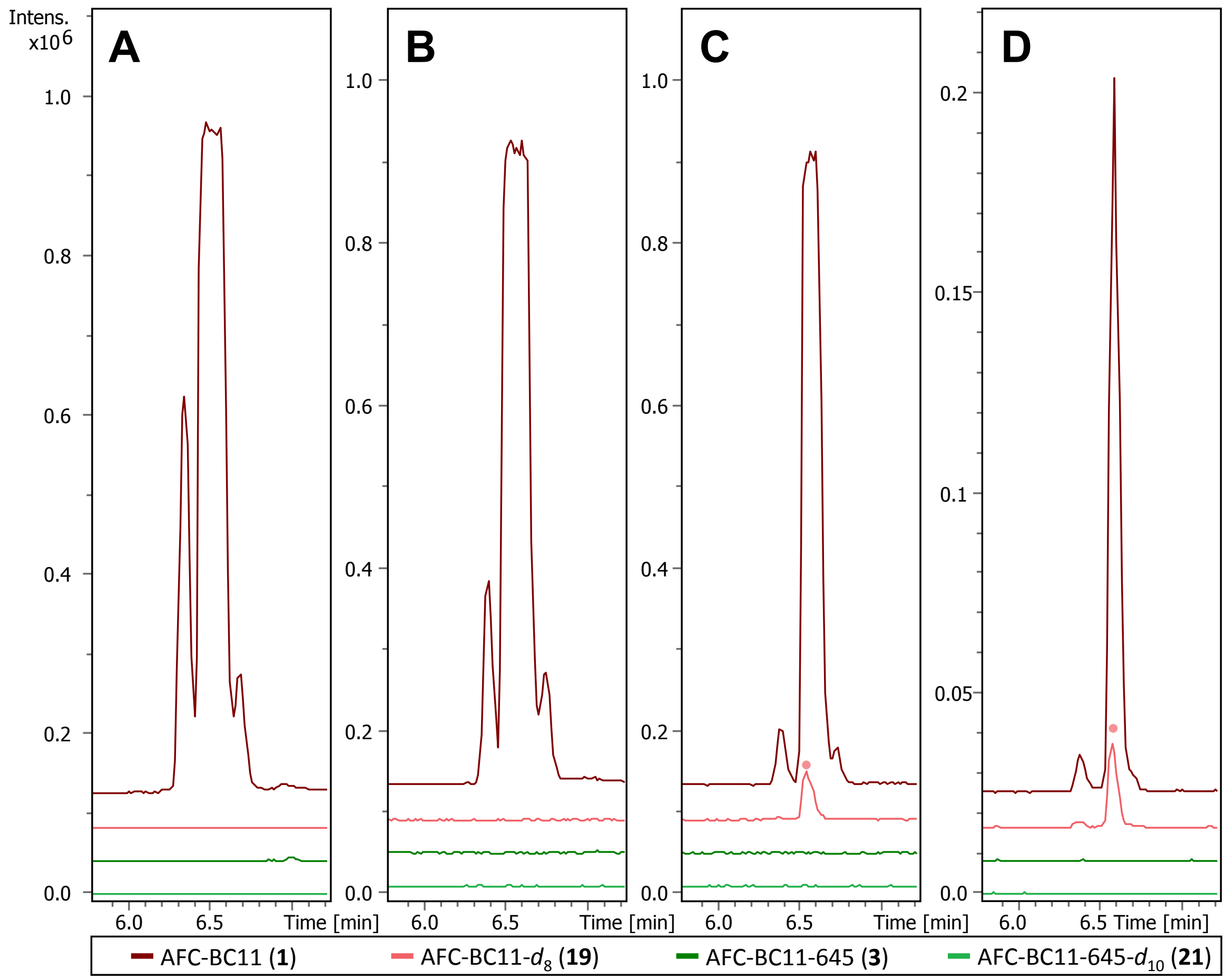

Figure S48. Extracted ion chromatograph of AFCs production following supplementation with *cis*-vaccenic acids. PDB cultures of wild-type K56-2 were treated with **(A)** DMSO, **(B)** *cis*-vaccenic acid, **(C)** *cis*-vaccenic acid: *cis*-vaccenic acid-d_{13}=1:1, and **(D)** *cis*-vaccenic acid-d_{13}. Peaks denoted with dots indicate deuterium-labeled AFC-BC11-d_8 (**19**; pink), no deuterium-labeled AFC intermediate AFC-645-d_{10} (**21** light green) was detected.

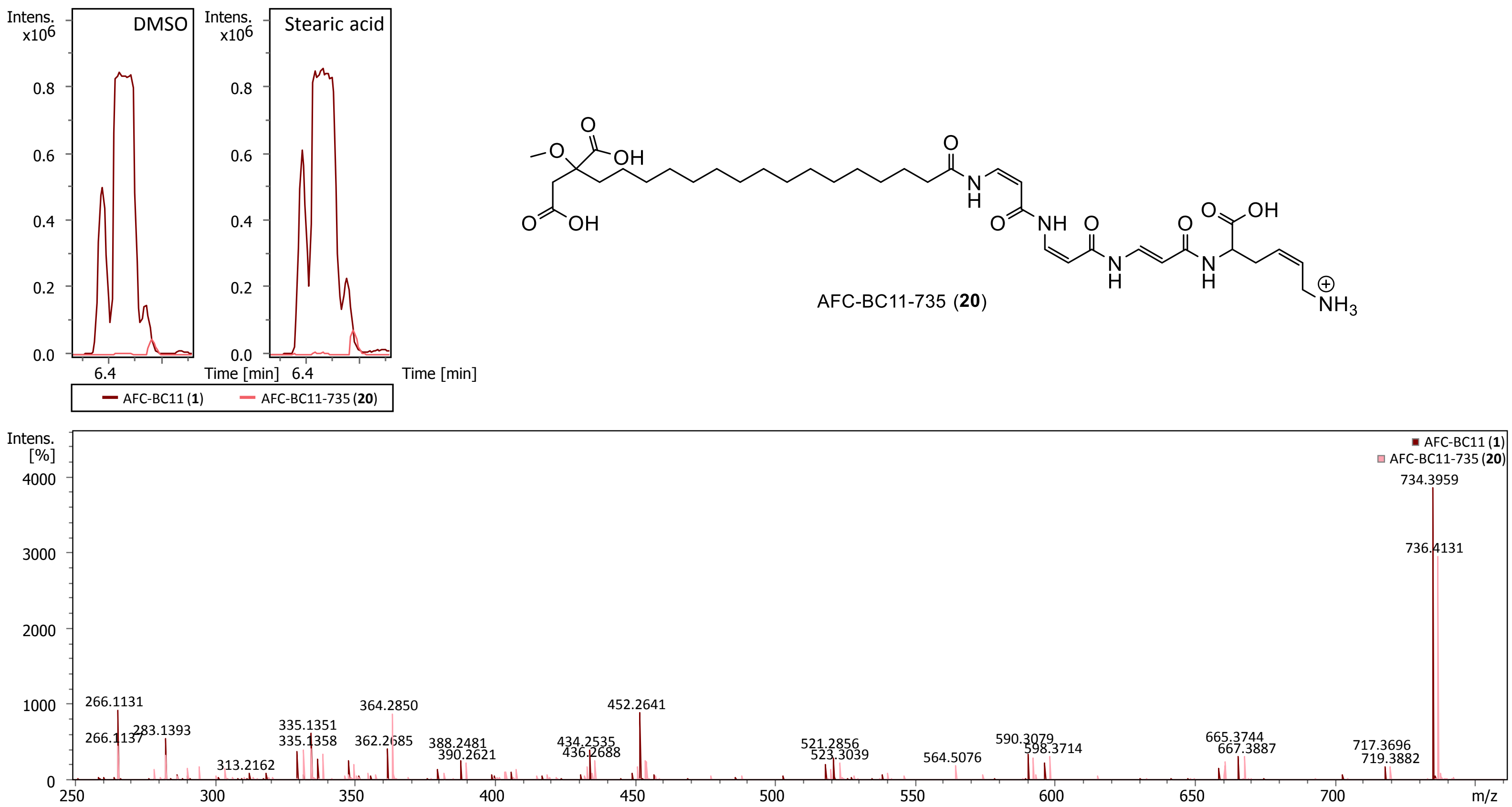

Figure S49. AFC-BC11-735 (**20**) production following supplementation with stearic acid in PDB. (**A**) Extracted ion chromatograph of AFC-BC11-735 (**20**) in DMSO control and stearic acid-supplemented cultures. (**B**) Structure of the AFC-BC11-735 (**20**). (**C**) HR-

MS/MS fragmentation comparison between AFC-BC11 (**1**) and AFC-BC11-735 (**20**), showing a +2H shift in the acyl side-chain fragments

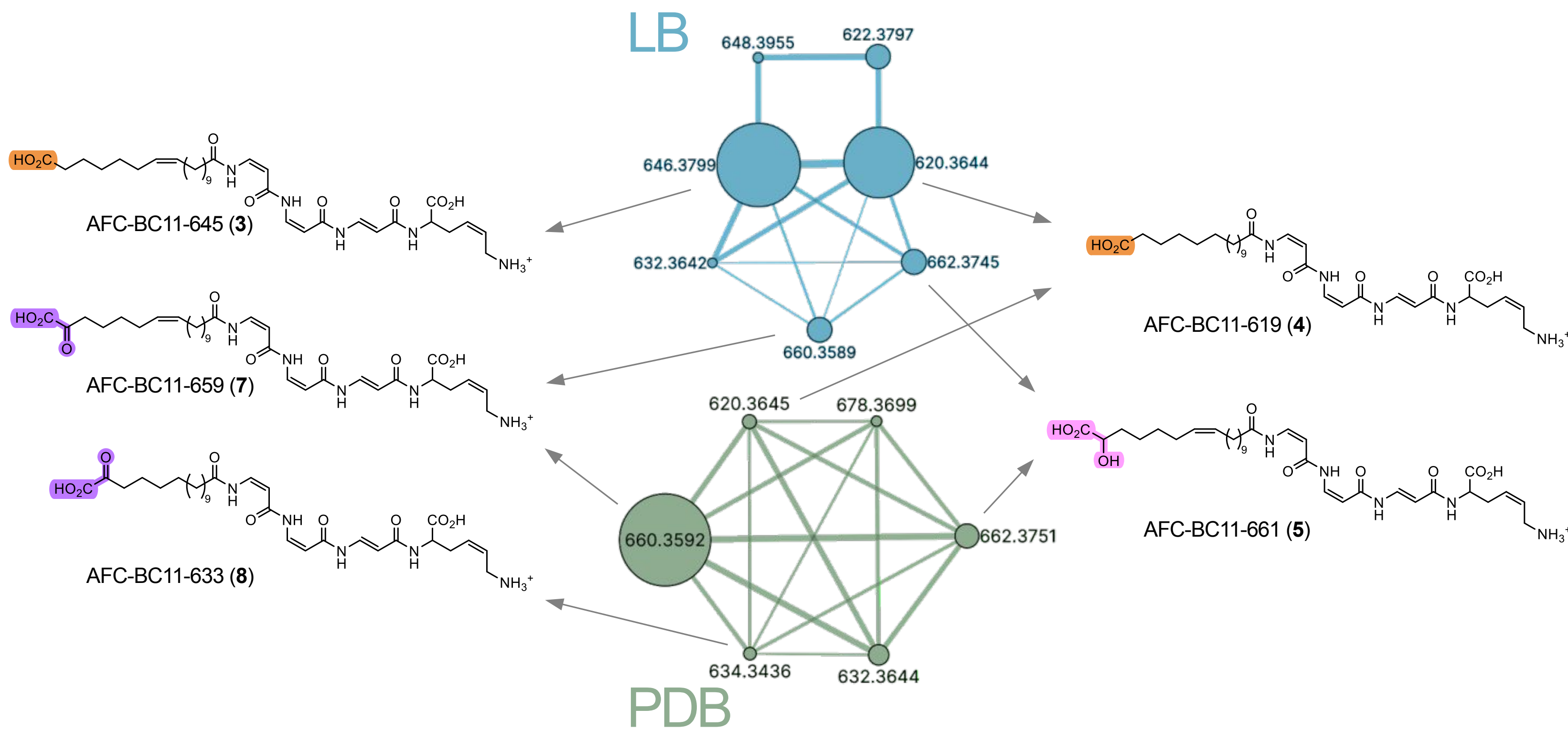

Figure S50. Molecular networking analysis of the *afcF* mutant cultured in LB and PDB media, highlighting the network of AFC-BC11 (**1**) and its derivatives. Node size corresponds to the peak area of each compound. Proposed structures of main derivatives are illustrated,

with the functional groups on the aliphatic side chain (ω-CFA, α-hydroxy ω-CFA and α-oxo ω-CFA) highlighted in orange, pink and purple, respectively.

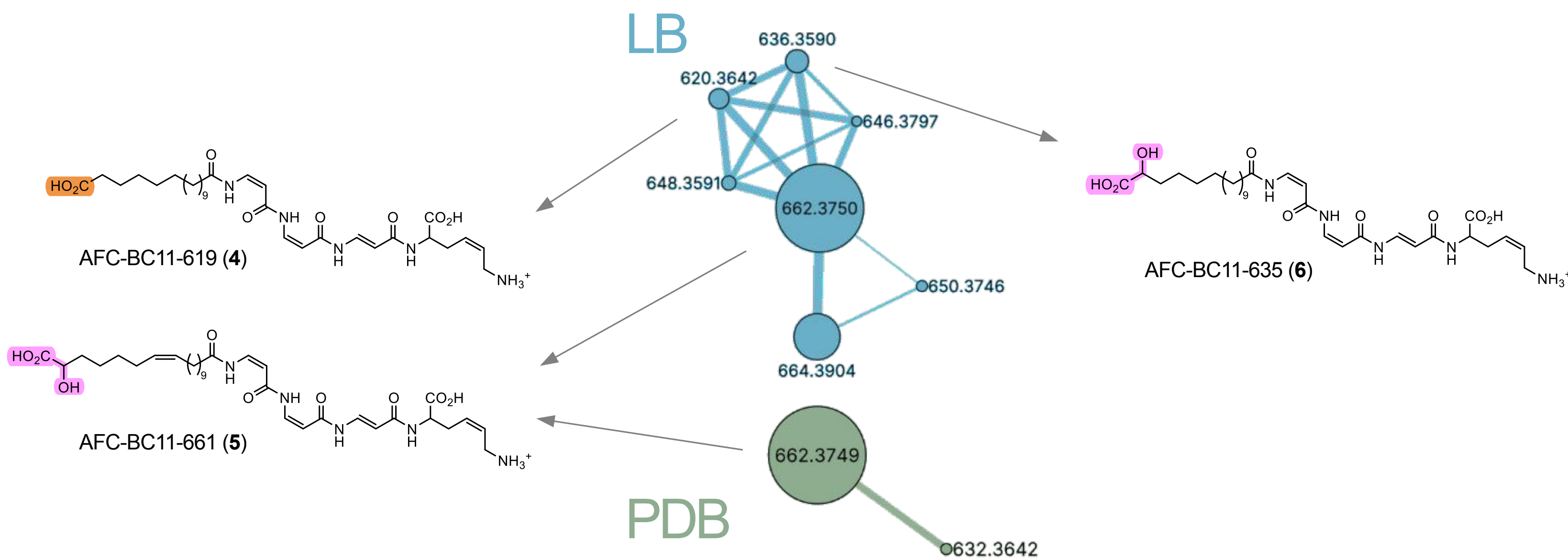

Figure S51. Molecular networking analysis results of the *afcS* mutant cultured in LB and PDB medium, highlighting the network of AFC-BC11 (**1**) and its derivatives. Node size corresponds to the peak area of each compound. Proposed structures of the main derivatives are illustrated. Functional groups on the aliphatic side chain (ω-CFA, α-hydroxy ω-CFA) are highlighted in orange and pink, respectively. The non-reduced bond on lysine at the C-terminus is highlighted in yellow.

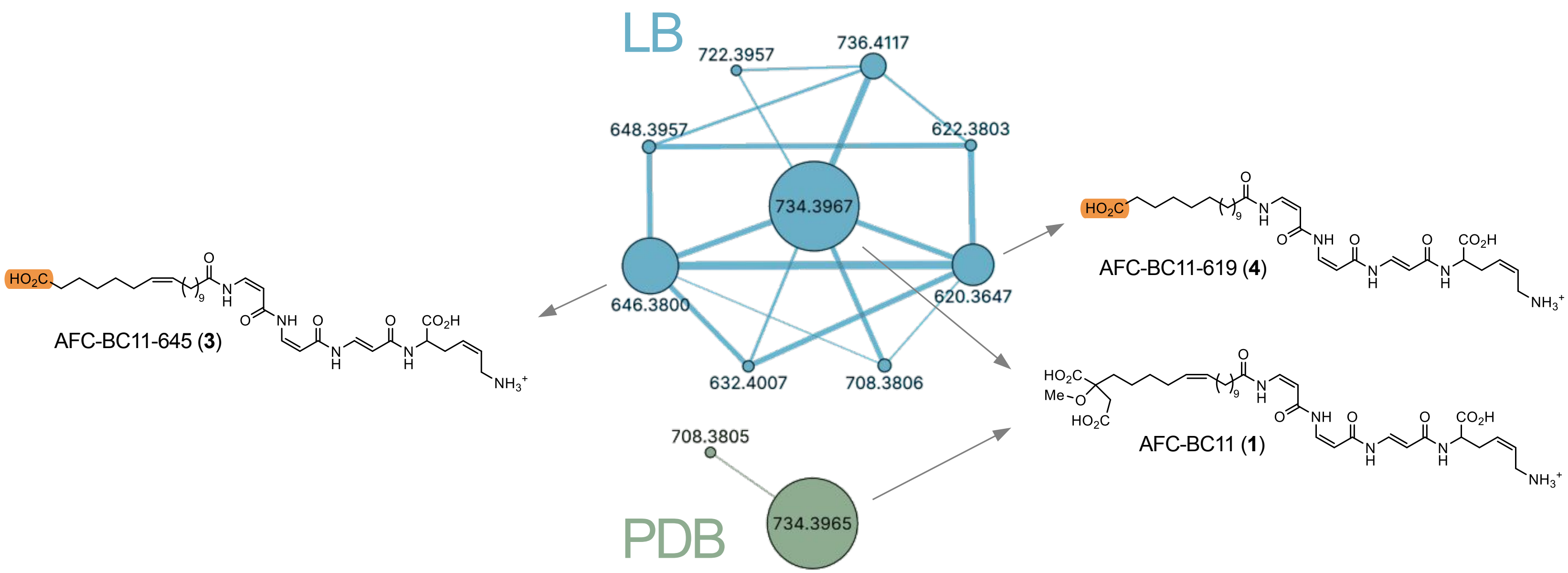

Figure S52. Molecular networking analysis results of the wildtype sample cultured in LB and PDB medium, highlighting the network of AFC-BC11 (**1**) and its derivatives. Node size corresponds to the peak area of each compound. Proposed structures of the main derivatives are illustrated. The carboxylic acid functional group on the aliphatic side chain is highlighted in orange.

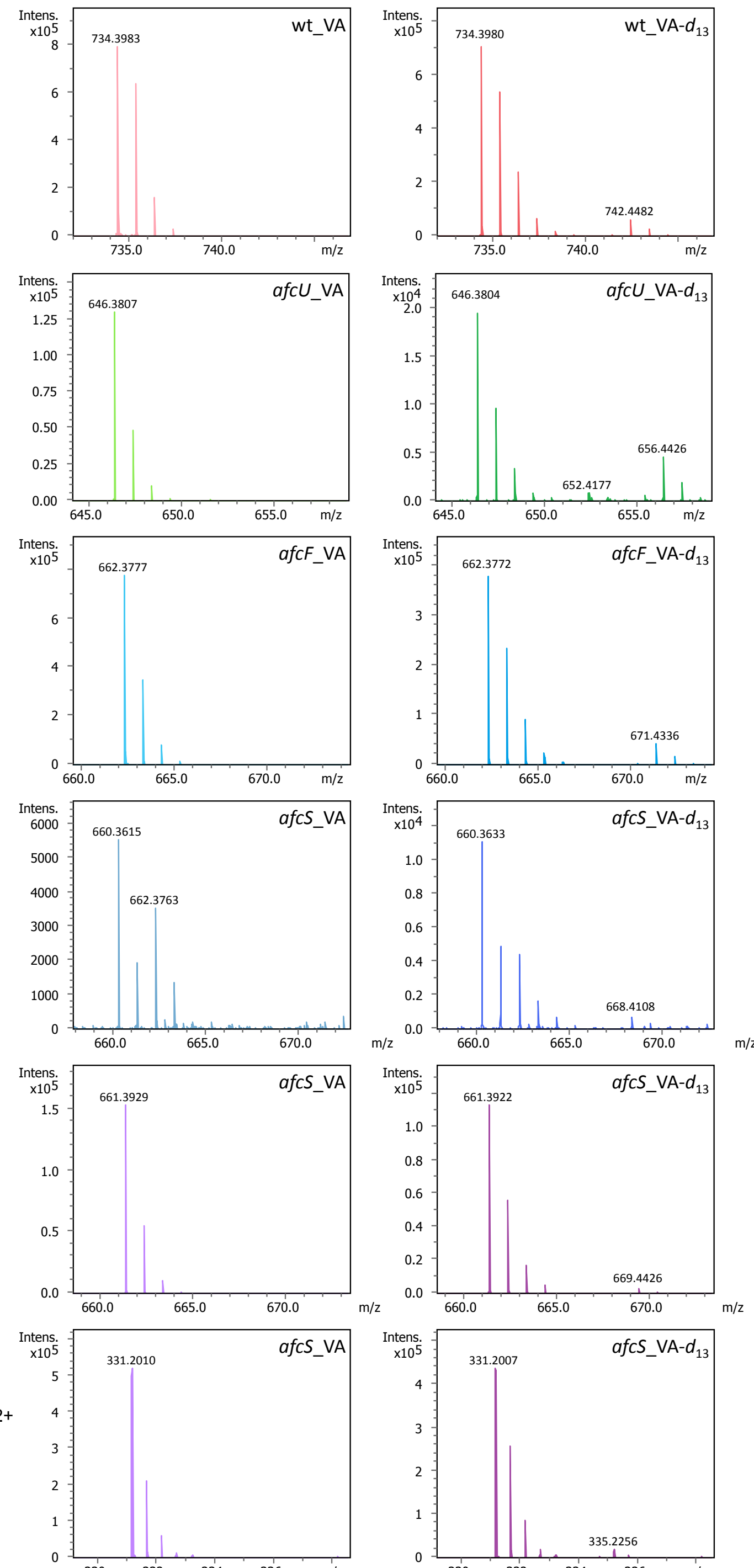

[AFC-BC11+H]$^+$
$C_{36}H_{56}N_5O_{11}^+$
m/z: 734.39708
Δppm = 1.7

[AFC-BC11-*d*$_8$+H]$^+$
$C_{36}H_{48}D_8N_5O_{11}^+$
m/z: 742.44730
Δppm = 1.2

[AFC-BC11-645+H]$^+$
$C_{33}H_{52}N_5O_8^+$
m/z: 646.38104
Δppm = 0.5

[AFC-BC11-645-*d*$_{10}$+H]$^+$
$C_{33}H_{42}D_{10}N_5O_8^+$
m/z: 656.44381
Δppm = −1.8

[AFC-BC11-661+H]$^+$
$C_{33}H_{52}N_5O_9^+$
m/z: 662.37595
Δppm = 2.6

[AFC-BC11-661-*d*$_9$+H]$^+$
$C_{33}H_{43}D_9N_5O_9^+$
m/z: 671.43245
Δppm = 1.7

[AFC-BC11-659+H]$^+$
$C_{33}H_{50}N_5O_9^+$
m/z: 660.36030
Δppm = 1.8

[AFC-BC11-659-*d*$_8$+H]$^+$
$C_{33}H_{42}D_8N_5O_9^+$
m/z: 668.41052
Δppm = 0.4

[AFC-BC11-660+H]$^+$
$C_{33}H_{53}N_6O_8^+$
m/z: 661.39194
Δppm = 1.5

[AFC-BC11-660-*d*$_8$+H]$^+$
$C_{33}H_{45}D_8N_6O_8^+$
m/z: 669.44215
Δppm = 0.7

[AFC-BC11-660+2H]$^{2+}$
$C_{33}H_{54}N_6O_8^{2+}$
m/z: 331.19961
Δppm = 4.2

[AFC-BC11-660-*d*$_8$+2H]$^{2+}$
$C_{33}H_{46}D_8N_6O_8^{2+}$
m/z: 335.22472
Δppm = 2.6

Figure S53. HRMS identification of deuterium-labeled AFCs following supplementation with *cis*-vaccenic acid-d_{13}. AFC compounds were detected in cultures of wild-type, Δ*afcU*, Δ*afcF* and Δ*afcS* strains grown in PDB medium with either *cis*-vaccenic acid or *cis*-vaccenic acid-d_{13}.

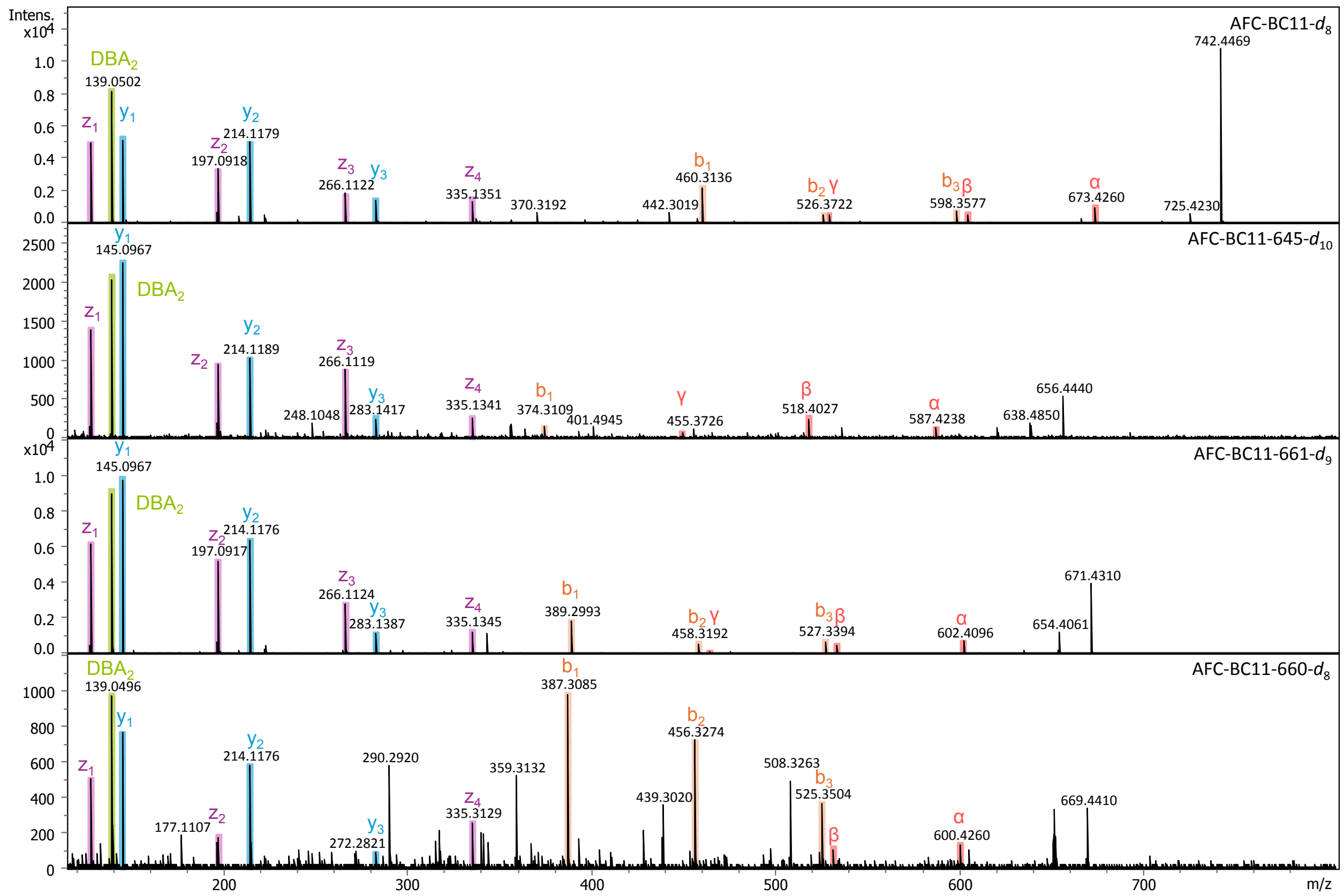

Intens.
x10^4
AFC-BC11-d8
DBA2
139.0502
742.4469
z1
y1
y2
214.1179
z2
197.0918
z3
266.1122
y3
z4
335.1351
370.3192
442.3019
b1
460.3136
b2 γ
526.3722
b3β
598.3577
α
673.4260
725.4230
AFC-BC11-645-d10
y1
145.0967
DBA2
z1
y2
214.1189
z2
z3
266.1119
248.1048
y3
283.1417
z4
335.1341
b1
374.3109
401.4945
γ
455.3726
β
518.4027
α
587.4238
638.4850
656.4440
AFC-BC11-661-d9
x10^4
y1
145.0967
DBA2
y2
214.1176
z1
z2
197.0917
z3
266.1124
y3
283.1387
z4
335.1345
b1
389.2993
b2 γ
458.3192
b3β
527.3394
α
602.4096
654.4061
671.4310
AFC-BC11-660-d8
DBA2
139.0496
y1
b1
387.3085
b2
456.3274
y2
214.1176
290.2920
359.3132
508.3263
z1
439.3020
b3
525.3504
177.1107
z2
y3
272.2821
z4
335.3129
β
α
600.4260
669.4410
m/z

Figure S54. HR-MS/MS fragmentation of deuterium-labeled AFCs. Deuterium-labeled AFC compounds were identified based on HRMS and HR-MS/MS fragmentation patterns, except for AFC-BC11-659-d_8 (**23**), which was only determined by HRMS.

References

(1) Flannagan, R. S.; Linn, T.; Valvano, M. A. A System for the Construction of Targeted Unmarked Gene Deletions in the Genus *Burkholderia*. *Environ Microbiol* **2008**, *10* (6), 1652–1660. https://doi.org/10.1111/j.1462-2920.2008.01576.x.

(2) Agnoli, K.; Freitag, R.; Gomes, M. C.; Jenul, C.; Suppiger, A.; Mannweiler, O.; Frauenknecht, C.; Janser, D.; Vergunst, A. C.; Eberl, L. Use of Synthetic Hybrid Strains to Determine the Role of Replicon 3 in Virulence of the *Burkholderia Cepacia* Complex. *Appl Environ Microbiol* **2017**, *83* (13), e00461-17. https://doi.org/10.1128/AEM.00461-17.

(3) Toyofuku, M.; Cárcamo-Oyarce, G.; Yamamoto, T.; Eisenstein, F.; Hsiao, C.-C.; Kurosawa, M.; Gademann, K.; Pilhofer, M.; Nomura, N.; Eberl, L. Prophage-Triggered Membrane Vesicle Formation through Peptidoglycan Damage in *Bacillus Subtilis*. *Nat Commun* **2017**, *8* (1), 481. https://doi.org/10.1038/s41467-017-00492-w.

(4) Turnbull, L.; Toyofuku, M.; Hynen, A. L.; Kurosawa, M.; Pessi, G.; Petty, N. K.; Osvath, S. R.; Cárcamo-Oyarce, G.; Gloag, E. S.; Shimoni, R.; Omasits, U.; Ito, S.; Yap, X.; Monahan, L. G.; Cavaliere, R.; Ahrens, C. H.; Charles, I. G.; Nomura, N.; Eberl, L.; Whitchurch, C. B. Explosive Cell Lysis as a Mechanism for the Biogenesis of Bacterial Membrane Vesicles and Biofilms. *Nat Commun* **2016**, *7* (1), 11220. https://doi.org/10.1038/ncomms11220.

(5) Chen, Y.-C.; Kalawong, R.; Toyofuku, M.; Eberl, L. The Role of Peptidoglycan Hydrolases in the Formation and Toxicity of *Pseudomonas Aeruginosa* Membrane Vesicles. *microLife* **2022**, *3*, uqac009. https://doi.org/10.1093/femsml/uqac009.

(6) Zhong, L.; Mühlenweg, A.; Hong, D.; Yammine, S.; Poch, A.; Xu, D.; Kirimlioglu, Y.; Großgloß, L.; Boulanger, M.; Graeger, F.; Seidel, M.; Gemander, M.; Walther, G.; Kemper, S.; Dang, T.; Royer, M.; Mainz, A.; Cociancich, S.; Süssmuth, R. D. The Intricate Nonribosomal Assembly of a Potent Antifungal Lipopeptide from the *Burkholderia Cepacia* Complex. *J Am Chem Soc* **2025**. https://doi.org/10.1021/jacs.5c04167.

(7) O'Grady, E. P.; Nguyen, D. T.; Weisskopf, L.; Eberl, L.; Sokol, P. A. The *Burkholderia Cenocepacia* LysR-Type Transcriptional Regulator ShvR Influences Expression of Quorum-Sensing, Protease, Type II Secretion, and *Afc* Genes. *J Bacteriol* **2011**, *193* (1), 163–176. https://doi.org/10.1128/JB.00852-10.

(8) Jenul, C.; Sieber, S.; Daeppen, C.; Mathew, A.; Lardi, M.; Pessi, G.; Hoepfner, D.; Neuburger, M.; Linden, A.; Gademann, K.; Eberl, L. Biosynthesis of Fragin Is Controlled by a Novel Quorum Sensing Signal. *Nat Commun* **2018**, *9* (1), 1297. https://doi.org/10.1038/s41467-018-03690-2.

(9) Pluskal, T.; Castillo, S.; Villar-Briones, A.; Oresic, M. MZmine 2: Modular Framework for Processing, Visualizing, and Analyzing Mass Spectrometry-Based Molecular Profile Data. *BMC Bioinformatics* **2010**, *11*, 395. https://doi.org/10.1186/1471-2105-11-395.

(10) Heuckeroth, S.; Damiani, T.; Smirnov, A.; Mokshyna, O.; Brungs, C.; Korf, A.; Smith, J. D.; Stincone, P.; Dreolin, N.; Nothias, L. F.; Hyötyläinen, T.; Orešič, M.; Karst, U.; Dorrestein, P. C.; Petras, D.; Du, X.; Hooft, J. J. J. van der; Schmid, R.; Pluskal, T. Reproducible Mass Spectrometry Data Processing and Compound Annotation in MZmine 3. *Nat Protoc* **2024**, *19* (9), 2597–2641. https://doi.org/10.1038/s41596-024-00996-y.

(11) Schmid, R.; Heuckeroth, S.; Korf, A.; Smirnov, A.; Myers, O.; Dyrlund, T. S.; Bushuiev, R.; Murray, K. J.; Hoffmann, N.; Lu, M.; Sarvepalli, A.; Zhang, Z.; Fleischauer, M.; Dührkop, K.; Wesner, M.; Hoogstra, S. J.; Rudt, E.; Mokshyna, O.; Brungs, C.; Ponomarov, K.; Mutabdžija, L.; Damiani, T.; Pudney, C. J.; Earll, M.; Helmer, P. O.; Fallon, T. R.; Schulze, T.; Rivas-Ubach, A.; Bilbao, A.; Richter, H.; Nothias, L.-F.; Wang, M.; Orešič, M.; Weng, J.-K.; Böcker, S.; Jeibmann, A.; Hayen, H.; Karst, U.; Dorrestein, P. C.; Petras, D.; Du, X.; Pluskal, T. Integrative Analysis of Multimodal Mass Spectrometry Data in MZmine 3. *Nat Biotechnol* **2023**, *41* (4), 447–449. https://doi.org/10.1038/s41587-023-01690-2.

(12) Fotie, J. The Potential of Peptides and Depsipeptides from Terrestrial and Marine Organisms in the Fight against Human Protozoan Diseases. In *Bioactive Natural Products*; John Wiley & Sons, Ltd, 2014; pp 279–320. https://doi.org/10.1002/9783527684403.ch10.

(13) Portmann, C.; Blom, J. F.; Kaiser, M.; Brun, R.; Jüttner, F.; Gademann, K. Isolation of Aerucyclamides C and D and Structure Revision of Microcyclamide 7806A: Heterocyclic Ribosomal Peptides from *Microcystis Aeruginosa* PCC 7806 and Their Antiparasite Evaluation. *J Nat Prod* **2008**, *71* (11), 1891–1896. https://doi.org/10.1021/np800409z.

(14) Takahashi, H.; Iwasaki, A.; Ebihara, A.; Taguchi, R.; Jeelani, G.; Nozaki, T.; Suenaga, K. Isolation of Hennaminal and Isolation and Total Synthesis of Hennamide, Pyrrolinone Compounds from the Marine *Cyanobacterium Rivularia* Sp. *Org Lett* **2023**, *25* (14), 2400–2404. https://doi.org/10.1021/acs.orglett.3c00421.

(15) Miller, V. L.; Mekalanos, J. J. A Novel Suicide Vector and Its Use in Construction of Insertion Mutations: Osmoregulation of Outer Membrane Proteins and Virulence Determinants in *Vibrio Cholerae* Requires toxR. *J Bacteriol* **1988**, *170* (6), 2575–2583. https://doi.org/10.1128/jb.170.6.2575-2583.1988.

(16) Hanahan, D. Studies on Transformation of *Escherichia Coli* with Plasmids. *J Mol Biol* **1983**, *166* (4), 557–580. https://doi.org/10.1016/S0022-2836(83)80284-8.

(17) Casadaban, M. J.; Cohen, S. N. Analysis of Gene Control Signals by DNA Fusion and Cloning in *Escherichia Coli*. *J Mol Biol* **1980**, *138* (2), 179–207. https://doi.org/10.1016/0022-2836(80)90283-1.

(18) Vanlaere, E.; Baldwin, A.; Gevers, D.; Henry, D.; De Brandt, E.; LiPuma, J. J.; Mahenthiralingam, E.; Speert, D. P.; Dowson, C.; Vandamme, P. Taxon K, a Complex within the *Burkholderia Cepacia* Complex, Comprises at Least Two Novel Species, *Burkholderia Contaminans* Sp. Nov. and *Burkholderia Lata* Sp. Nov. *Int J Syst Evol Microbiol* **2009**, *59* (Pt 1), 102–111. https://doi.org/10.1099/ijs.0.001123-0.

(19) Van, V. T.; Berge, O.; Balandreau, J.; Ké, S. N.; Heulin, T. Isolement et activité nitrogénasique de *Burkholderia vietnamiensis*, bactérie fixatrice d'azote associée au riz

(Oryza sativa L) cultivé sur un sol sulfaté du Viêt-nam. *Agronomie* **1996**, *16* (8), 479–491. https://doi.org/10.1051/agro:19960802.
(20) Wallner, A.; Moulin, L.; Busset, N.; Rimbault, I.; Béna, G. Genetic Diversity of Type 3 Secretion System in *Burkholderia* s.l. and Links with Plant Host Adaptation. *Front Microbiol* **2021**, *12*. https://doi.org/10.3389/fmicb.2021.761215.
(21) Brett, P. J.; DeShazer, D.; Woods, D. E. *Burkholderia Thailandensis* Sp. Nov., a *Burkholderia Pseudomallei*-like Species. *Int J Syst Bacteriol* **1998**, *48 Pt 1*, 317–320. https://doi.org/10.1099/00207713-48-1-317.
(22) Martina, P.; Leguizamon, M.; Prieto, C. I.; Sousa, S. A.; Montanaro, P.; Draghi, W. O.; Stämmler, M.; Bettiol, M.; de Carvalho, C. C. C. R.; Palau, J.; Figoli, C.; Alvarez, F.; Benetti, S.; Lejona, S.; Vescina, C.; Ferreras, J.; Lasch, P.; Lagares, A.; Zorreguieta, A.; Leitão, J. H.; Yantorno, O. M.; Bosch, A. *Burkholderia Puraquae* Sp. Nov., a Novel Species of the *Burkholderia Cepacia* Complex Isolated from Hospital Settings and Agricultural Soils. *Int J Syst Evol Microbiol* **2018**, *68* (1), 14–20. https://doi.org/10.1099/ijsem.0.002293.
(23) Gotschlich, A.; Huber, B.; Geisenberger, O.; Tögl, A.; Steidle, A.; Riedel, K.; Hill, P.; Tümmler, B.; Vandamme, P.; Middleton, B.; Camara, M.; Williams, P.; Hardman, A.; Eberl, L. Synthesis of Multiple *N*-Acylhomoserine Lactones Is Wide-Spread among the Members of the *Burkholderia Cepacia* Complex. *Syst Appl Microbiol* **2001**, *24* (1), 1–14. https://doi.org/10.1078/0723-2020-00013.
(24) Subramoni, S.; Agnoli, K.; Eberl, L.; Lewenza, S.; Sokol, P. A. Role of *Burkholderia Cenocepacia afcE* and *afcF* Genes in Determining Lipid-Metabolism-Associated Phenotypes. *J Gen Microbiol* **2013**, *159* (Pt_3), 603–614. https://doi.org/10.1099/mic.0.064683-0.
(25) Figurski, D. H.; Helinski, D. R. Replication of an Origin-Containing Derivative of Plasmid RK2 Dependent on a Plasmid Function Provided in Trans. *Proc Natl Acad Sci USA* **1979**, *76* (4), 1648–1652. https://doi.org/10.1073/pnas.76.4.1648.
(26) Kovach, M. E.; Elzer, P. H.; Steven Hill, D.; Robertson, G. T.; Farris, M. A.; Roop, R. M.; Peterson, K. M. Four New Derivatives of the Broad-Host-Range Cloning Vector pBBR1MCS, Carrying Different Antibiotic-Resistance Cassettes. *Gene* **1995**, *166* (1), 175–176. https://doi.org/10.1016/0378-1119(95)00584-1.
(27) Horton, R. M.; Cai, Z.; Ho, S. N.; Pease, L. R. Gene Splicing by Overlap Extension: Tailor-Made Genes Using the Polymerase Chain Reaction. *Biotechnol Adv* **2013**, *54* (3), 129–133. https://doi.org/10.2144/000114017.
(28) Dashti, Y.; Nakou, I. T.; Mullins, A. J.; Webster, G.; Jian, X.; Mahenthiralingam, E.; Challis, G. L. Discovery and Biosynthesis of Bolagladins: Unusual Lipodepsipeptides from *Burkholderia Gladioli* Clinical Isolates. *Angew Chem Int Ed* **2020**, *59* (48), 21553–21561. https://doi.org/10.1002/anie.202009110.
(29) Gilchrist, C. L. M.; Chooi, Y.-H. Clinker & Clustermap.Js: Automatic Generation of Gene Cluster Comparison Figures. *Bioinformatics* **2021**, *37* (16), 2473–2475. https://doi.org/10.1093/bioinformatics/btab007.
(30) Dose, B.; Ross, C.; Niehs, S. P.; Scherlach, K.; Bauer, J. P.; Hertweck, C. Food-Poisoning Bacteria Employ a Citrate Synthase and a Type II NRPS to Synthesize Bolaamphiphilic

Lipopeptide Antibiotics. *Angew Chem Int Ed* **2020**, *59* (48), 21535–21540. https://doi.org/10.1002/anie.202009107.
